# Medical Error: Using Storytelling and Reflection to Impact Resident Error Response Factors

**DOI:** 10.15766/mep_2374-8265.11451

**Published:** 2024-10-10

**Authors:** Sherry Adkins, Peter Reynolds, Kelly Rabah, Stacy Flowers

**Affiliations:** 1 Associate Program Director, Department of Family Medicine, Wright State University Boonshoft School of Medicine, Family Health Services of Darke County, Inc.; 2 Residency Program Director, Department of Family Medicine, Wright State University Boonshoft School of Medicine; 3 Director of Patient Safety and Quality Improvement and Assistant Professor, Wright State University Boonshoft School of Medicine; Senior Director of Quality Innovation, Wright State Physicians; 4 Associate Professor and Director of Behavioral Science, Department of Family Medicine, Wright State University Boonshoft School of Medicine

**Keywords:** Medical Error, Storytelling, Psychological Adaptation, Quality Improvement/Patient Safety, Reflection/Narrative Medicine, Family Medicine

## Abstract

**Introduction:**

Medical error is common and has a significant impact on physicians, learners, and patients’ perception of the medical system; however, residents receive little formal training on this topic. This curriculum aims to foster sharing of personal medical error stories, review and practice error management and coping strategies, and impact error response factors.

**Methods:**

Faculty identified factors related to effective physician error management and recovery in order to develop a targeted curriculum for family medicine residents. The curriculum consisted of three 1-hour didactic sessions in a medium-sized, urban program. Instructional methods included guided reflection after mentor storytelling, small-group discussion, role-play, and self-reflection.

**Results:**

Twenty-two out of 30 (73%) residents completed the premodule survey, and 15 out of 30 (50%) residents completed the post module survey. Fewer than half of residents reported they knew what to do when faced with medical error, but this increased to 93% after curriculum delivery, as did rates of reported error story sharing. Resident reported self-efficacy (*I can be honest about the errors I make as a doctor.*) and self-awareness (*I acknowledge when I am at increased risk for making errors*) also increased following the curriculum.

**Discussion:**

Family medicine residents are receptive to learning from peers and mentors about error management and recovery. A brief curriculum can impact the culture around disclosure and support. Future iterations should focus on the impact of targeted curricular interventions on patient-oriented outcomes related to medical error.

## Educational Objectives

By the end of this activity, learners will be able to:

1. Define medical error and integrate this topic into their understanding of the profession of medicine.
2. Identify ways that physicians cope and thrive after medical error.
3. Describe *safety culture* and identify ways that colleagues can help with error management and recovery.
4. Describe local policies and practices related to medical error.

## Introduction

*I am a healer, yet sometimes I do more harm than good.* **D. Hilfiker**, “Facing Our Mistakes”

The Kohn et al. landmark study in 2000 reported preventable medical errors in hospitals resulted in approximately 98,000 deaths across 33.6 million hospital admissions.^1^ More recent studies estimate that 440,000 people die in the United States each year due to preventable medical error.^2^ Negative outcomes related to error are evident: rising health care costs due to adverse events and the subsequent need for repeat tests, readmissions, increased length of stay, rising insurance premiums, and the American public's lack of trust in health care professionals and institutions. Other indirect costs that result from poor safety outcomes in the US include loss of income to patients, and sometimes-preventable intermediate or long-range disabilities or chronic health care challenges.

Even though medical error is the third leading cause of death in the US, costing between $73.5 and $98 billion in quality adjusted life years,^1,3^ and error experiences are common among residents,^4^ residents receive little formal training in error management and recovery.

Maladaptive coping strategies appear frequently among learners^4,5^ and practicing physicians, with impact on physician quality of life and patient care.^6^ Researchers have identified common physician trajectories after error,^7^ including the frequently reported phenomenon of second victimhood—fear of litigation, shame, self-blame, and guilt arising from acute awareness of human fallibility and its impact on patients. These consequences appear cumulative and build across one's tenure. Further, shame and embarrassment create barriers to disclosure, reducing opportunity for analysis and process improvement.^8^ Second victimhood recovery follows six stages: chaos and accident response, intrusive reflections, restoring personal integrity, enduring the inquisition, obtaining emotional first aid, and moving on (which can involve surviving in, thriving in, or dropping out of medicine).^7^

In contrast to maladaptive responses, speaking up about medical error is an important act that impacts patient safety, quality of patient care, and long-term error reduction as transparency improves.^8^ The act of disclosure can be healing for the physician as it provides the opportunity to connect with the patient in a meaningful way where care and compassion is expressed, and patient-centered care is the goal.

Researchers have identified several factors associated with desired outcomes: error reporting,^9–11^ disclosure,^12^ coping,^13,14^ constructive change,^14^ and growth after error.^15^ More specifically, coping may be aided by professional counseling, discussing the error with trusted peers, and engaging in quality projects linked to the error. Coping is essential to safeguard the emotional well-being of physicians and to prevent burnout. Error management support and guidance from more experienced physicians impacts resident emotions and behaviors. Presumably, supporting and promoting resident exposure to medical error disclosure through engagement with more experienced physicians helps them to emulate constructive responses and to identify maladaptive ones.

Previous curricular evaluations have demonstrated the value of simulated clinical scenarios to practice communication-based skills in a safe setting using a team approach.^16–18^ Our curriculum contributes to the existing literature by exploring the dimension of storytelling. Storytelling creates the framework to engage residents in the discussion and practice of disclosure, and to integrate a challenging concept—personal fallibility—into their professional identity.

To summarize, development of an approach to medical error is critical for personal and professional resilience^19^ and meaningful participation in quality improvement.^7,20^ Curricula addressing error management and recovery are desired by learners^21,22^ and can improve resident knowledge, skills, and abilities in this area.^10,11,23^ Mentor storytelling can be particularly effective^24^ as well as integrating error management and recovery into everyday activities.^10^

## Methods

We organized key factors related to effective error management and physician growth after error using a logic model for developing health interventions called the predisposing, reinforcing, and enabling constructs in educational diagnosis and evaluation - policy, regulatory, and organizational constructs in educational and environmental development (PRECEDE-PROCEED) model.^25^ Predisposing factors included resident knowledge (awareness of mentor error, local policies and procedures, effective error disclosure steps, and related professional values) and beliefs (error recovery self-efficacy and attitude towards error). We also targeted enabling factors, specifically resident skills (error and cause identification, error disclosure, emotion management, and accessing support), as well as a reinforcing factor (talking among colleagues). The facilitator's guide (Appendix A) includes additional details on the medical error response factors.

The primary goal of our curriculum was to help residents appreciate the pervasiveness of medical error and practice productive error responses. Preexisting error curricula in our family medicine residency program included: (1) the Institute for Healthcare Improvement's patient safety module completed during resident orientation^26,27^; (2) a longitudinal wellness program, Tending the Flame, with a session on medical error and a personal wellness plan (inclusive of coping strategies) development assignment^28^; and (3) a didactic session specifically on disclosure training (given once every 3 years). In addition, local rotation sites had policies related to medical error and disclosure although residents were not particularly aware of these before our curricular intervention.

We used the AAMC Quality Improvement and Patient Safety Competencies^29^ and ACGME family medicine milestones^30^ to inform the development of the educational objectives and pedagogical techniques (i.e., facilitating reflection and role-modeling for affective or attitudinal targets like self-efficacy). Specific objectives are included in our facilitator's guide (Appendix A), which also describes the implementation for the three 60-minute resident sessions. Slides for each session are included in Appendices B–F, and the faculty survey (Appendix G) and pre-/postmodule surveys (Appendices H & I) are also included.

### Logistics

We recruited family medicine residency faculty (*N* = 7) in a medium-sized, urban, midwestern program with an e-mail and survey (Appendix G) sent 1 week before session one. During weekly didactics we recruited PGY 1, 2, and 3 family medicine residents (*N* = 30). We scheduled the three sessions to occur over a 5 months period between August 2022 and January 2023, in order to allow participants to have time to process the session content in between sessions.

### Session One

We used mentor storytelling to demonstrate physician error, emotions/coping strategies, and the ways in which the physician's environment can impact error response (e.g., workload and error culture). Prior to the session, we identified a faculty volunteer to share a personal story of an error and the aftermath with our program recruitment and faculty survey (Appedix G). Once the faculty member was selected, we gave the following guidance to potential storytellers: (1) we believe the most impactful stories will come from program mentors and leadership; (2) the session is confidential and for learning; (3) the purpose of the story sharing is to reflect on ways that physicians react to medical error, amplifying strategies that result in good care for the patient and physician(s) involved; (4) stories do not have to be long, 5–10 minutes works well. The session (Appendix B) began with the faculty story and then the faculty facilitator guided residents in reflection using several prompts (Appendix C). Next, the session facilitator presented a small amount of lecture material, facilitated small- and large-group discussions, and prompted timed self-reflection through writing.

### Session Two

During the next session (Appendix D), we stimulated interest by discussing key professional values related to error and then had a large-group discussion of learner fears and barriers related to error. We introduced the concept of *safety culture*, and residents had the opportunity to reflect on strengths and weaknesses of the current culture of their practice. The faculty presenter engaged learners throughout the session using small- and large-group discussion, polling, and timed self-reflection through writing.

### Session Three

During the third session (Appendix E), the faculty presenter led a review of local policies and procedures related to medical error, and residents practiced self-awareness, error disclosure, root cause reflection, and coping. The faculty member facilitated the review of sample error cases (Appendix F) and also instructed students to write a letter to a colleague or future self in the wake of perceived error.

### Evaluation

We emailed residents the premodule survey (Appendix H) immediately prior to the first session. Residents were given time to complete the premodule survey immediately prior to the start of the session, but survey links remained active through the start of session three to allow residents who missed session one or two the opportunity to complete the survey. The postmodule survey (Appendix I) was sent via email to residents at the end of session three, and residents were given time to complete this survey just after session three concluded. Anonymous pre- and postmodule survey responses were analyzed and compared. To measure knowledge and confidence we collated participant affirmative responses (*agree*/*strongly agree*) on a 5-point Likert scale (1 = *strongly disagree*, 5 = *strongly agree*). We measured satisfaction by collating a percentage of residents selecting each rating of the emotional difficulty (0 = *not emotionally difficult at all*, 10 = *extremely emotionally difficult*) and helpfulness (0 = *not helpful at all,* 10 = *extremely helpful*) of the curriculum. Due to small sample size, analyses by PGY were not undertaken. The evaluation procedures were approved by the Wright State University Boonshoft School of Medicine Institutional Review Board (FWA# 00002427, approved August 26, 2022).

## Results

Of the cohort of 30 PGY 1, 2, and 3 participating family medicine residents, 22 (73%) completed the premodule survey, and 15 (50%) completed the postmodule survey. Seven pre- and postmodule surveys were able to be matched using anonymous codes. Of those who completed the postmodule survey, nine completed all three sessions, five completed two sessions, and one completed one session. Additionally, seven out of seven faculty completed the faculty survey.

### State of Preexisting Environment

Premodule responses demonstrated the pervasiveness of error experience among residents. Most residents reported having experienced error (55%, *n* = 12) and having had a mentor or peer share an error story with them (73%, *n* = 16). Among those with an error experience, 10 (77%) reported the error was disclosed to the patient. Many residents with error experience reported that their team or organization learned from the error (75%, *n* = 9), acknowledged the error (92%, *n* = 11), and debriefed as a team (83%, *n* = 10).

### Knowledge and Confidence

**Figure 1.**
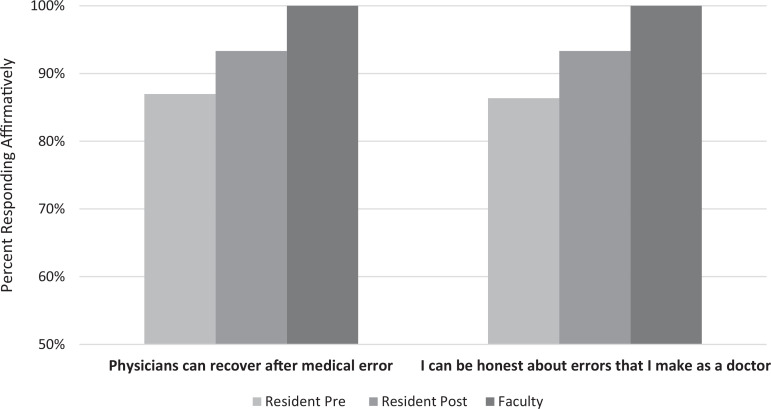
Faculty (*N* = 7) and resident premodule (*N* = 22) and postmodule (*N* = 15) beliefs about error, presented as percentage of respondents responding affirmatively (*agree*/*strongly agree*).

Our curriculum was associated with an increase in residents who reported several target factors: disclosure confidence/self-efficacy, knowledge of local procedures, accessing support as an error response, faculty and peer story sharing, and acknowledgement that mentors have made errors. Specifically, disclosure self-efficacy (*I can be honest about the errors I make as a doctor.*) increased after the curriculum from 86% (*n* = 19) to 93% (*n* = 14; Figure 1). As expected, faculty reported higher levels of confidence compared to residents with error disclosure and personal relationship recovery (Figure 1).

**Figure 2.**
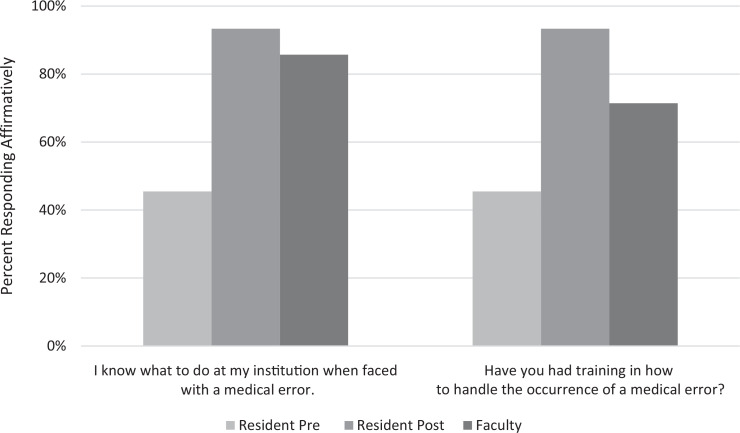
Knowledge of error procedures and error training among faculty (*N* = 7) and residents, both premodule (*N* = 22) and postmodule (*N* = 15), presented as percentage of respondents responding affirmatively (*agree*/*strongly agree*).

**Figure 3.**
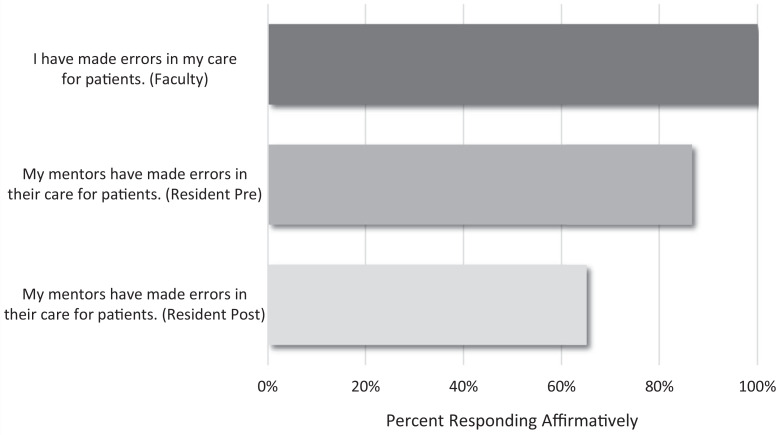
Resident premodule (*N* = 22) and postmodule (*N* = 15) perception of mentor error and faculty (*N* = 7) reporting of error, both presented as percent responding affirmatively (*Yes*).

Knowledge of related local procedures (*I know what to do at my institution when faced with a medical error.*) increased from 46% (*n* = 10) to 93% (*n* = 14; Figure 2). The curriculum was also associated with an increase in reaching out to others as an error response from 36% (*n* = 8) to 87% (*n* = 13). Debriefing with the team remained common, but the rate of residents reporting *feel bad about myself* as an error response increased from 41% (*n* = 9) to 60% (*n* = 9). After the curriculum, rates of reported faculty and peer story sharing increased, and resident reported awareness of mentor error increased from 68% (*n* = 15) to 87% (*n* = 13). Incidentally, all faculty respondents reported *I have made errors in my care for patients* (Figure 3). Responses also demonstrated an increase in resident reported self-awareness (*I acknowledge when I am at increased risk for making errors*) from 77% (*n* = 17) to 93% (*n* = 14).

### Satisfaction

**Figure 4.**
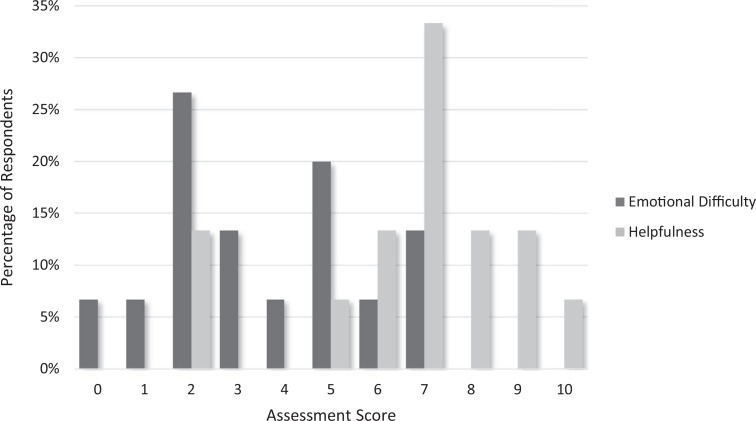
Resident postmodule (*N* = 15) assessment of the emotional difficulty (0 = *not emotionally difficult at all*, 10 = *extremely emotionally difficult*) and helpfulness (0 = *not helpful at all,* 10 = *extremely helpful*) of the curriculum, presented as percent respondents selecting each ranking.

Overall residents reported the training was helpful (ranking > 5), and six residents (40%) reported an emotionally difficult rating of 5 or greater for the curriculum (Figure 4). Prior to the module, residents were most interested in further training through personal stories of mentor error (73%, *n* = 16), and after the curriculum, residents reported most interest in additional training in legal and malpractice risk (73%, *n* = 11).

## Discussion

To address the lack of a graduate medical education curriculum related to medical error response, we developed three sessions for family medicine residents. We found the use of a model for factor organization and assessment of the curriculum as a necessary initial step. From this evaluation we confirmed that most residents already had prior experience with team or personal error, our curricular intervention positively impacted specific targets, and residents found the curriculum helpful. However, sample size and the format and timing of our postmodule survey limited our ability to fully assess our curriculum. We plan to refine the curriculum based on initial results by adding several new evaluation techniques and offering the module more broadly to other graduate medical education specialty programs in our community.

Because of the complexity of physician error response, we found the process of organizing predisposing, enabling, and reinforcing factors from the literature helpful for identifying potential curricular targets. Similarly, our exploration of the preexisting curriculum allowed us to select targets not already being fully addressed. Our residency, like many others, has a formal and informal curriculum (i.e., unspoken norms and lessons that residents learn) for medical error, and identifying this curriculum helped us partner with current faculty champions to develop a synergistic curriculum.

Our curriculum evaluation suggests that most residents had prior experience with team or personal error, confirming the importance of the topic, and suggests that a brief curriculum can be effective at impacting important error response factors. Residents reported significant interest in error training, and many found our curriculum helpful. The interest in further training shifting from personal stories of mentor error (premodule) to legal and malpractice concerns (postmodule) could indicate a deficiency in the curriculum or a natural change in focus.

From an experiential standpoint, the storytelling by faculty prompted residents to use empathic language to explore what they would have felt and done in the storyteller's shoes and voice the respect they had for the physician doing the right thing. After initial rumination regarding the error itself, residents shared surprising insights into error management and recovery, including a reframing of the error and a thankfulness for physician colleagues that can be called upon to help deal with complications of error. Based on our experience in this curriculum, residents can be remarkably open, especially when openness is modeled by mentors. Surprisingly, the rate of residents who report feeling bad about oneself after error increased from 41% (*n* = 9) to 60% (*n* = 9) after the curriculum. Perhaps the act of addressing this subject as a group was emotionally charging. The fact that 40% of residents reported an emotionally difficult rating of 5 or greater highlights the importance of including resident support resources during the sessions themselves. We are unsure if this finding will persist in larger sample sizes.

Time was a barrier to accomplishing delivery of all components of the curriculum, and we hope to refine or remove less important components. For example, all residents (pre- and postmodule) and faculty reported that good doctors should be honest about errors they make, suggesting this belief may be a less important target for intervention. The time spent on discussion of values and underlying medical ethics could be used to give more time for other components. The literature has shown a disconnect between belief in the appropriateness of disclosure, intent to disclose, and actual disclosure rates. Therefore, resident and patient-oriented outcomes are an important next step in curriculum assessment (e.g., resident milestones data, error reporting rates at primary rotation sites, and institutional patient safety survey information).

Limitations of our evaluation stemmed from an ambitious desire to develop a comprehensive curriculum, limited sample size and time, lack of direct skills and knowledge assessments, and missing team and patient-oriented outcomes. As such, our curriculum and evaluation do not allow for specific module content to be associated with changes in resident responses. Similarly, the complexity of error response and the scope and time for our curriculum evaluation limited our ability to associate our curriculum with specific changes in rates of error acknowledgement, disclosure, coping, and growth, or patient-oriented outcomes like satisfaction with error disclosure or care relationship after error. Our small sample size limits generalizability, and we hope to offer a refined curriculum to our entire graduate medical education community, which will help with sample size limitations.

Our timeframe allowed only for short-term reassessment of survey responses, which could differ from long-term impacts. Future studies could use resident milestone data to assess measurable outcomes, and a 6-month postsurvey could be undertaken to see if changes in knowledge, skills, and beliefs persist. Organizational data like error reporting rates and patient safety survey data could be tracked by year to evaluate potential impacts organization-wide. The project also uncovered a need for faculty development in medical error response, and sessions may be adapted for this purpose.

Our brief curriculum was associated with an increase in related resident reported knowledge, confidence, and story sharing. The medical community will benefit from further refining the model for error management and growth behaviors among residents. It will also be important to move from short-term, self-reported resident knowledge and beliefs to long-term resident and patient-oriented outcomes like error reporting rate, disclosure skills assessment by faculty and patient, and overall error rates. We intend to improve our postmodule assessment by adding direct skills assessment for error disclosure and incorporating resident and patient-oriented outcomes like reporting rates and patient safety survey data. Increasing our sample size by offering the curriculum to our local graduate medical education community including numerous specialty programs will allow us to better compare pre- and postmodule results. Ultimately, development of interdisciplinary and interprofessional error response training will best prepare learners to manage their future errors and their personal recovery.

## Appendices


Facilitators Guide.docxError Session 1.pptxError Session 1 Handout.pdfError Session 2.pptxError Session 3.pptxError Session 3 Handout - Error Cases.docxFaculty Survey.docxPremodule Resident Survey.docxPostmodule Resident Survey.docx
*All appendices are peer reviewed as integral parts of the Original Publication.*
