# Correction: Medical Error: Using Storytelling and Reflection to Impact Resident Error Response Factors

**DOI:** 10.15766/mep_2374-8265.11539

**Published:** 2025-05-29

## Correction

In the publication, “Medical Error: Using Storytelling and Reflection to Impact Resident Error Response Factors,” the Figure 3 y-axis middle bar and lower bar were incorrectly labeled. The middle bar parentheses should read (Resident Post), and the lower bar parentheses should read (Resident Pre). The correct Figure 3 is:

**Figure 3.**
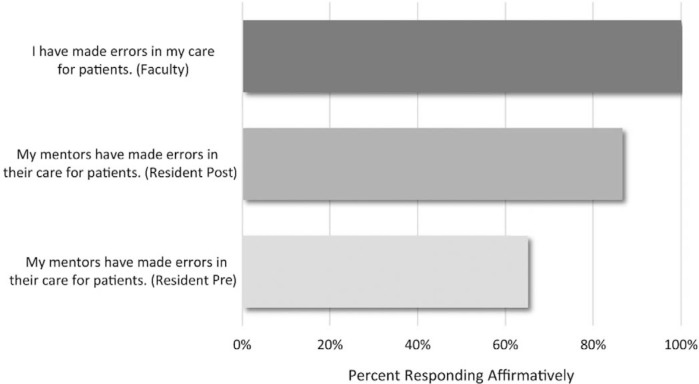
Resident premodule (*N* = 22) and postmodule (*N* = 15) perception of mentor error and faculty (*N* = 7) reporting of error, both presented as percent responding affirmatively (*Yes*).
