# Supplementary material for: Medical Error: Using Storytelling and Reflection to Impact Resident Error Response Factors
Source: MedEdPORTAL. 2024 Oct 10;20:11451. doi: 10.15766/mep_2374-8265.11451 (PMC11466310; doi:10.15766/mep_2374-8265.11451)
Supplement: Supplementary file 1 — Facilitators Guide.docxError Session 1.pptxError Session 1 Handout.pdfError Session 2.pptxError Session 3.pptxError Session 3 Handout - Error Cases.docxFaculty Survey.docxPremodule Resident Survey.docxPostmodule Resident Survey.docx [file mep_2374-8265.11451-s001.zip › A. Facilitators Guide.docx]

This guide offers framing and suggestions for those implementing medical error response curriculum.

**Background**

Our curriculum is designed to fill a need in graduate medical education for training in medical error acknowledgement and recovery. It was designed for initial use in a Family Medicine residency but is easily adapted to other specialties and could be adapted to undergraduate medical education or faculty development in this area.

1. Error is common to physicians and impacts us and our patients
2. Maladaptive coping is common
3. Our local culture around reporting impacts physician behavior
4. There are evidence-based steps to patient-centered error disclosure
5. There are evidence-based steps to physician growth after error
6. Talking is critical
7. Our stories can be career and life changing for learners

*Overall goal*:

The curriculum centers on the development of a psychologically safe space away from daily responsibilities so that learners can hear the very personal stories and experiences of more tenured physicians, have the opportunity to reflect, and work independently and together to practice related skills.

*Related AAMC Quality Improvement and Patient Safety Competencies*^1^

Domain I, Patient Safety

#2 Self and team care

#4 Identify and report safety events and hazards

#5 Disclose patient safety events

#6 Report, manage, and analyze patient safety events

#7 Describe common types of error and work to decrease risk

#8 Understand the role of culture in patient safety and contribute to just culture

Domain IV: Patients and Families as QIPS Partners

#4 Disclose patient safety events

*Related ACGME Milestones (Family Medicine)*^2^

1. Systems-Based Practice 1: Patient Safety and Quality Improvement
2. Professionalism 1: Professional Behavior and Ethical Principles
3. Professionalism 3: Self-Awareness and Help-Seeking Behaviors
4. Interpersonal and Communication Skills 1: Patient- and Family-Centered Communication
5. Interpersonal and Communication Skills 2: Interprofessional and Team Communication
6. Interpersonal and Communication Skills 3: Communication within Health Care Systems

*Model for curriculum development*:

Using the PRECEDE/PROCEED model,^3^ a logic model for developing health interventions, we organized key factors related to effective error management and physician growth after error.^4^ PRECEDE stands for Predisposing, Reinforcing, and Enabling Constructs in Educational/Environmental Diagnosis and Evaluation; and PROCEED spells out Policy, Regulatory, and Organizational Constructs in Educational and Environmental Development.

Factors identified through literature review related to effective physician error management and growth after error (bold items were selected as targets). Adapted from Langlois and Hallam.

| **Predisposing** | **Enabling** | **Reinforcing** |
| --- | --- | --- |
| Training^5–8^ | **Skill - Identify error and causes**^9^ | Reminders – routine error processing/daily activities^10^ |
| Know what error is^11^ | **Skill – disclose error**^6,7,9,12–18^ | Reminders – routine peer and mentor support^9^ |
| **Know steps to effective disclosure**^9^ | **Skill – manage emotions**^9,19,20^ | Positive reinforcement – quality of disclosure^21,22^ |
| Know factors associated with physician recovery^19,23,24^ | **Skill - cope**^5,13,20,25–27^ | **Positive reinforcement – ‘talking’ to process**^9,19,28^ |
| **Know related professional values**^29,30^ | **Skill – access support**^9^ | Positive reinforcement – feedback regarding process improvement after error reporting^9^ |
| **Know local policies and procedures**^5,29^ | Access – support for reporting, disclosing from peers and supervisors^10^ | Support – family, friends, colleagues, mentors, mental health, supervisor, patient safety organizations, religious community^19,20,31^ |
| **Believe error is a common experience**^9^ | Access – nonpunitive environment^18,29^ |  |
| **Believe doctors should disclose**^32^ | Access – role models^5^ |  |
| Believe it is safe and effective to disclose^31^ | Access – routine error debriefing/daily activities^10^ |  |
| **Believe I can recover/grow after error**^8^ | Access – easy reporting^29,33,34^ |  |
| Intend to grow from errors^9^ | Access – mental health support^25^ |  |
|  | Access – colleague and mentor support for recovery and growth^19,20^ |  |

*Session Objectives and Teaching Methods:*

| Session | Objectives | Methods |
| --- | --- | --- |
| Session one  (personal stories)  Appendix B Slides | Understand that error is a common experience to all physicians.  Define error and describe common causes of error.  Identify ways that physicians cope and thrive after error.  Develop a strategy for acknowledging and processing medical error. | Mentor storytelling  Guided self reflection (see medical error story session 1 handout)  Lecture  Large group discussion |
| Session two  (ethics and culture)  Appendix C Slides | Apply professional values to the topic of medical error.  Openly discuss fears related to medical error.  Describe safety culture and identify ways that colleagues can help with error management and recovery | Lecture  Small and large group discussion  Self-reflection (writing)  Optional: Polling software |
| Session three  (local policies and practice)  Appendix D Slides | Describe local policies and practices related to medical error.  Practice self-awareness, open disclosure, and root cause reflection (abbreviated root cause analysis).  Practice coping skills. | Small and large group discussion  Role play (see clinical cases handout)  Self-reflection (writing, letter to future self)  Optional: Polling software |

**Tips on implementation**

It is important to consider the timing of sessions in terms of the trainee's education and clinical experience. It may be helpful to allow the resident to build foundational experience on the floors participating in the daily care of patients before offering this exercise. Direct clinical experience provides an underpinning and frame of reference for them as they engage in this activity. It also provides the opportunity to observe more tenured peers and faculty engage in the activity of disclosure, as well as the equally important task of preparing for the disclosure itself. Therefore, too early in their intern year may be less than ideal. Much past the beginning of their second year may be too late, as they will have undoubtedly faced this difficult task themselves by then. Having said that, the literature is inconclusive about the ideal timing.

*Proposed Timeline:*

| Task | Timing |
| --- | --- |
| Identify faculty curriculum champion | Start of academic year |
| Schedule sessions 1, 2, and 3 | Start of academic year |
| Disseminate pre-intervention and faculty survey tools | 3-4 months into the academic year |
| Review above survey tool data to identify strengths and opportunities of current curriculum | 4-5 months into the academic year |
| Personalize materials   1. Identify local faculty storytelller 2. Seek error scenarios for roleplay 3. Collect and add information regarding local policies and practices, local resources-pertinent hospital departments and champions, and mental health support for residents to slides | 4-5 months into academic year |
| Carry out Session 1 | 5-6 months into academic year |
| Carry out Session 2 | 6-7 months into academic year |
| Carry out Session 3 | 7-8 months into academic year |
| Disseminate post-intervention survey tool | Immediately or soon after completing Session 3 |
| Assess curriculum outcomes | 9-12 months into academic year |

*Checklist/Preparation for session 1:*

1. Time protected from clinical duties, the first of three 60-minute sessions, over the course of 3-6 months.
2. Quiet space
3. Microphone if large area so that residents can be heard during large group discussion.
4. A story. Local faculty/mentor highly preferred.
   1. Instructions for storytellers: We believe that the most impactful stories will come from program mentors and leadership. Generally, we want to reiterate that the discussion is confidential and in the spirit of learning.  The purpose of the story-sharing is to reflect on ways that physicians react to medical error, amplifying evidence-based strategies from the literature that result in good patient care and care for the involved physician(s).  Stories do not have to be long, 5-10 minutes works well.
   2. If a local story is unavailable, consider written stories from the literature or searching for videos of physicians sharing stories through Harvard (Healing the Healer)^35^ or University of Virginia School of Medicine (Wisdom Through Adversity)^36^, see article references for numerous sources.
5. “Medical Error Session 1 Handout” and pens.
6. Blank paper and pens for learners to take note of their own coping skills if they choose.
7. OPTIONAL: Consider inserting images of faculty from YOUR program who are champions, or researchers/activists that you admire (Appendix B, Slide 15).
8. OPTIONAL: There are two videos available after the resources slide if you have the time to include or would like to provide learners with a link to view later.
9. OPTIONAL: There are prompts within the slide deck where faculty can incorporate polling software if they choose.
10. Facilitator familiar with slides
11. Humility and courage, get ready to do some powerful learning!

*Checklist/Preparation for session 2:*

1. Time protected from clinical duties, the second of three 60-minute sessions, over the course of 3-6 months.
2. Quiet space
3. Tables, chairs, and space that allow residents to partner up and get into groups of 3
4. Microphone if large area so that residents can be heard during discussion.
5. Blank paper and pen for self reflection exercises
6. OPTIONAL: There are prompts within the slide deck where faculty can incorporate polling software if they choose.
7. Facilitator familiar with slides
8. Humility and courage, get ready to do some powerful learning!

*Checklist/Preparation for session 3:*

1. Time protected from clinical duties, the third of three 60-minute sessions, over the course of 3-6 months.
2. Quiet space
3. Tables and chairs that allow residents to partner up and get into groups of 3
4. Microphone if large area so that residents can be heard during discussion.
5. Facilitator familiar with local policies (program sponsor, clinic, adult, and/or pediatric hospital) related to error. Knowing how policies are similar and different can help guide and inform learners (session 3).
6. “Clinical error cases for session 3 roleplay” handout OR Faculty can develop a few different error cases for session 3 (at least 3 different error stories), for small group practice. Error cases should be local, specific to specialty, de-identified and short (few sentences). They can be partially or completely fictionalized. Remember the goal is to focus on resident skills including disclosure and not on the medical facts of the case.
   1. If local cases are not available, consider cases from the Agency for Healthcare Research and Quality Patient Safety Network website, under Web M&M Case Studies.^37^ Of note, you can submit anonymous cases here as well.
7. Blank paper and pen for self reflection exercises
8. OPTIONAL: There is one video (slide 12) available if you have the time to include or would like to provide learners with a link to view later.
9. OPTIONAL: Feel free to personalize Appendix D, specifically Slide 4 (local policies), 7 (local reporting processes), 8 (local pertinent champions/site contacts), and 19 (local and/or recommended support resources)
10. OPTIONAL: There are prompts within the slide deck where faculty can incorporate polling software if they choose.
11. Facilitator familiar with slides
12. Humility and courage, get ready to do some powerful learning!

*Optional Resources that may enhance learning:*

1. Slides were prepared to be tailored for each educational setting. Specifically, programs are encouraged to insert pictures of local faculty champions and mentors and information on local site policies and practices related to error.
2. Consider the use of polling software to increase engagement, but as always, technology can fail, and it is important not to slow the momentum of group discovery if technical challenges arise.
3. Several online video resources proved valuable in our course development. These optional resources can be shared during sessions, or links provided for further independent study.
   1. The TED Talk “Doctors Make Mistakes. Can We Talk About That?” by a gifted speaker, Dr. Brian Goldman^38^
   2. “Healing the Healer Film”^35^ (Harvard.edu) discusses error as a common experience for physicians and demonstrates that patient-physician relationships can survive/thrive even after an error
   3. “Medical Error: A Case Based Approach to Apology and Disclosure Video^39^” developed by Brigham and Women’s Hospital and narrated by Dr. Jo Shapiro, and finally
   4. “Choosing Wisdom: The Path Through Adversity,”^36^ a documentary exploring the themes medical error and recovery based on research from University of Virginia.
4. Facilitator familiar with any polling software and any included videos
5. Feedback and support during small group work and facilitation of large group discussion may be enriched by faculty who work in patient safety and risk management as well as psychology.
6. A powerful potential addition to the faculty team could include a patient or family member who has been on the receiving end of a disclosure conversation. The patient portends the best teacher in offering feedback about how well or not, a disclosure of an adverse event was executed. While this is not required and may create logistical barriers, it can be very effective. If this is not possible, the roleplay scenarios do include the opportunity to play the role of the patient / family.

**Options for curricular assessment:**

1. Use resident survey tools, as we did in our pilot study. Build time into session for survey completion to increase response rates.
2. Other methods that could be used:
   1. Qualitative feedback from faculty and learners
   2. Direct knowledge / skills assessment of learners
   3. Organizational patient safety indicators (error reporting rates)
   4. Learner burnout rates
   5. Patient or simulated patient feedback after error disclosure

References

1. AAMC. *Quality Improvement and Patient Safety Competencies Across the Learning Continuum. AAMC New and Emerging Areas in Medicine Series.* .; 2019.

2. ACGME. *Family Medicine Milestones*.; 2019.

3. Langlois MA, Hallam JS. Integrating multiple health behavior theories into program planning: the PER Worksheet. *Health Promot Pract*. 2010;11(2):282-288. doi:10.1177/1524839908317668

4. Adkins S. Curriculum Background. Retrieved from The STFM Resource Library: https://resourcelibrary.stfm.org/viewdocument/medical-error-disclosure-and-manage?CommunityKey=2751b51d-483f-45e2-81de-4faced0a290a&tab=librarydocuments.

5. Fischer MA, Mazor KM, Baril J, Alper E, DeMarco D, Pugnaire M. Learning from mistakes. Factors that influence how students and residents learn from medical errors. *J Gen Intern Med*. 2006;21(5):419-423. doi:10.1111/j.1525-1497.2006.00420.x

6. Aaron M, Webb A, Luhanga U. A Narrative Review of Strategies to Increase Patient Safety Event Reporting by Residents. *J Grad Med Educ*. 2020;12(4):415-424. http://ezproxy.libraries.wright.edu/login?url=https://search.ebscohost.com/login.aspx?direct=true&db=edb&AN=145172652&site=eds-live

7. Sukalich S, Elliott JO, Ruffner G. Teaching medical error disclosure to residents using patient-centered simulation training. *Academic Medicine*. 2014;89(1):136-143. doi:10.1097/ACM.0000000000000046

8. White AA, Bell SK, Krauss MJ, et al. How trainees would disclose medical errors: educational implications for training programmes. *Med Educ*. 2011;45(4):372-380. doi:10.1111/j.1365-2923.2010.03875.x

9. Plews-Ogan M, May N, Owens J, Ardelt M, Shapiro J, Bell SK. Wisdom in Medicine: What Helps Physicians After a Medical Error? *ACADEMIC MEDICINE*. 2016;91(2):233-241. doi:10.1097/ACM.0000000000000886

10. Fox MD, Bump GM, Butler GA, Chen LW, Buchert AR. Making Residents Part of the Safety Culture: Improving Error Reporting and Reducing Harms. *J Patient Saf*. 2021;17(5):e373-e378. doi:10.1097/PTS.0000000000000344

11. Penson RT, Svendsen SS, Chabner BA, Lynch TJ, Levinson W. Medical mistakes: a workshop on personal perspectives. *Oncologist*. 2001;6(1):92-99. doi:10.1634/theoncologist.6-1-92

12. Bonnema RA, Gonzaga AMR, Bost JE, Spagnoletti CL. Teaching error disclosure: Advanced communication skills training for residents. *J Commun Healthc*. 2012;5(1):51-55. http://ezproxy.libraries.wright.edu/login?url=https://search.ebscohost.com/login.aspx?direct=true&db=ufh&AN=74491295&site=eds-live

13. Asakawa M, Imafuku R, Kawakami C, Hayakawa K, Suzuki Y, Saiki T. Promoting a culture of sharing the error: A qualitative study in resident physicians’ process of coping and learning through self-disclosure after medical error. *Front Med (Lausanne)*. 2022;9. doi:10.3389/fmed.2022.960418

14. Kaldjian LC, Jones Elizabeth W. Gary E. R. Facilitating and Impeding Factors for Physicians’ Error Disclosure: A Structured Literature Review. *The Joint Commission Journal on Quality and Patient safety*. 2006;32(4):188-198. http://ezproxy.libraries.wright.edu/login?url=https://search.ebscohost.com/login.aspx?direct=true&db=edselp&AN=S1553725006320247&site=eds-live

15. Hannawa AF, Shigemoto Y, Little TD. Medical errors: Disclosure styles, interpersonal forgiveness, and outcomes. *Soc Sci Med*. 2016;156:29-38. http://ezproxy.libraries.wright.edu/login?url=https://search.ebscohost.com/login.aspx?direct=true&db=edselp&AN=S0277953616301265&site=eds-live

16. Gallagher TH, Waterman AD, Ebers AG, et al. Patients’ and physicians’ attitudes regarding the disclosure of medical errors. *JAMA: Journal of the American Medical Association*. 2003;289(8):1001-1007. doi:10.1001/jama.289.8.1001

17. Helmchen LA, Richards MR, McDonald TB, Helmchen LA, Richards MR, McDonald TB. How does routine disclosure of medical error affect patients’ propensity to sue and their assessment of provider quality? Evidence from survey data. *Med Care*. 2010;48(11):955-961. doi:10.1097/MLR.0b013e3181eaf84d

18. Cox LM, Logio LS. Patient safety stories: a project utilizing narratives in resident training. *Acad Med*. 2011;86(11):1473-1478. doi:10.1097/ACM.0b013e318230efaa

19. Harrison R, Johnson J, McMullan RD, et al. Toward Constructive Change After Making a Medical Error: Recovery From Situations of Error Theory as a Psychosocial Model for Clinician Recovery. *J Patient Saf*. 2022;18(6):587-604. http://ezproxy.libraries.wright.edu/login?url=https://search.ebscohost.com/login.aspx?direct=true&db=edo&AN=158599120&site=eds-live

20. Engel KG, Rosenthal M, Sutcliffe KM. Residents’ Responses to Medical Error: Coping, Learning, and Change. *Academic Medicine*. 2006;81(1):86-93. doi:10.1097/00001888-200601000-00021

21. Waterman Hazel Dunagan Amy D. Jane Erik William Claiborne G, Levinson W, Fraser VJ, Gallagher TH. The Emotional Impact of Medical Errors on Practicing Physicians in the United States and Canada. *The Joint Commission Journal on Quality and Patient safety*. 2007;33(8):467-476. http://ezproxy.libraries.wright.edu/login?url=https://search.ebscohost.com/login.aspx?direct=true&db=edselp&AN=S155372500733050X&site=eds-live

22. Mccay L, Wu AW. Medical error: the second victim. *British Journal of Hospital Medicine (17508460)*. 2012;73(10):C146-C148. doi:10.12968/hmed.2012.73.Sup10.C146

23. Scott SD, Hirschinger LE, Cox KR, McCoig M, Brandt J, Hall LW. The natural history of recovery for the healthcare provider “second victim” after adverse patient events. *Qual Saf Health Care*. 2009;18(5):325-330. doi:10.1136/qshc.2009.032870

24. Laurent A, Aubert L, Chahraoui K, et al. Error in intensive care: psychological repercussions and defense mechanisms among health professionals. *Crit Care Med*. 2014;42(11):2370-2378. doi:10.1097/CCM.0000000000000508

25. Fatima S, Soria S, Esteban- Cruciani N. Medical errors during training: how do residents cope?: a descriptive study. *BMC Med Educ*. 2021;21(1):1-6. http://ezproxy.libraries.wright.edu/login?url=https://search.ebscohost.com/login.aspx?direct=true&db=edb&AN=151647186&site=eds-live

26. Goldberg RM, Kuhn G, Andrew LB, et al. Coping with medical mistakes and errors in judgment. *Ann Emerg Med*. 2002;39(3):287-292. http://ezproxy.libraries.wright.edu/login?url=https://search.ebscohost.com/login.aspx?direct=true&db=rzh&AN=106944186&site=eds-live

27. Plews-Ogan M, Owens JE, May NB. Wisdom through adversity: Learning and growing in the wake of an error. *Patient Educ Couns*. 2013;91(2):236-242. http://ezproxy.libraries.wright.edu/login?url=https://search.ebscohost.com/login.aspx?direct=true&db=edselp&AN=S0738399112005198&site=eds-live

28. May N, Plews-Ogan M. The role of talking (and keeping silent) in physician coping with medical error: A qualitative study. *Patient Educ Couns*. 2012;88(3):449-454. http://ezproxy.libraries.wright.edu/login?url=https://search.ebscohost.com/login.aspx?direct=true&db=edselp&AN=S0738399112002546&site=eds-live

29. Rashed A, Hamdan M. Physicians’ and Nurses’ Perceptions of and Attitudes Toward Incident Reporting in Palestinian Hospitals. *J Patient Saf*. 2019;15(3):212-217. doi:10.1097/PTS.0000000000000218

30. Kaldjian LC, Jones EW, Rosenthal GE, Tripp-Reimer T, Hillis SL. An empirically derived taxonomy of factors affecting physicians’ willingness to disclose medical errors. *J Gen Intern Med*. 2006;21(9):942-948. doi:10.1111/j.1525-1497.2006.00489.x

31. Harrison R, Lawton R, Perlo J, Gardner P, Armitage G, Shapiro J. Emotion and Coping in the Aftermath of Medical Error: A Cross-Country Exploration. *J Patient Saf*. 2015;11(1):28-35. doi:10.1097/PTS.0b013e3182979b6f

32. Kaldjian LC, Jones EW, Wu BJ, Forman-Hoffman VL, Levi BH, Rosenthal GE. Disclosing medical errors to patients: Attitudes and practices of physicians and trainees. *J Gen Intern Med*. 2007;22(7):988-996. doi:10.1007/s11606-007-0227-z

33. Perez B, Knych SA, Weaver SJ, et al. Understanding the Barriers to Physician Error Reporting and Disclosure: A Systemic Approach to a Systemic Problem. *J Patient Saf*. 2014;10(1):45-51. doi:10.1097/PTS.0b013e31829e4b68

34. Coyle YM, Mercer SQ, Murphy-Cullen CL, Schneider GW, Hynan LS. Effectiveness of a graduate medical education program for improving medical event reporting attitude and behavior. *Qual Saf Health Care*. 2005;14(5):383-388. doi:10.1136/qshc.2005.013979

35. Augello T. *Healing the Healer*.; 2010.

36. Plews-Ogan M, Owens J, Ardelt M, Calhoun L. *Choosing Wisdom: The Path Through Adversity*.; 2012.

37. Agency for Healthcare Research and Quality PSN. WebM&M: Case Studies.

38. Goldman B. Doctors Make Mistakes: Can We Talk about That. *TED talk*. Published online 2010. Video by TEDx, retrieved from https://www.ted.com/talks/brian_goldman_doctors_make_mistakes_can_we_talk_about_that?language=en is available under Creative Commons license Attribution - NonCommercial - No Derivatives (CC BY -- NC -- ND 4.0 International) https://creativecommons.org/licenses/by-nc-nd/4.0/deed.en. Accessed June 4, 2024.

39. Shapiro J. *Medical Error: A Case Based Approach to Apology and Disclosure Video*.; 2014.
