# Supplementary material for: Medical Error: Using Storytelling and Reflection to Impact Resident Error Response Factors
Source: MedEdPORTAL. 2024 Oct 10;20:11451. doi: 10.15766/mep_2374-8265.11451 (PMC11466310; doi:10.15766/mep_2374-8265.11451)
Supplement: Supplementary file 1 — Facilitators Guide.docxError Session 1.pptxError Session 1 Handout.pdfError Session 2.pptxError Session 3.pptxError Session 3 Handout - Error Cases.docxFaculty Survey.docxPremodule Resident Survey.docxPostmodule Resident Survey.docx [file mep_2374-8265.11451-s001.zip › C. Error Session 1 Handout.pdf]

Medical error session 1 – Notes page

<u>The Story</u>

1. Why did this happen?

2. What did the physician feel when faced with the error? What emotions do you feel?

3. What did the physician do when faced with the error? What would you do?

4. Did the physician recover? If so, how? If not, why not?

5. How did the patient discover and process the error?

6. Did anyone learn from or use the error for good? How?

7. What surprised you? Were any of your beliefs challenged?
