# Supplementary material for: Medical Error: Using Storytelling and Reflection to Impact Resident Error Response Factors
Source: MedEdPORTAL. 2024 Oct 10;20:11451. doi: 10.15766/mep_2374-8265.11451 (PMC11466310; doi:10.15766/mep_2374-8265.11451)
Supplement: Supplementary file 1 — Facilitators Guide.docxError Session 1.pptxError Session 1 Handout.pdfError Session 2.pptxError Session 3.pptxError Session 3 Handout - Error Cases.docxFaculty Survey.docxPremodule Resident Survey.docxPostmodule Resident Survey.docx [file mep_2374-8265.11451-s001.zip › D. Error Session 2.pptx]

## Slide 1
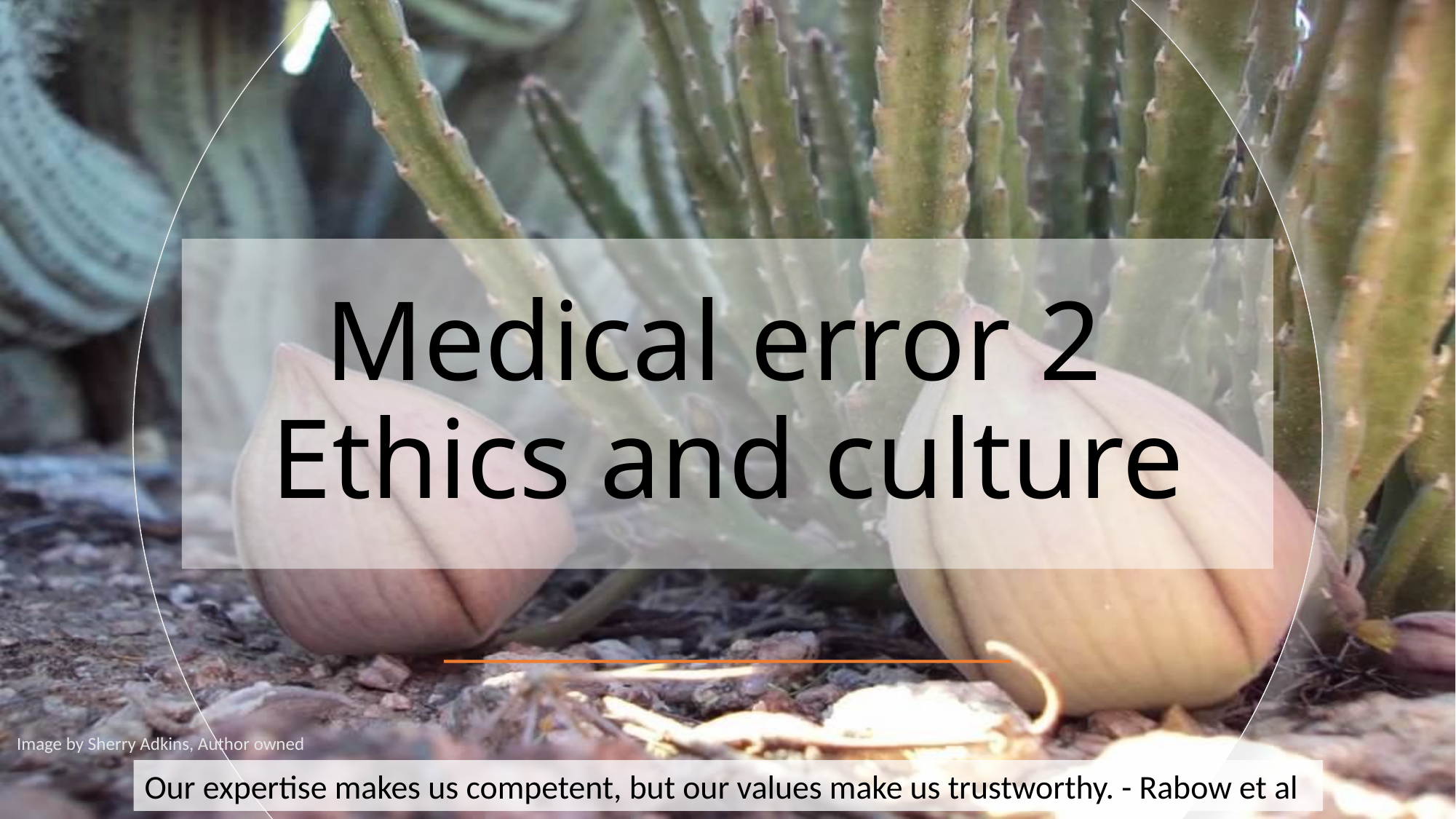

# Medical error 2  Ethics and culture
Image by Sherry Adkins, Author owned
Our expertise makes us competent, but our values make us trustworthy. - Rabow et al

## Slide 2
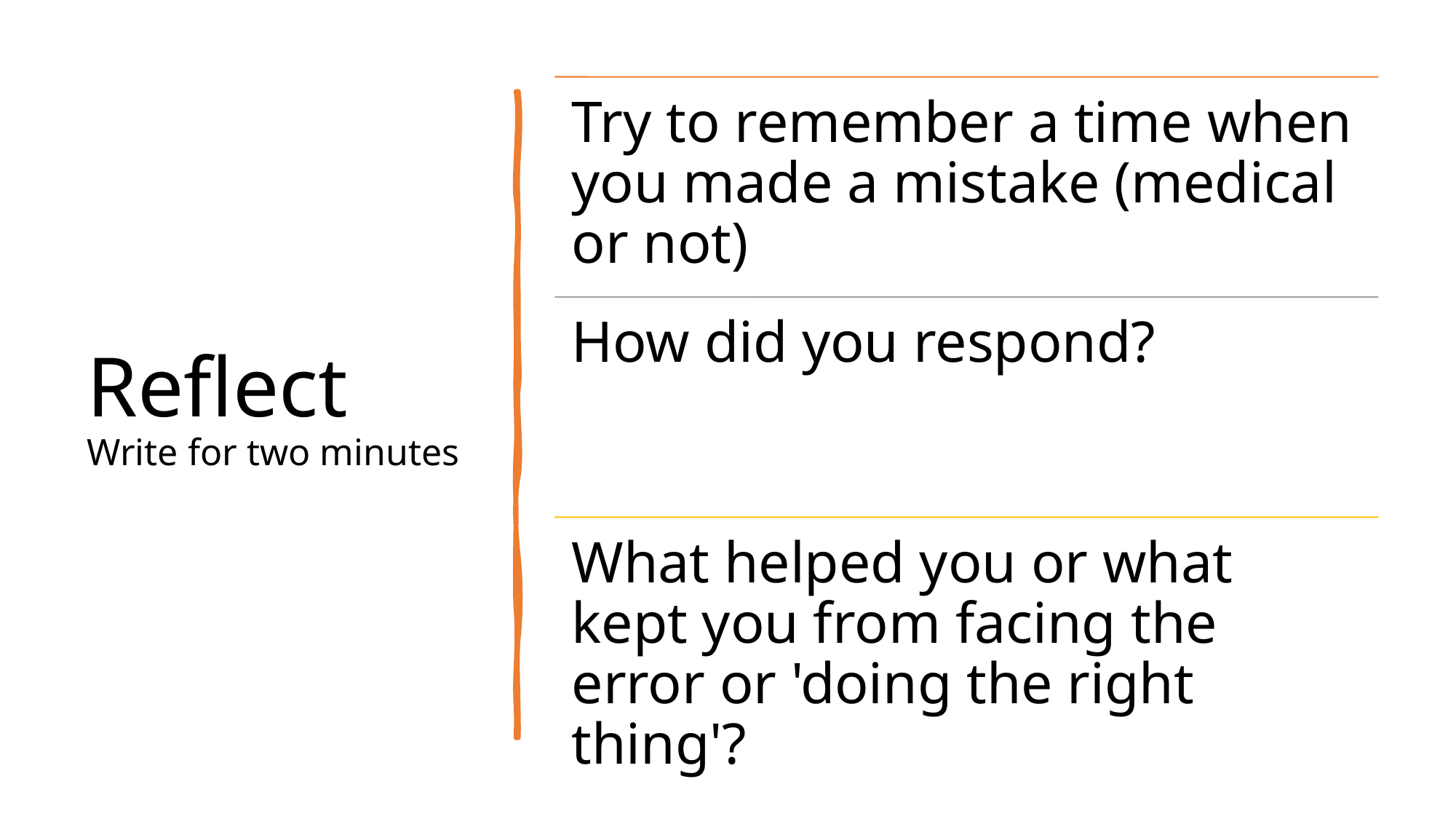

# Reflect Write for two minutes

## Slide 3
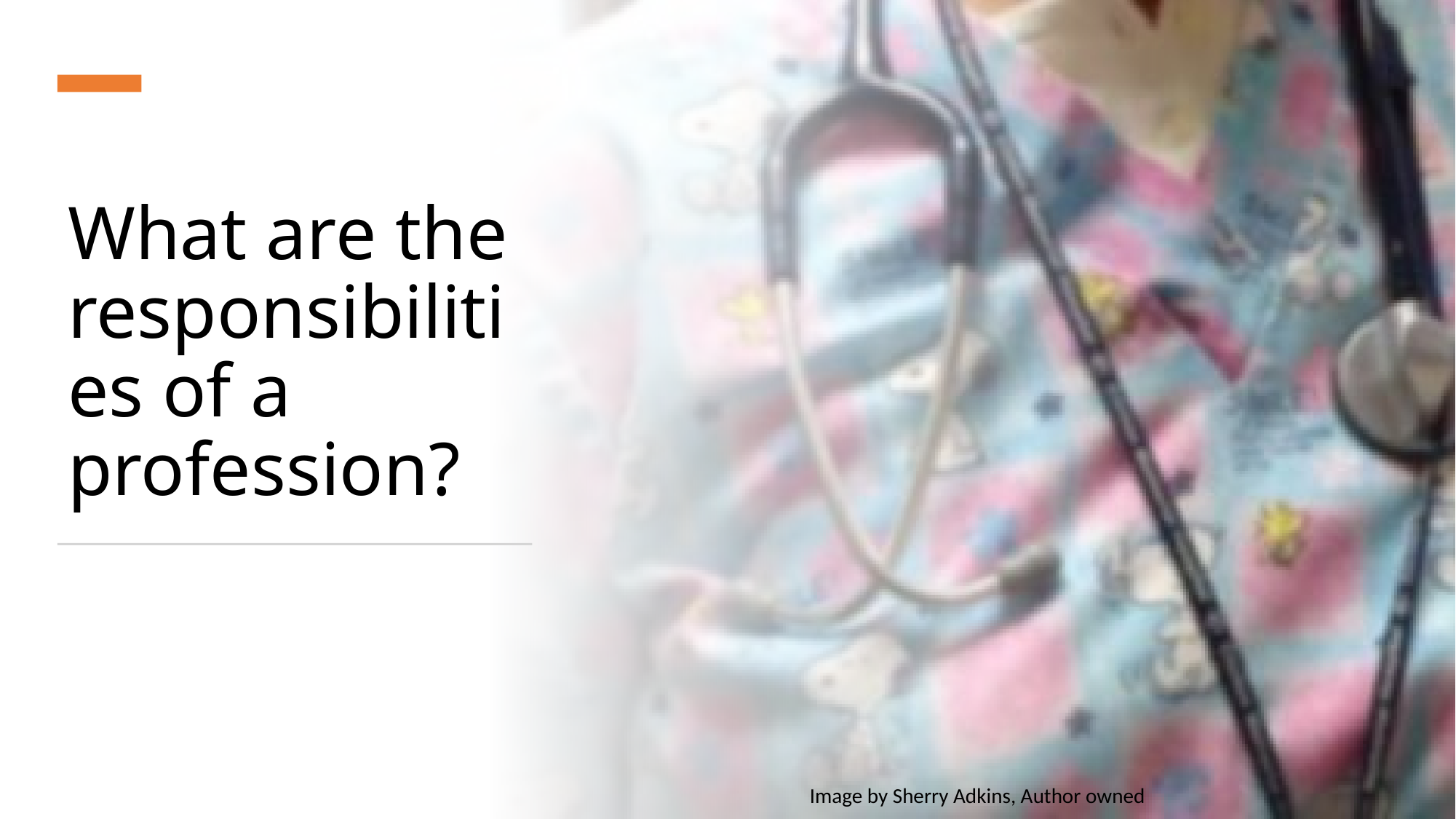

# What are the responsibilities of a profession?
Image by Sherry Adkins, Author owned

## Slide 4
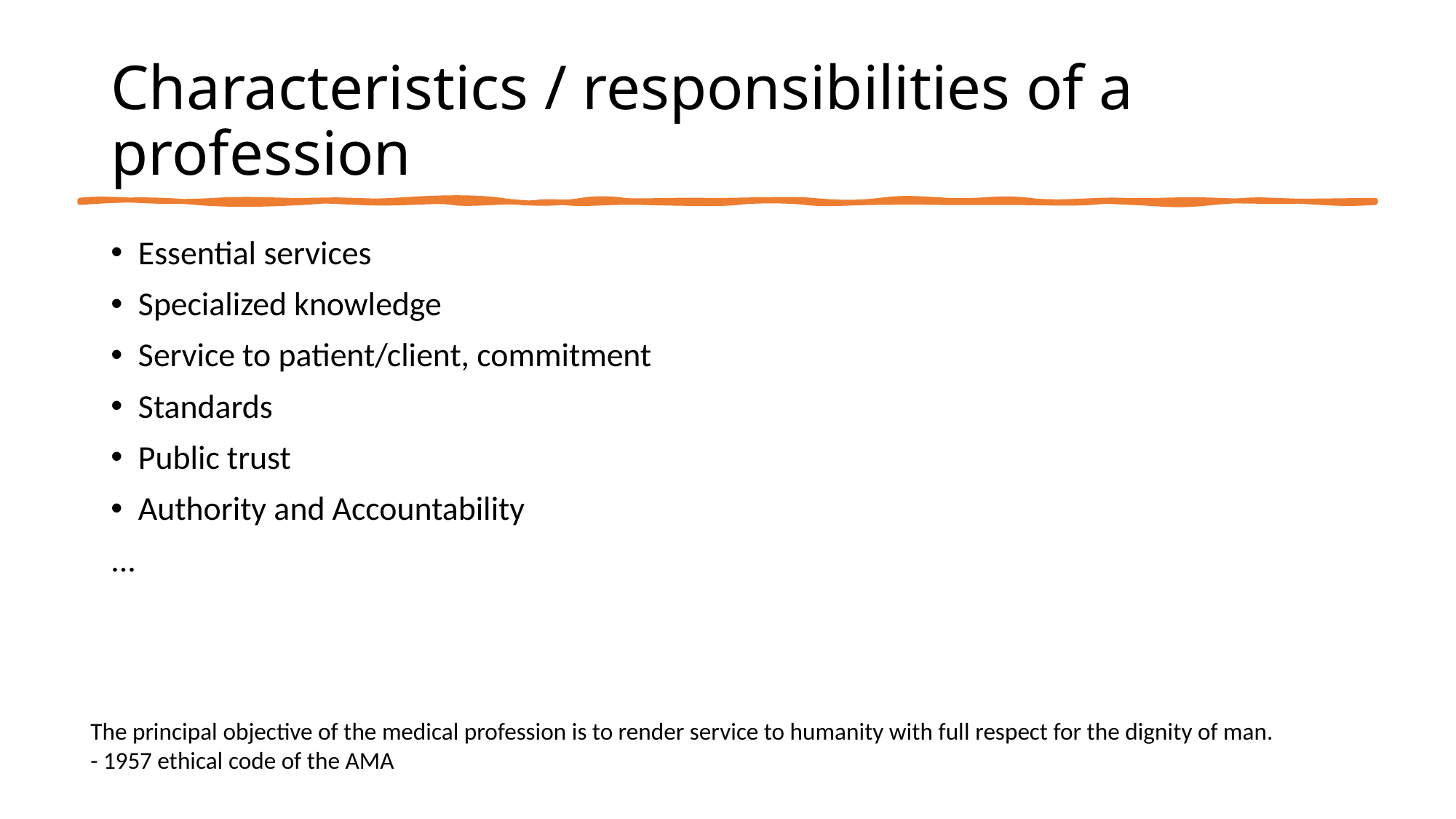

# Characteristics / responsibilities of a profession
Essential services
Specialized knowledge
Service to patient/client, commitment
Standards
Public trust
Authority and Accountability
...
The principal objective of the medical profession is to render service to humanity with full respect for the dignity of man.
- 1957 ethical code of the AMA

## Slide 5
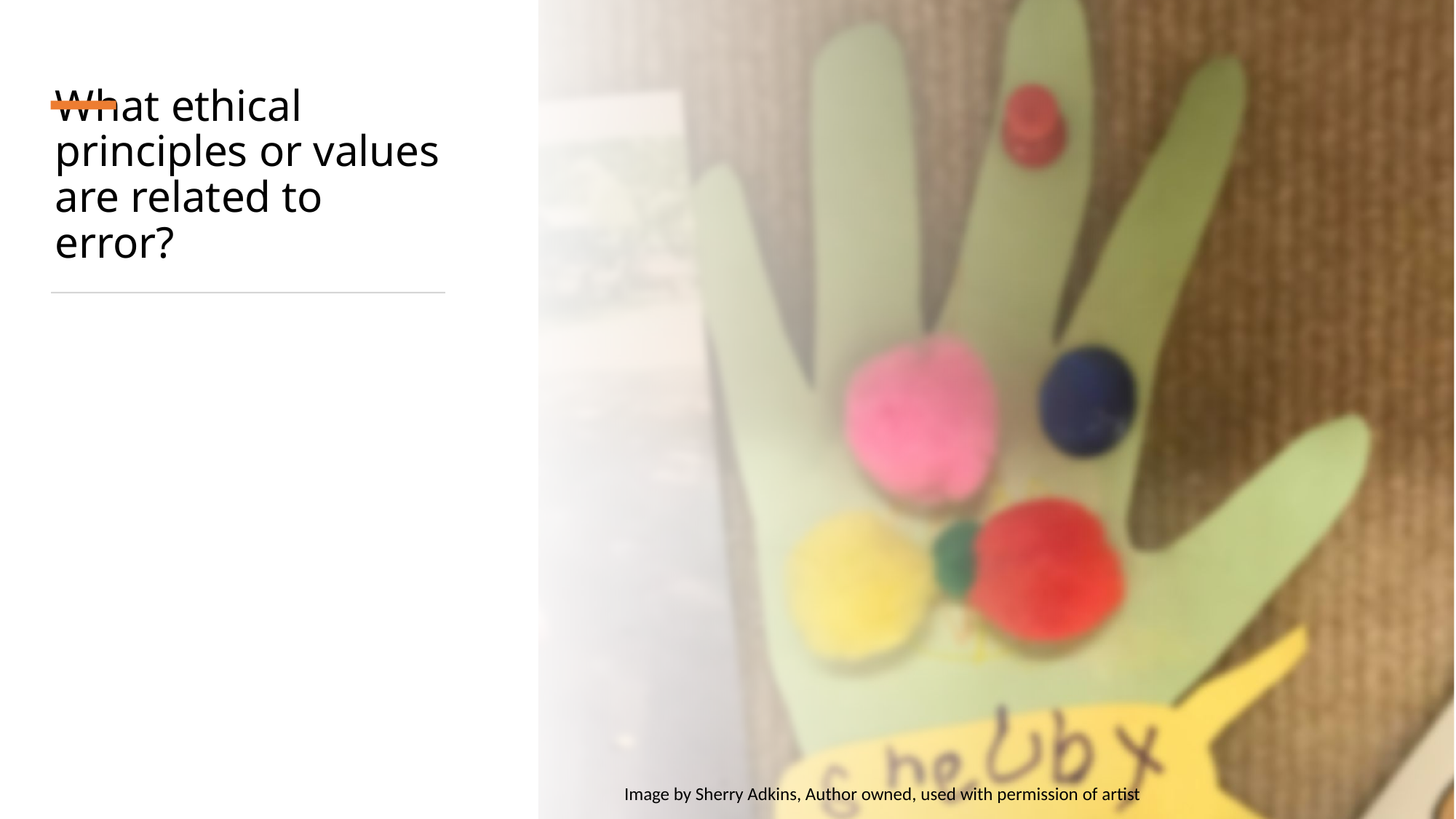

# What ethical principles or values are related to error?
Image by Sherry Adkins, Author owned, used with permission of artist

## Slide 6
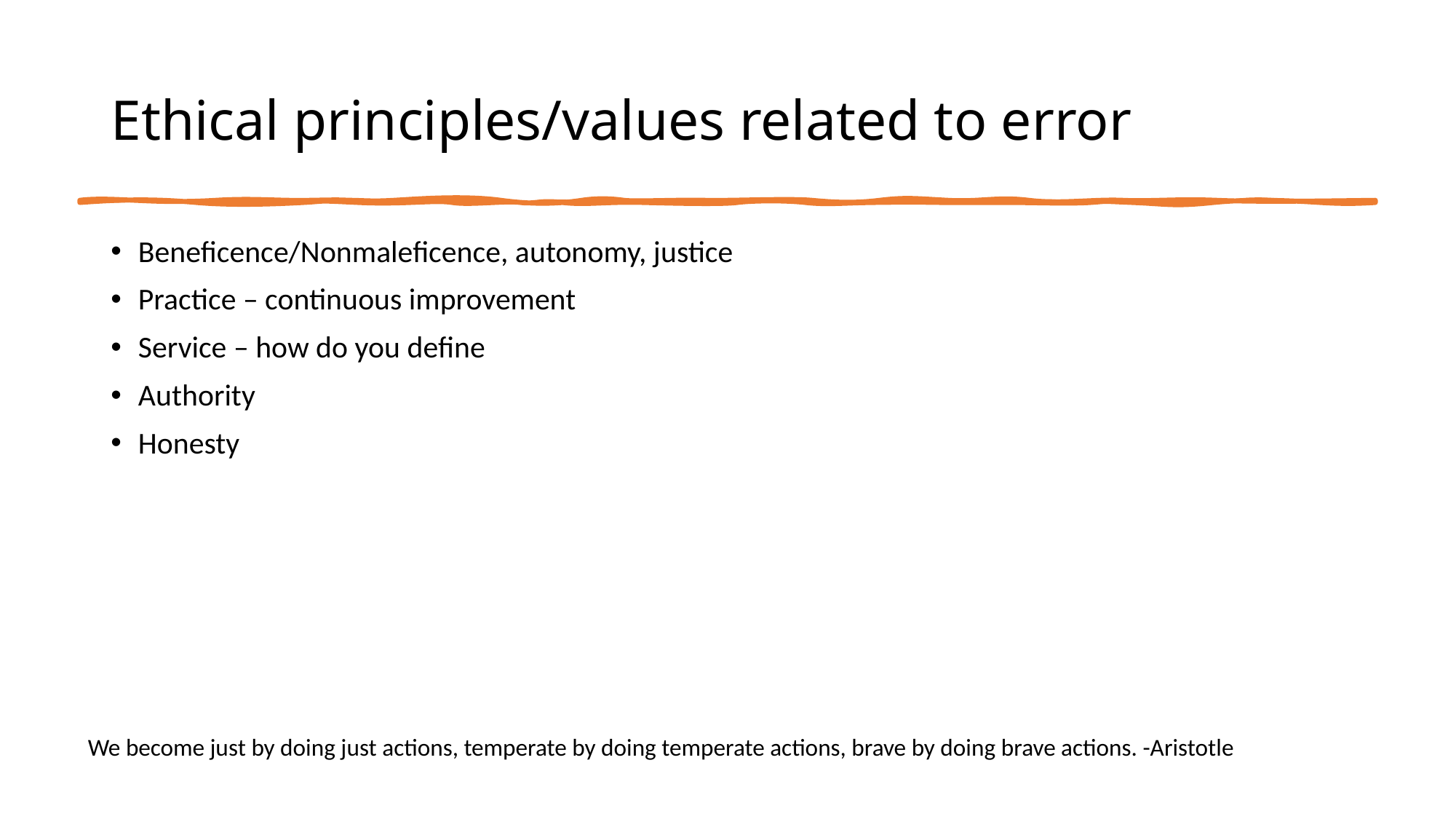

# Ethical principles/values related to error
Beneficence/Nonmaleficence, autonomy, justice
Practice – continuous improvement
Service – how do you define
Authority
Honesty
We become just by doing just actions, temperate by doing temperate actions, brave by doing brave actions. -Aristotle

## Slide 7
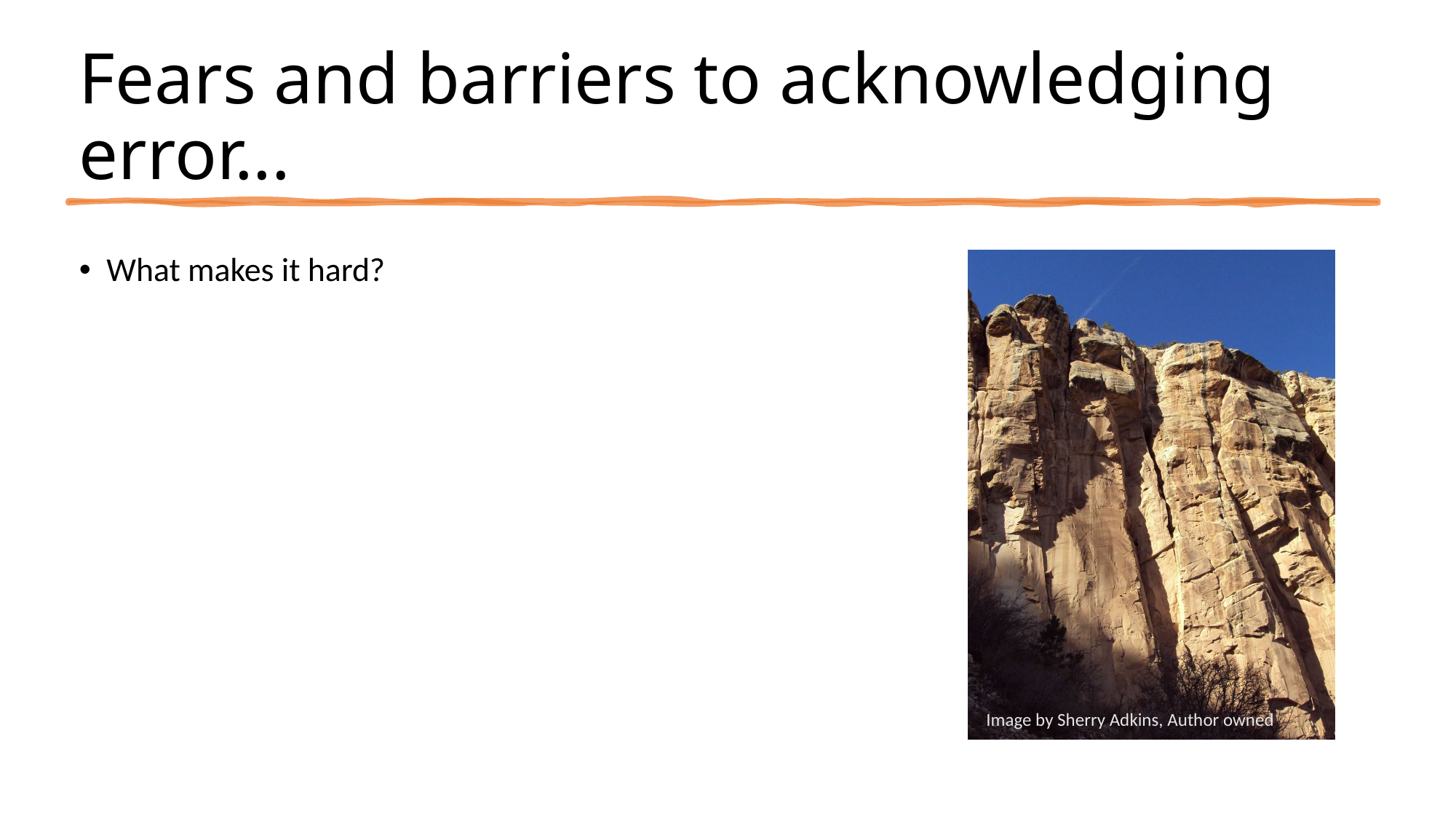

# Fears and barriers to acknowledging error...
What makes it hard?
Image by Sherry Adkins, Author owned

## Slide 8
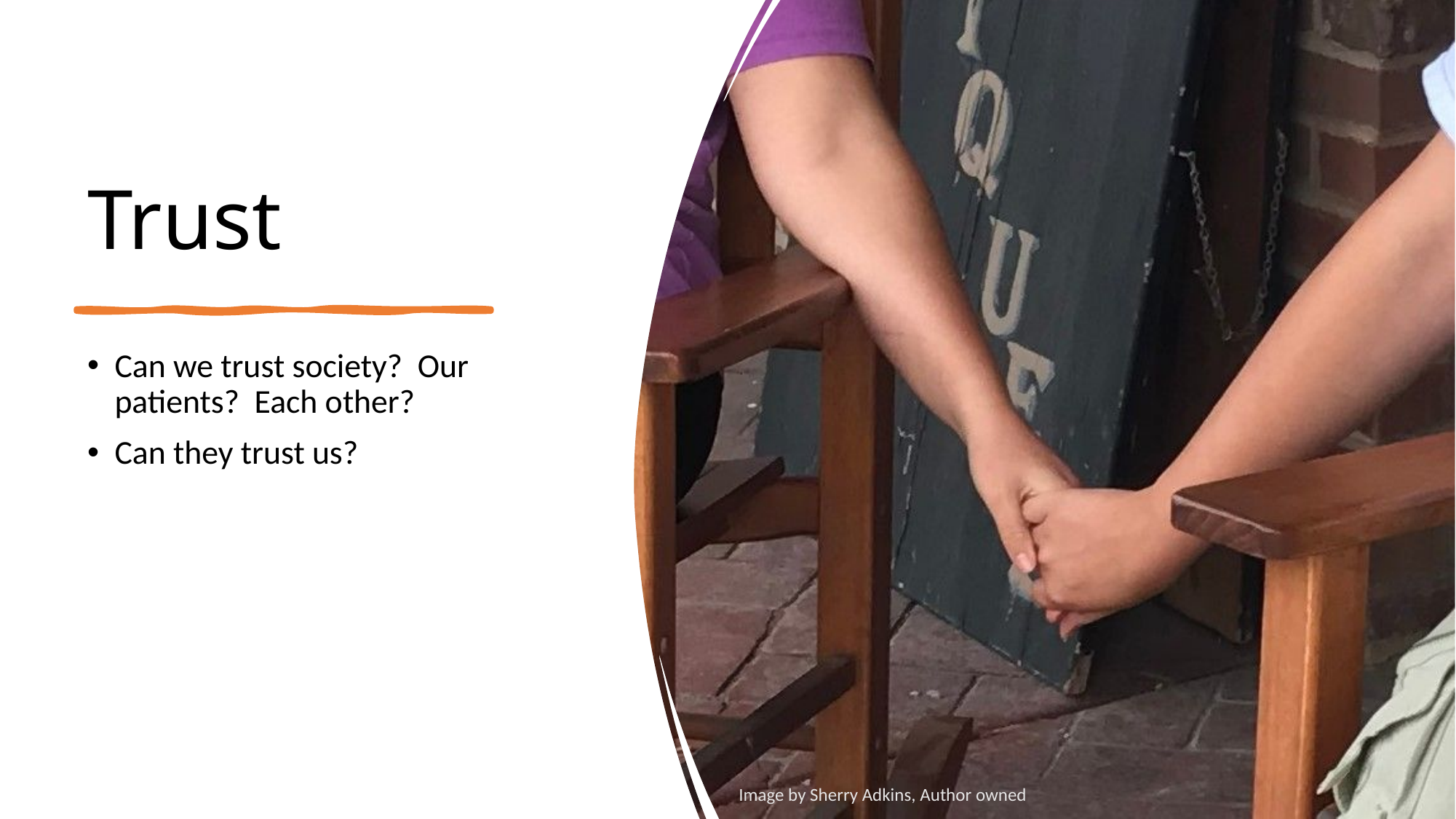

# Trust
Can we trust society?  Our patients?  Each other?
Can they trust us?
Image by Sherry Adkins, Author owned

## Slide 9
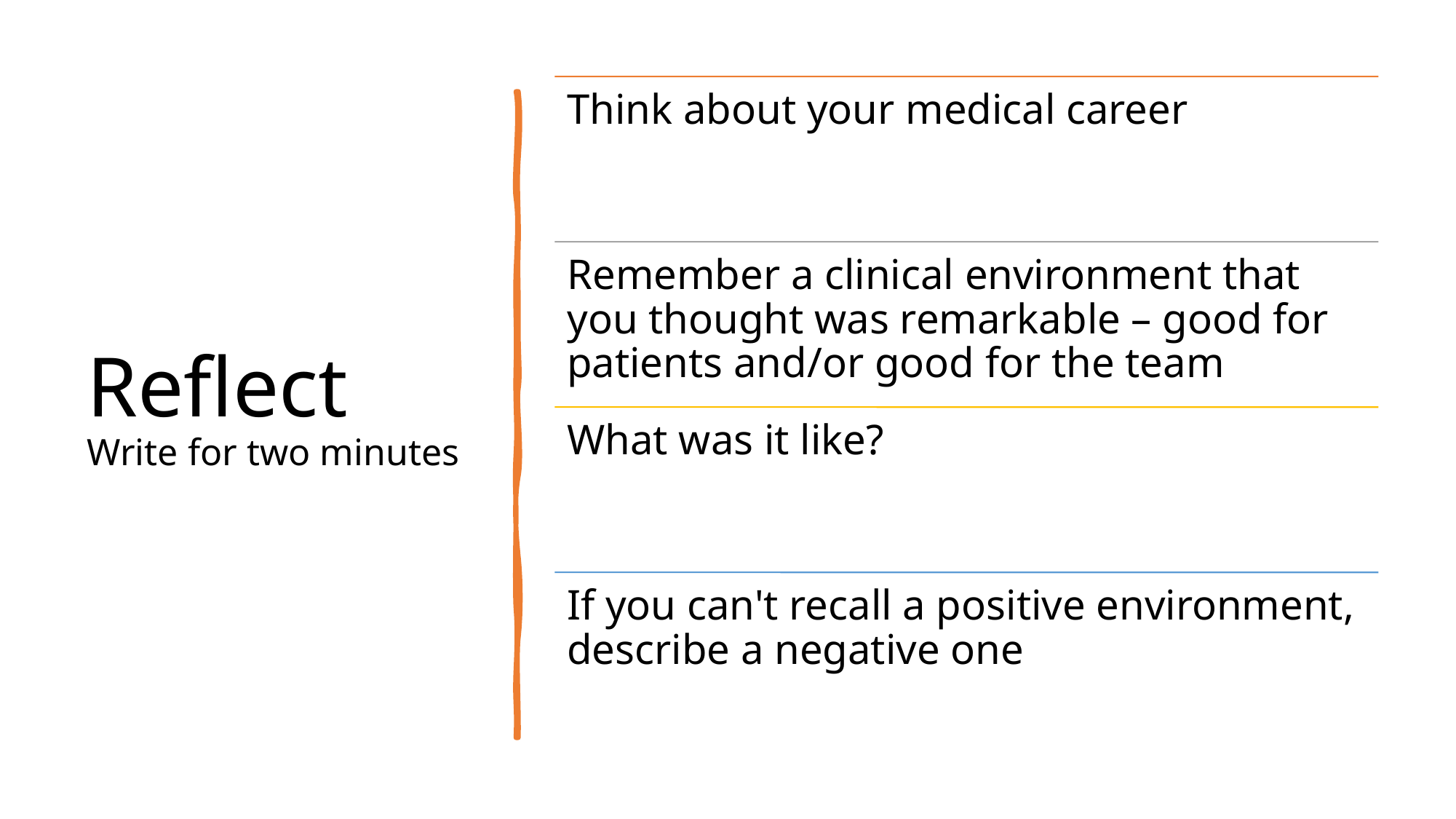

# Reflect Write for two minutes

## Slide 10
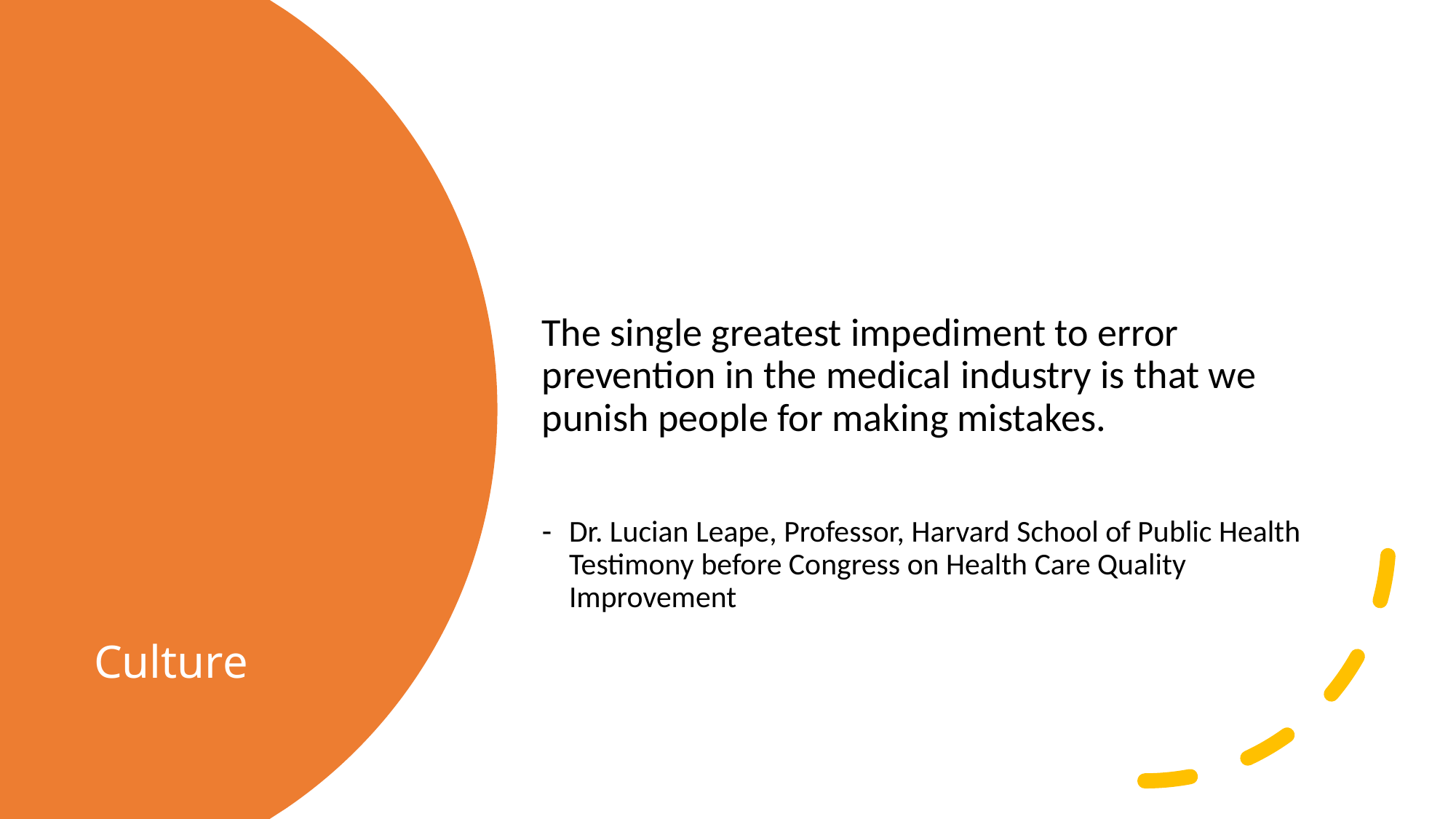

The single greatest impediment to error prevention in the medical industry is that we punish people for making mistakes.
Dr. Lucian Leape, Professor, Harvard School of Public Health Testimony before Congress on Health Care Quality Improvement
# Culture

## Slide 11
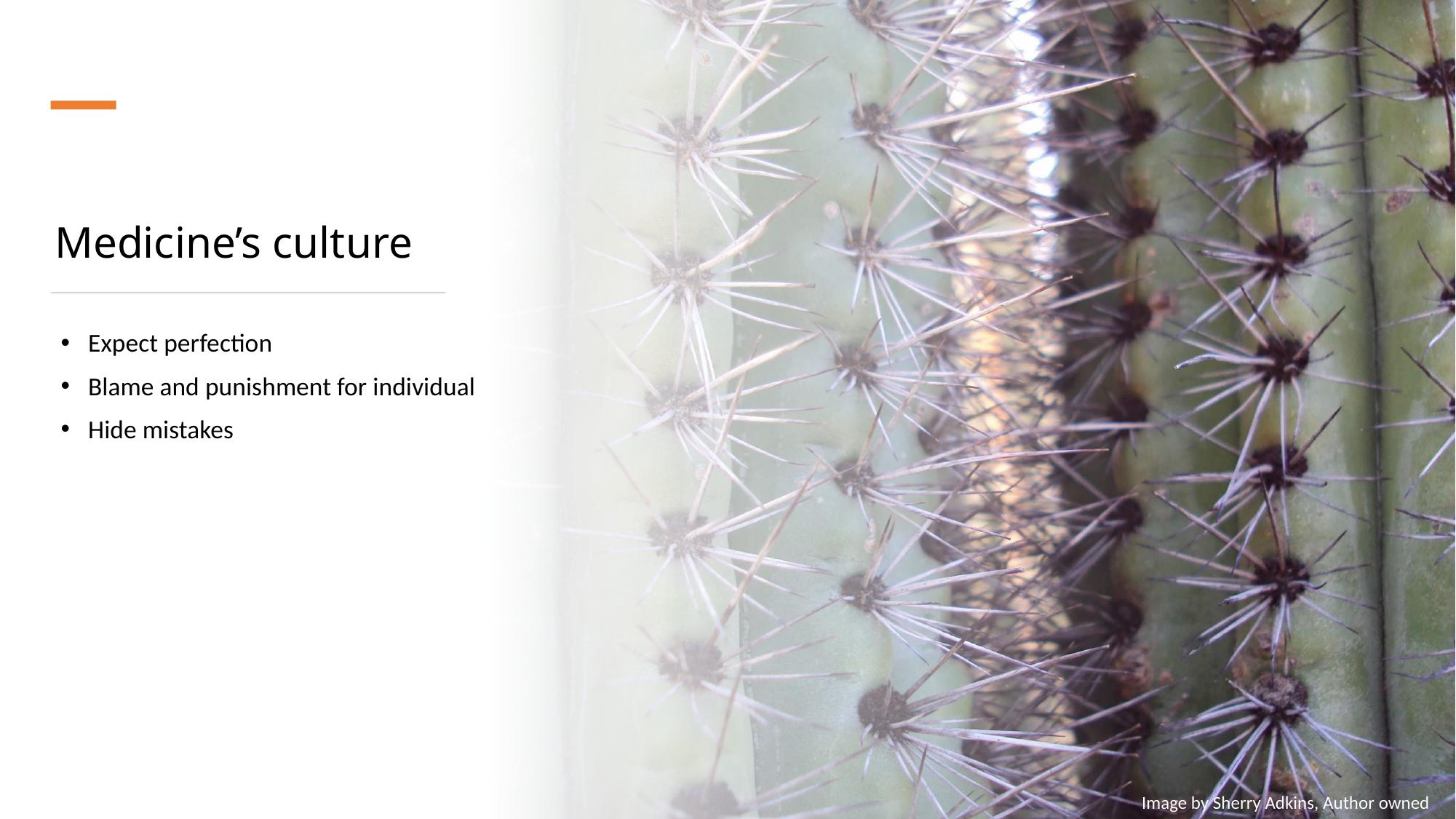

# Medicine’s culture
Expect perfection
Blame and punishment for individual
Hide mistakes
Image by Sherry Adkins, Author owned

## Slide 12
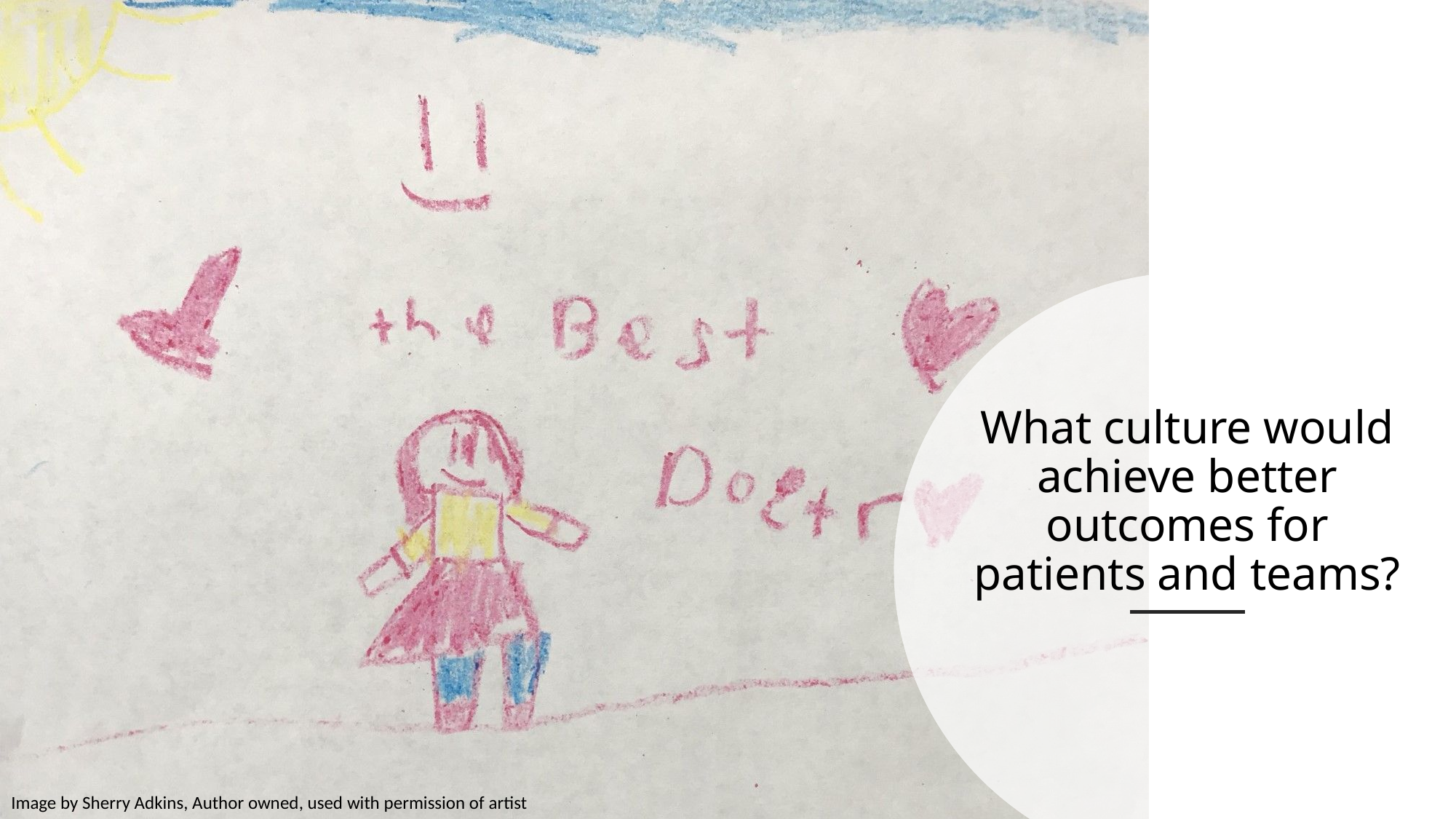

# What culture would achieve better outcomes for patients and teams?
Image by Sherry Adkins, Author owned, used with permission of artist

## Slide 13
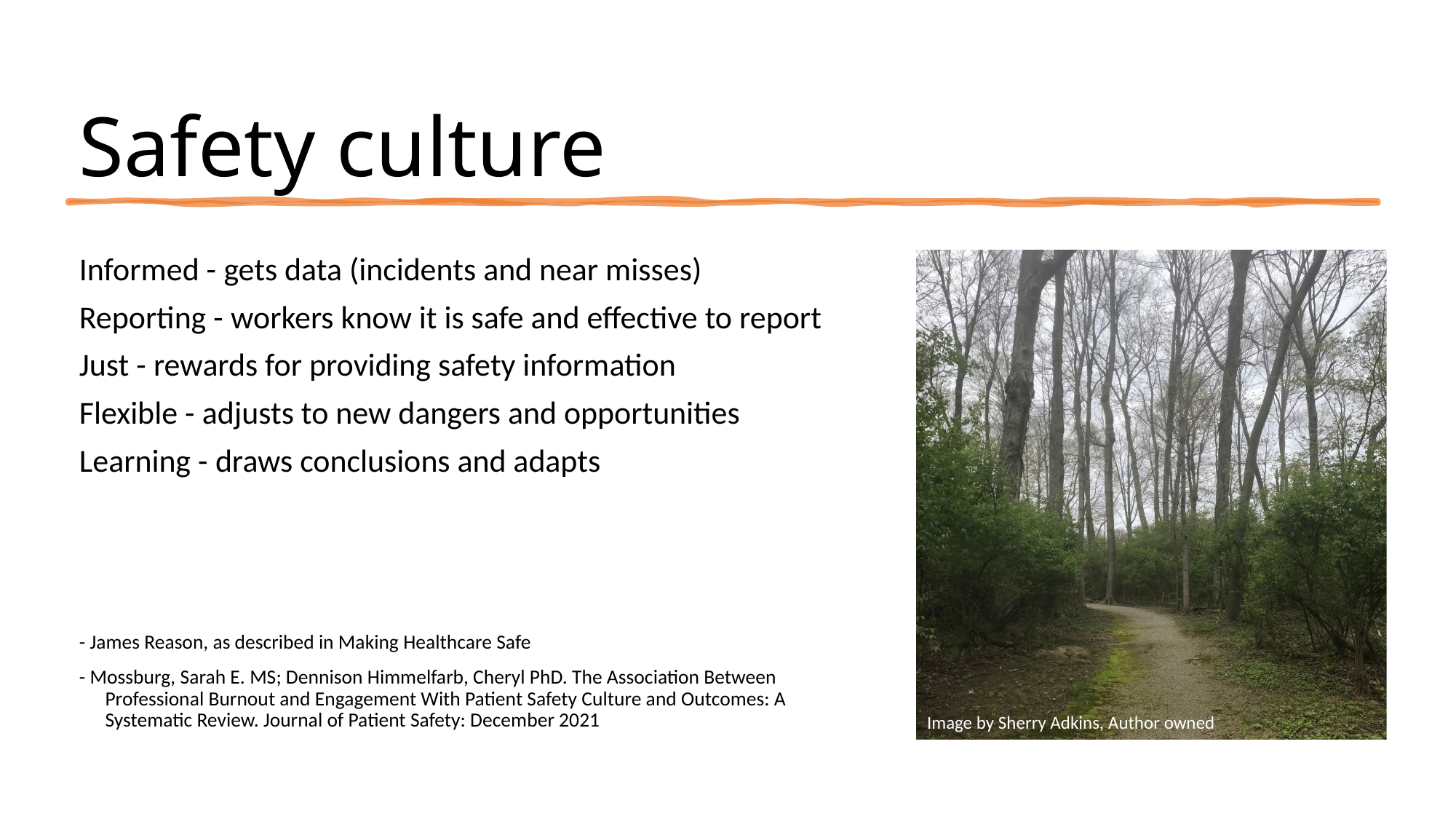

# Safety culture
Informed - gets data (incidents and near misses)
Reporting - workers know it is safe and effective to report
Just - rewards for providing safety information
Flexible - adjusts to new dangers and opportunities
Learning - draws conclusions and adapts
- James Reason, as described in Making Healthcare Safe
- Mossburg, Sarah E. MS; Dennison Himmelfarb, Cheryl PhD. The Association Between Professional Burnout and Engagement With Patient Safety Culture and Outcomes: A Systematic Review. Journal of Patient Safety: December 2021
Image by Sherry Adkins, Author owned

## Slide 14
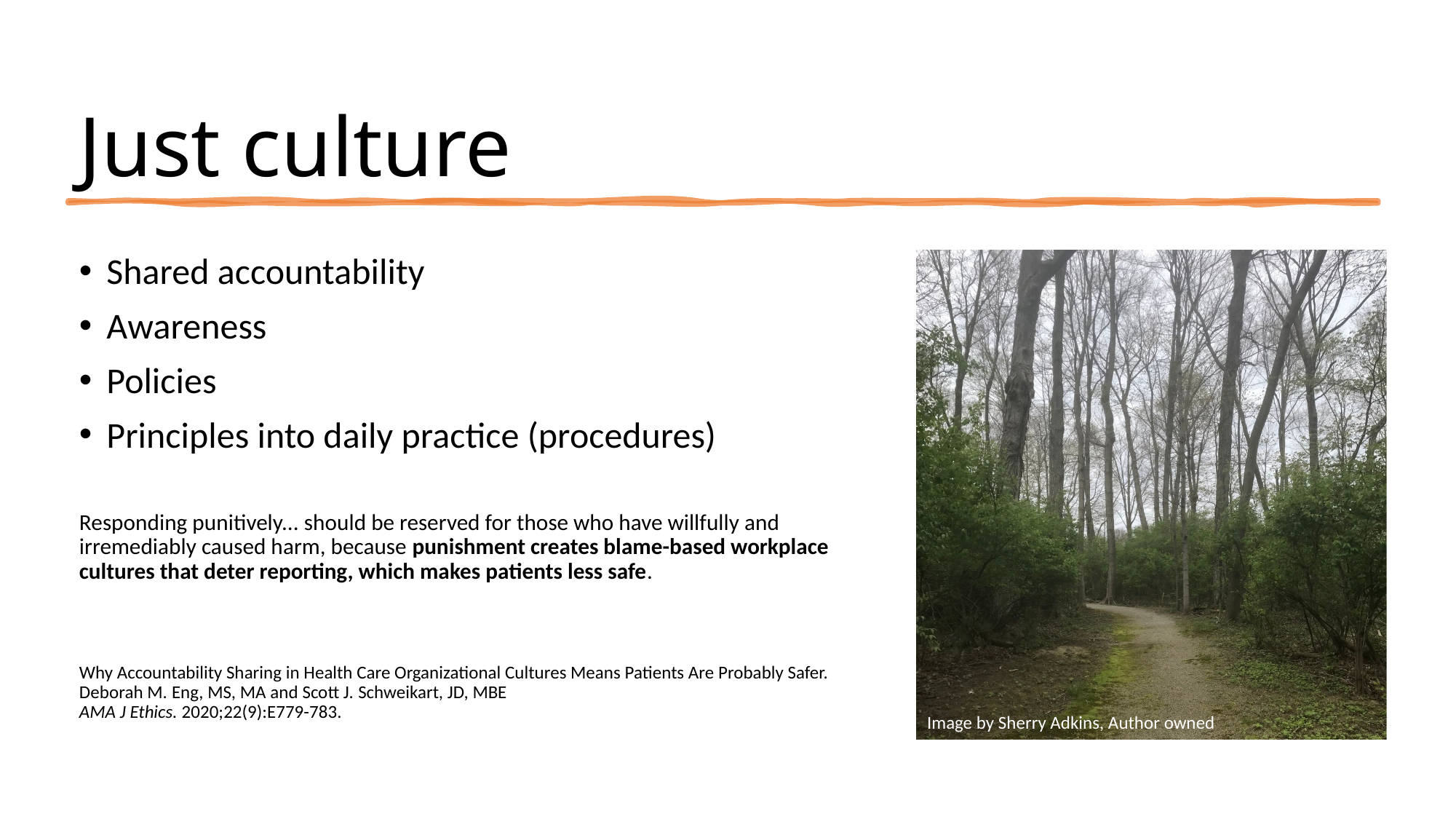

# Just culture
Shared accountability
Awareness
Policies
Principles into daily practice (procedures)
Responding punitively... should be reserved for those who have willfully and irremediably caused harm, because punishment creates blame-based workplace cultures that deter reporting, which makes patients less safe.
Why Accountability Sharing in Health Care Organizational Cultures Means Patients Are Probably Safer.  Deborah M. Eng, MS, MA and Scott J. Schweikart, JD, MBE
AMA J Ethics. 2020;22(9):E779-783.
Image by Sherry Adkins, Author owned

## Slide 15
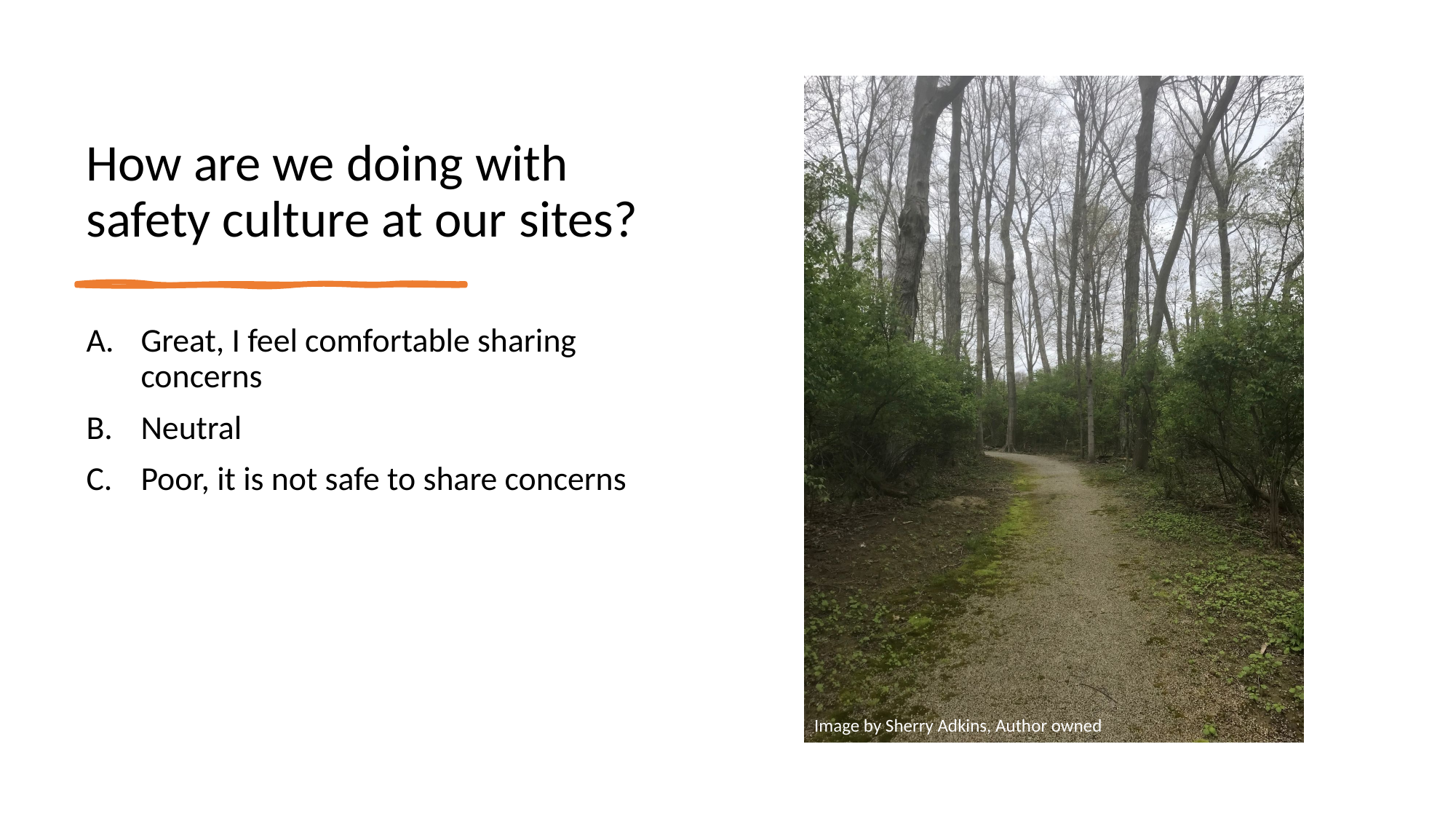

# How are we doing with safety culture at our sites?
Great, I feel comfortable sharing concerns
Neutral
Poor, it is not safe to share concerns
Image by Sherry Adkins, Author owned
Image by Sherry Adkins, Author owned

## Slide 16
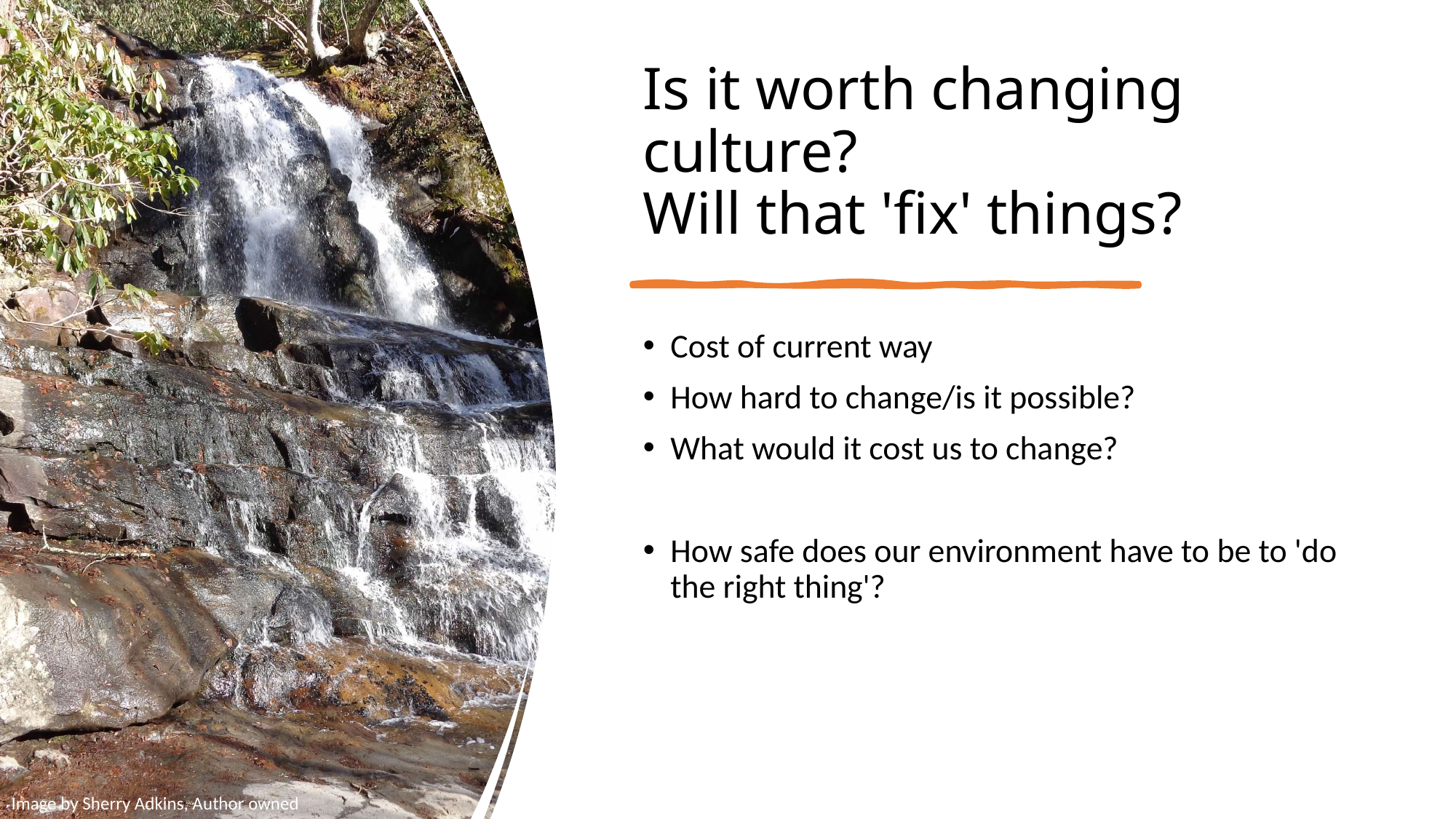

# Is it worth changing culture? Will that 'fix' things?
Cost of current way
How hard to change/is it possible?
What would it cost us to change?
How safe does our environment have to be to 'do the right thing'?
Image by Sherry Adkins, Author owned

## Slide 17
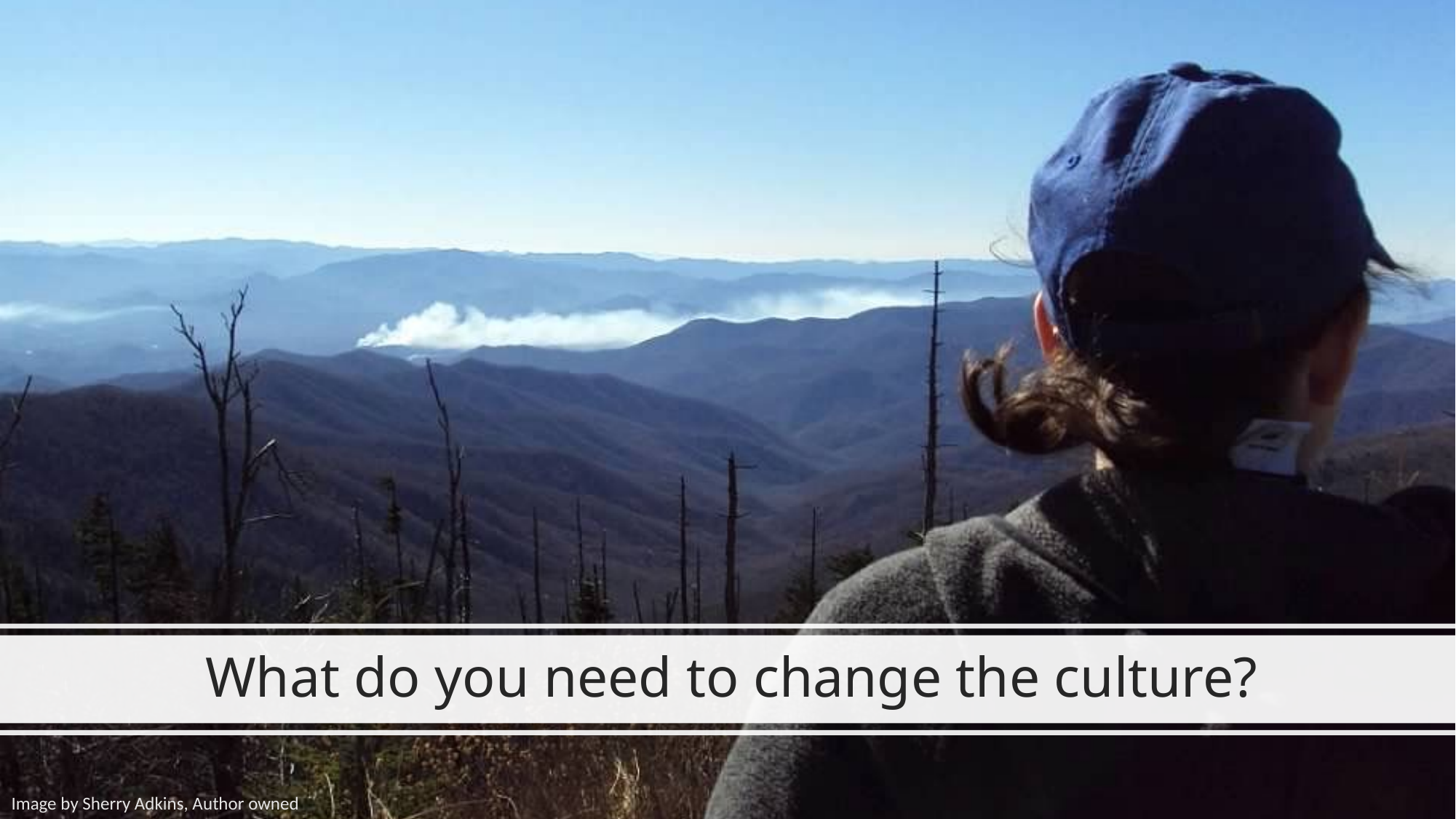

# What do you need to change the culture?
Image by Sherry Adkins, Author owned

## Slide 18
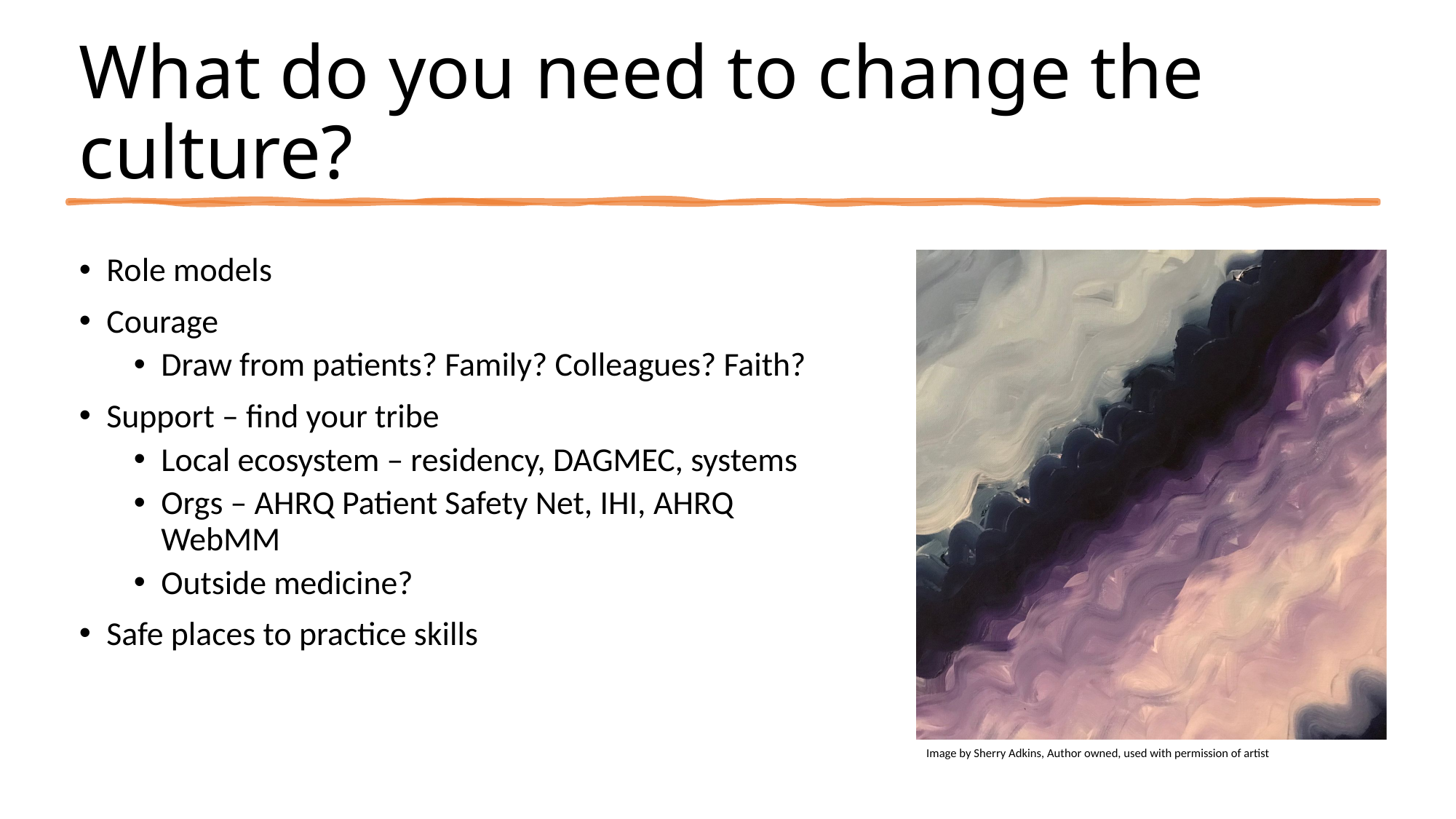

# What do you need to change the culture?
Role models
Courage
Draw from patients? Family? Colleagues? Faith?
Support – find your tribe
Local ecosystem – residency, DAGMEC, systems
Orgs – AHRQ Patient Safety Net, IHI, AHRQ WebMM
Outside medicine?
Safe places to practice skills
Image by Sherry Adkins, Author owned, used with permission of artist

## Slide 19
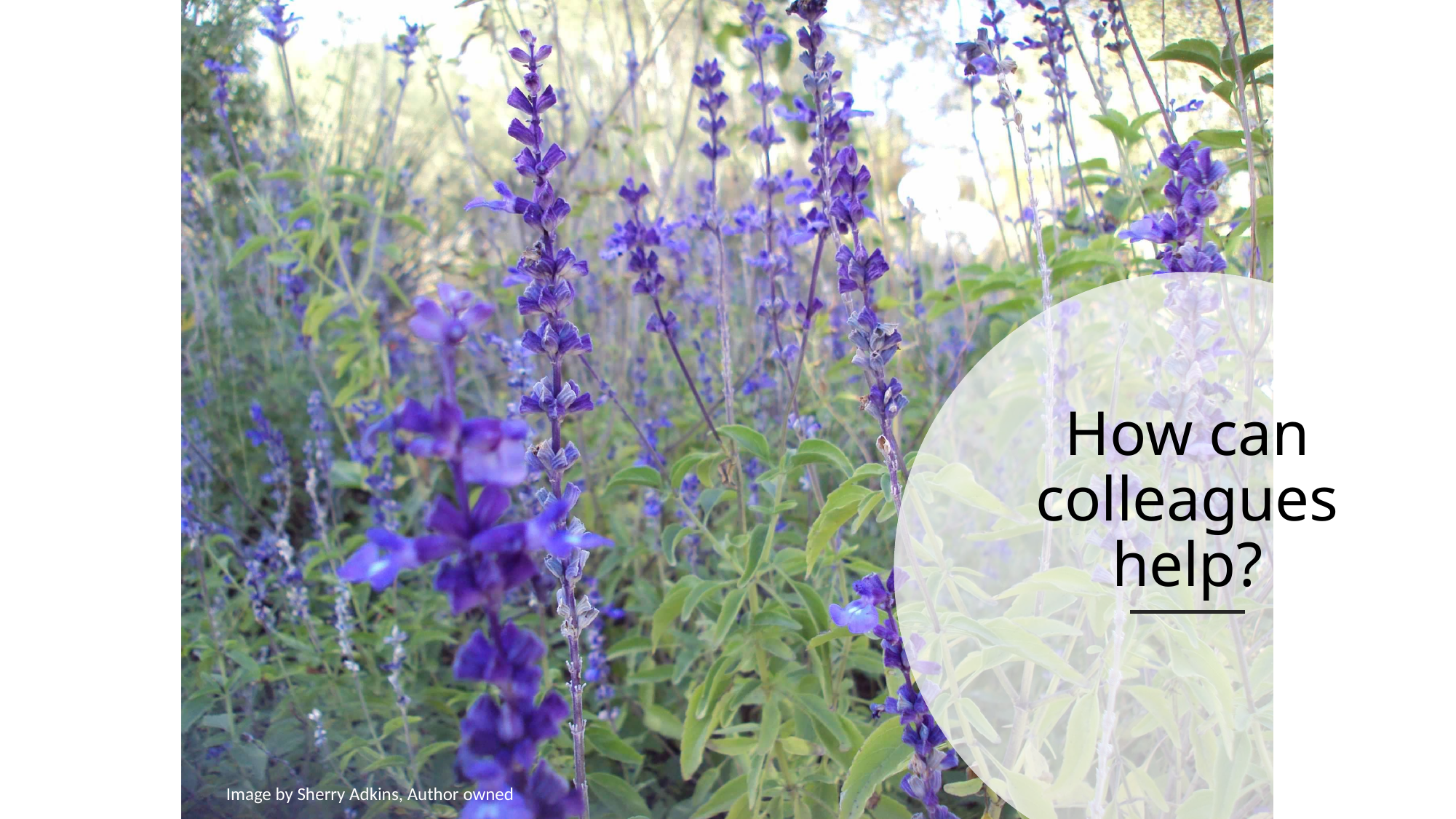

# How can colleagues help?
Image by Sherry Adkins, Author owned

## Slide 20
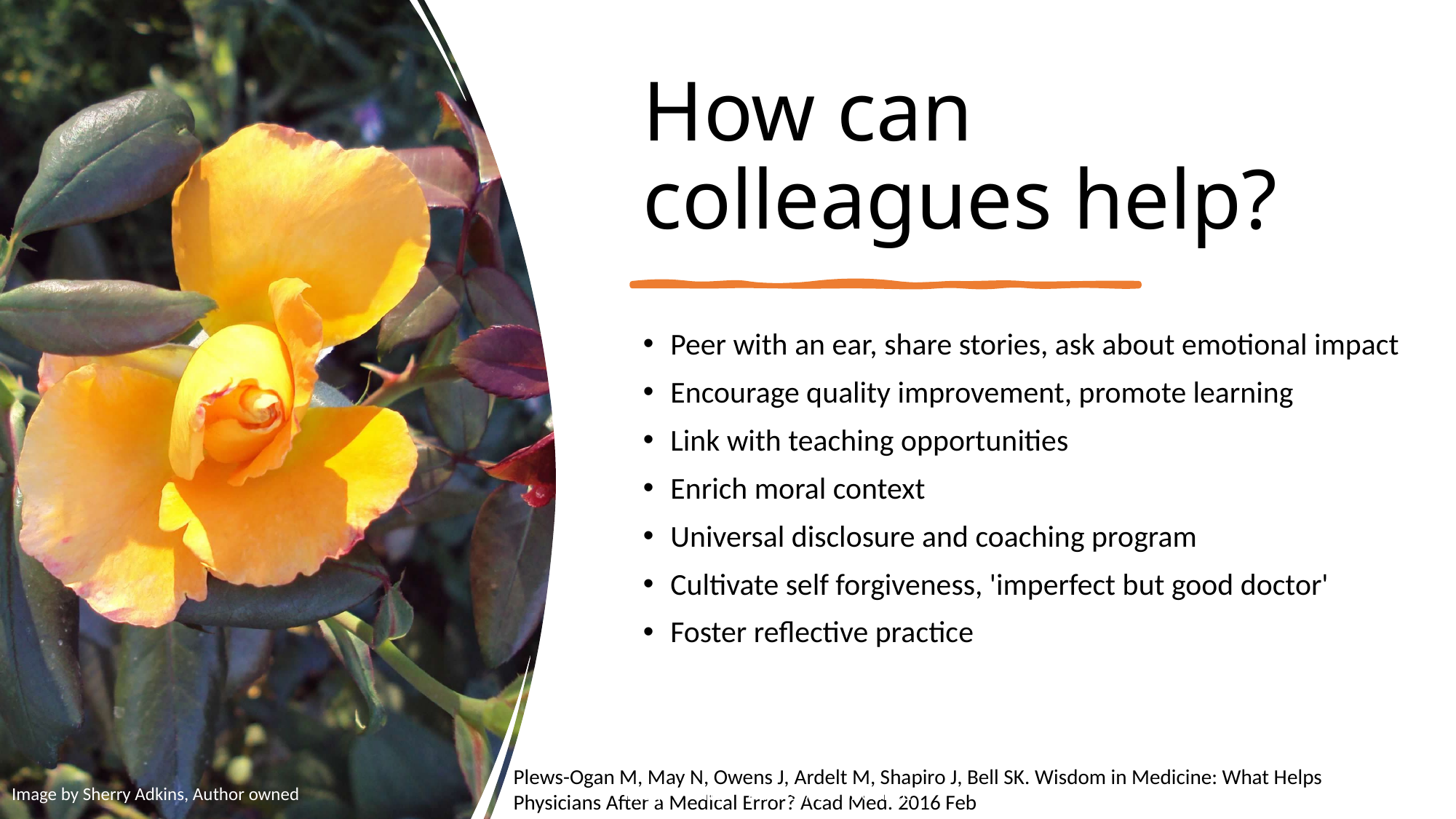

# How can colleagues help?
Peer with an ear, share stories, ask about emotional impact
Encourage quality improvement, promote learning
Link with teaching opportunities
Enrich moral context
Universal disclosure and coaching program
Cultivate self forgiveness, 'imperfect but good doctor'
Foster reflective practice
Plews-Ogan M, May N, Owens J, Ardelt M, Shapiro J, Bell SK. Wisdom in Medicine: What Helps Physicians After a Medical Error? Acad Med. 2016 Feb
Image by Sherry Adkins, Author owned
Image by Sherry Adkins, Author owned

## Slide 21
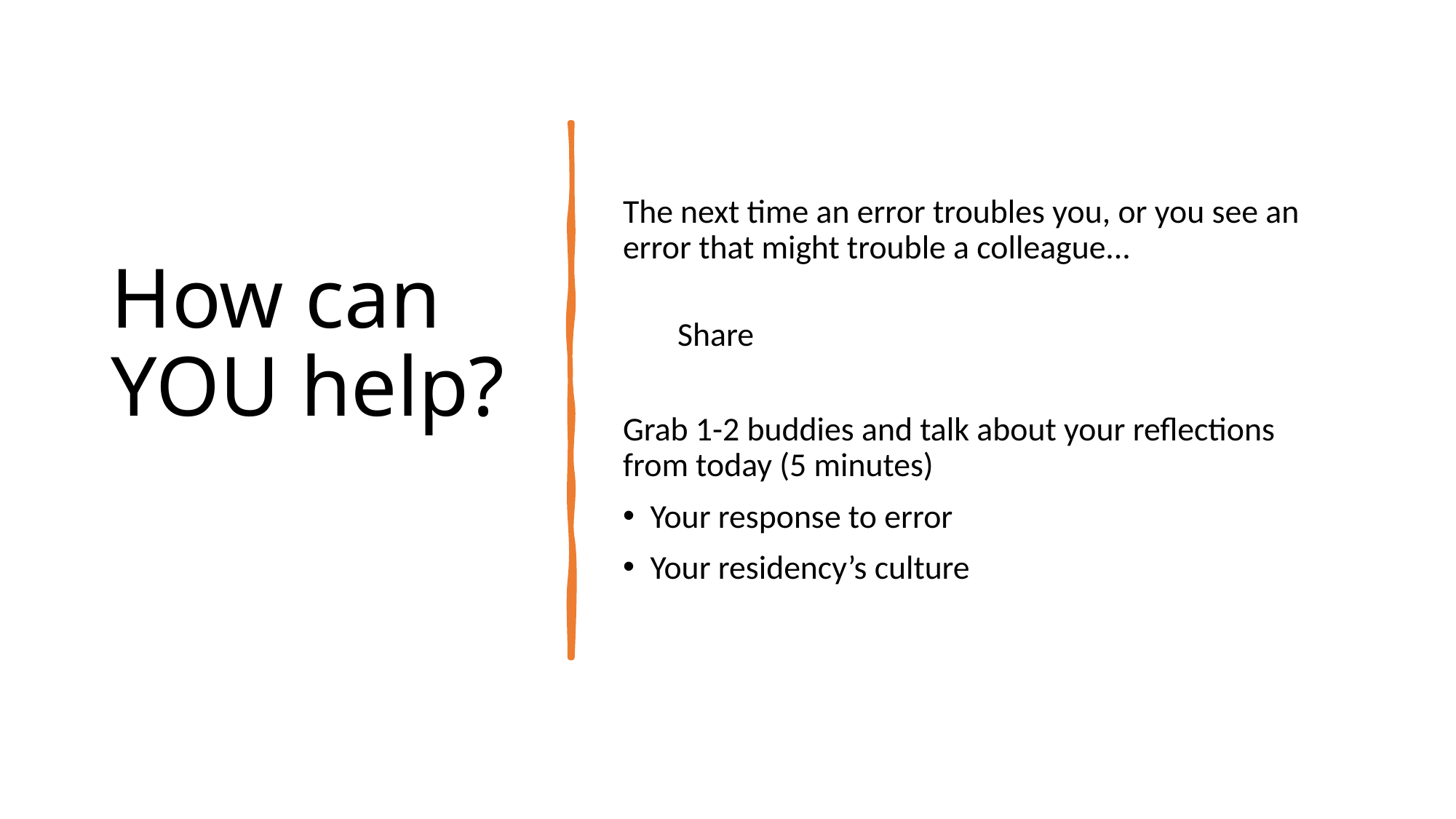

# How can YOU help?
The next time an error troubles you, or you see an error that might trouble a colleague...
Share
Grab 1-2 buddies and talk about your reflections from today (5 minutes)
Your response to error
Your residency’s culture

## Slide 22
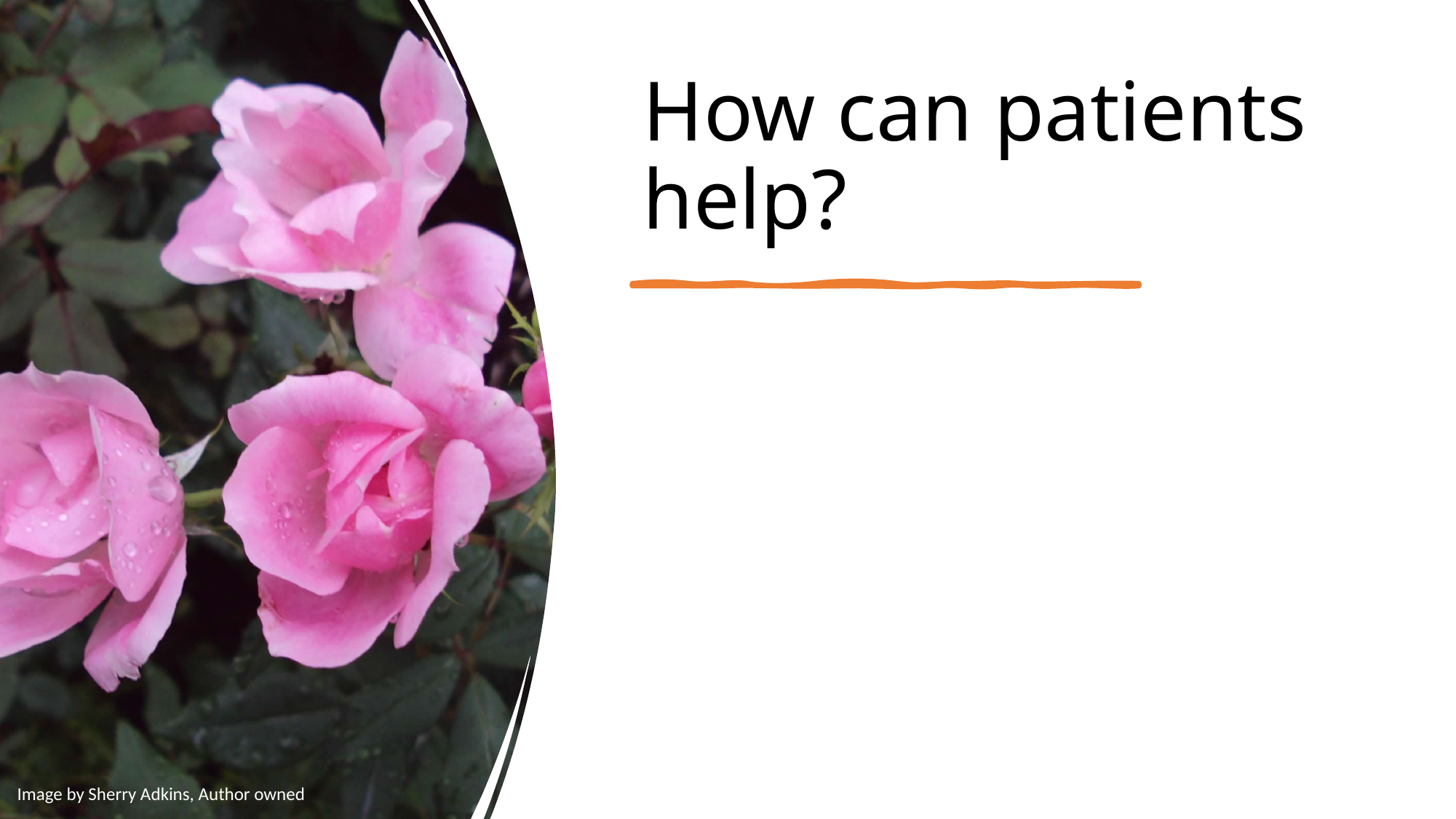

# How can patients help?
Image by Sherry Adkins, Author owned

## Slide 23
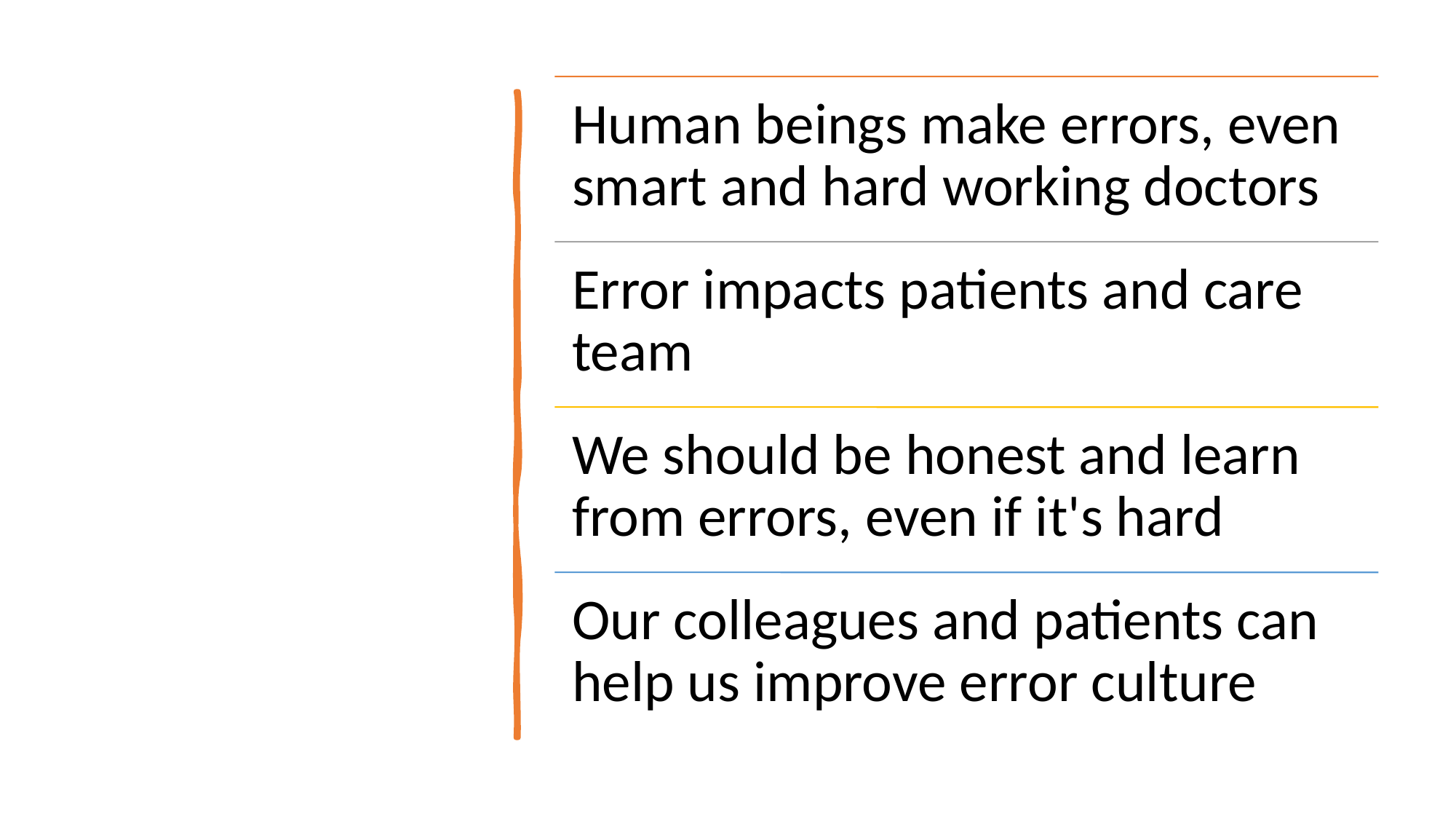

#

## Slide 24
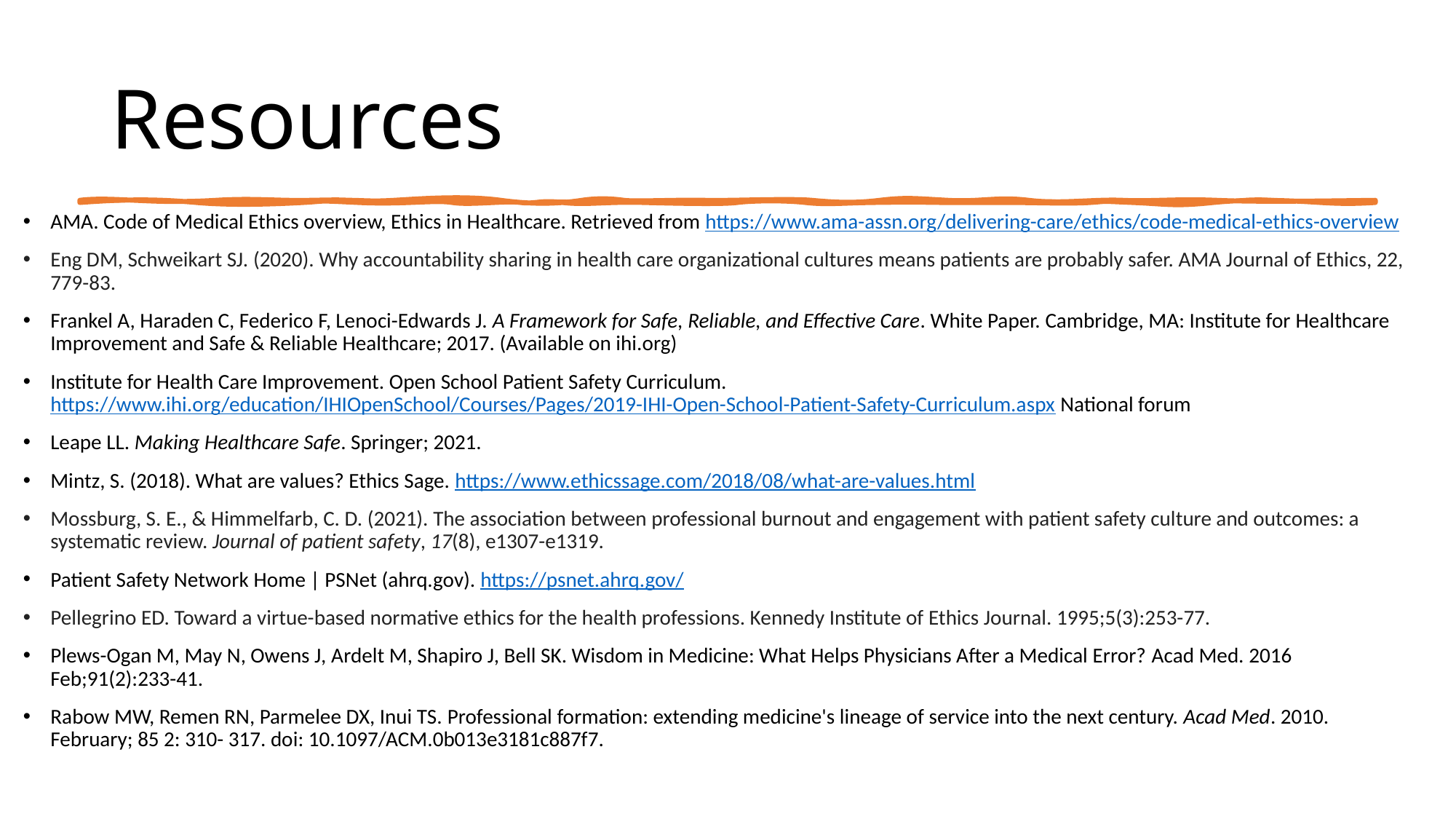

# Resources
AMA. Code of Medical Ethics overview, Ethics in Healthcare. Retrieved from https://www.ama-assn.org/delivering-care/ethics/code-medical-ethics-overview
Eng DM, Schweikart SJ. (2020). Why accountability sharing in health care organizational cultures means patients are probably safer. AMA Journal of Ethics, 22, 779-83.
Frankel A, Haraden C, Federico F, Lenoci-Edwards J. A Framework for Safe, Reliable, and Effective Care. White Paper. Cambridge, MA: Institute for Healthcare Improvement and Safe & Reliable Healthcare; 2017. (Available on ihi.org)
Institute for Health Care Improvement. Open School Patient Safety Curriculum. https://www.ihi.org/education/IHIOpenSchool/Courses/Pages/2019-IHI-Open-School-Patient-Safety-Curriculum.aspx National forum
Leape LL. Making Healthcare Safe. Springer; 2021.
Mintz, S. (2018). What are values? Ethics Sage. https://www.ethicssage.com/2018/08/what-are-values.html
Mossburg, S. E., & Himmelfarb, C. D. (2021). The association between professional burnout and engagement with patient safety culture and outcomes: a systematic review. Journal of patient safety, 17(8), e1307-e1319.
Patient Safety Network Home | PSNet (ahrq.gov). https://psnet.ahrq.gov/
Pellegrino ED. Toward a virtue-based normative ethics for the health professions. Kennedy Institute of Ethics Journal. 1995;5(3):253-77.
Plews-Ogan M, May N, Owens J, Ardelt M, Shapiro J, Bell SK. Wisdom in Medicine: What Helps Physicians After a Medical Error? Acad Med. 2016 Feb;91(2):233-41.
Rabow MW, Remen RN, Parmelee DX, Inui TS. Professional formation: extending medicine's lineage of service into the next century. Acad Med. 2010. February; 85 2: 310- 317. doi: 10.1097/ACM.0b013e3181c887f7.
