# Supplementary material for: Medical Error: Using Storytelling and Reflection to Impact Resident Error Response Factors
Source: MedEdPORTAL. 2024 Oct 10;20:11451. doi: 10.15766/mep_2374-8265.11451 (PMC11466310; doi:10.15766/mep_2374-8265.11451)
Supplement: Supplementary file 1 — Facilitators Guide.docxError Session 1.pptxError Session 1 Handout.pdfError Session 2.pptxError Session 3.pptxError Session 3 Handout - Error Cases.docxFaculty Survey.docxPremodule Resident Survey.docxPostmodule Resident Survey.docx [file mep_2374-8265.11451-s001.zip › E. Error Session 3.pptx]

## Slide 1
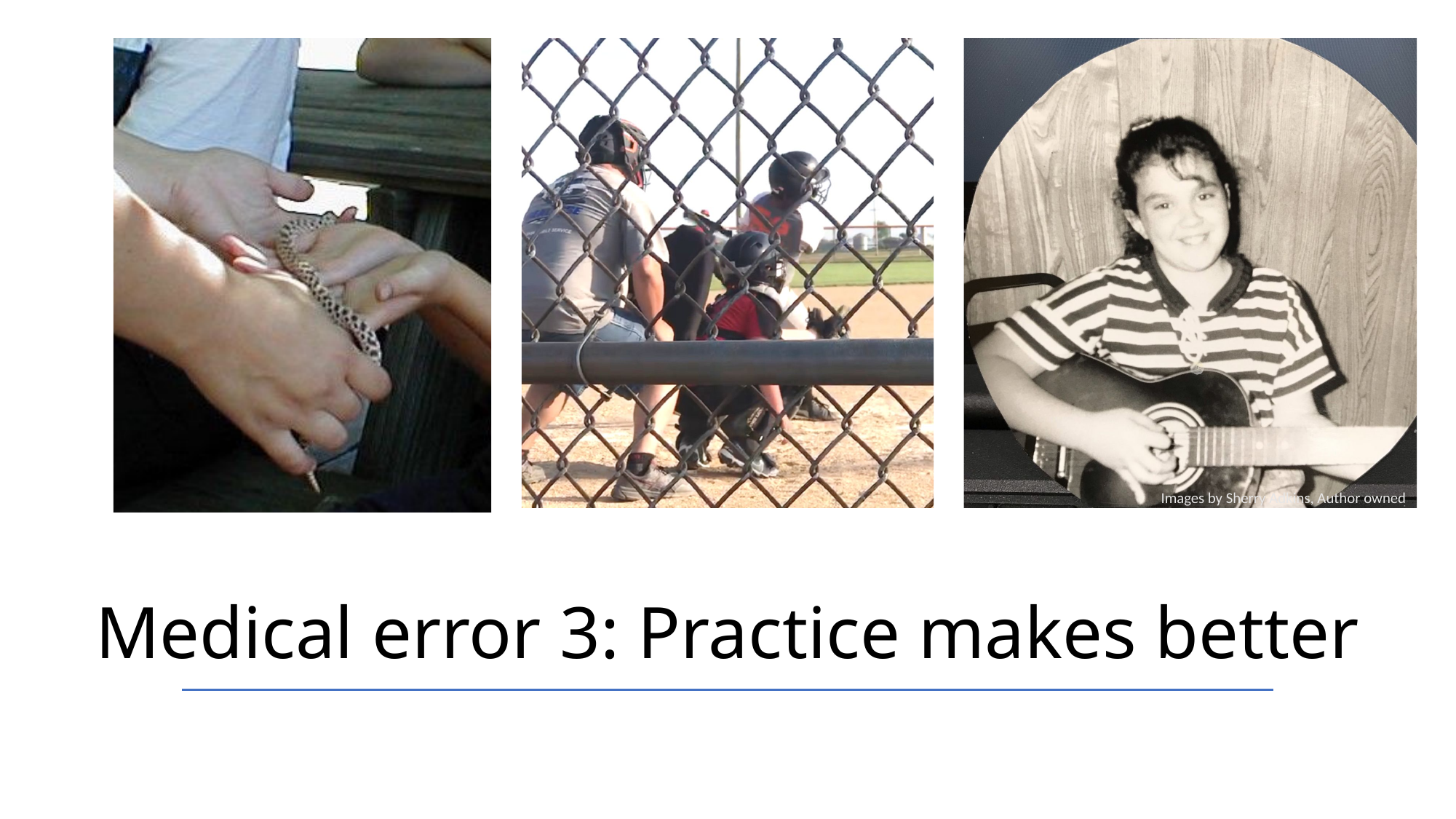

Images by Sherry Adkins, Author owned
# Medical error 3: Practice makes better

## Slide 2
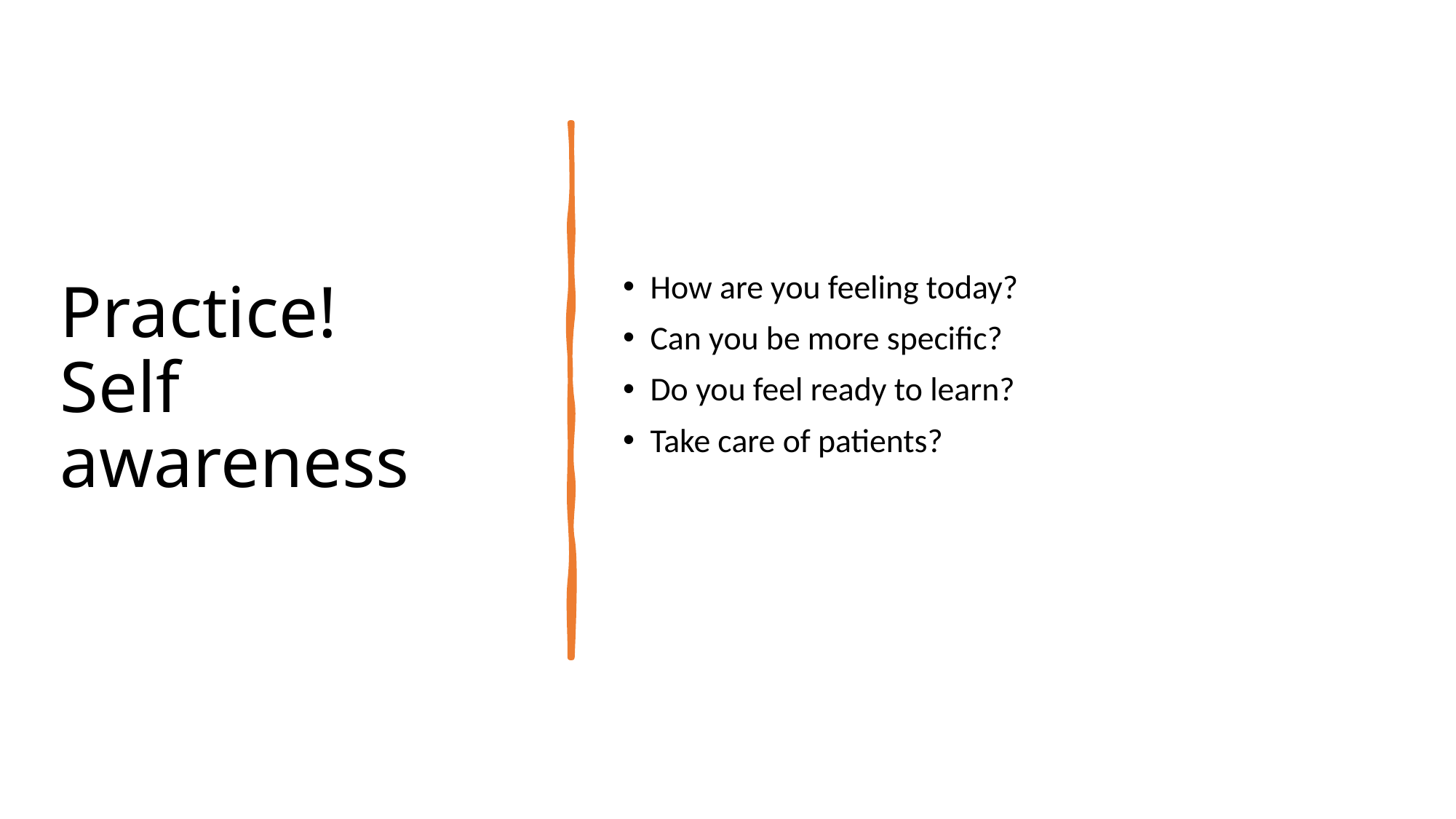

# Practice!  Self awareness
How are you feeling today?
Can you be more specific?
Do you feel ready to learn?
Take care of patients?

## Slide 3
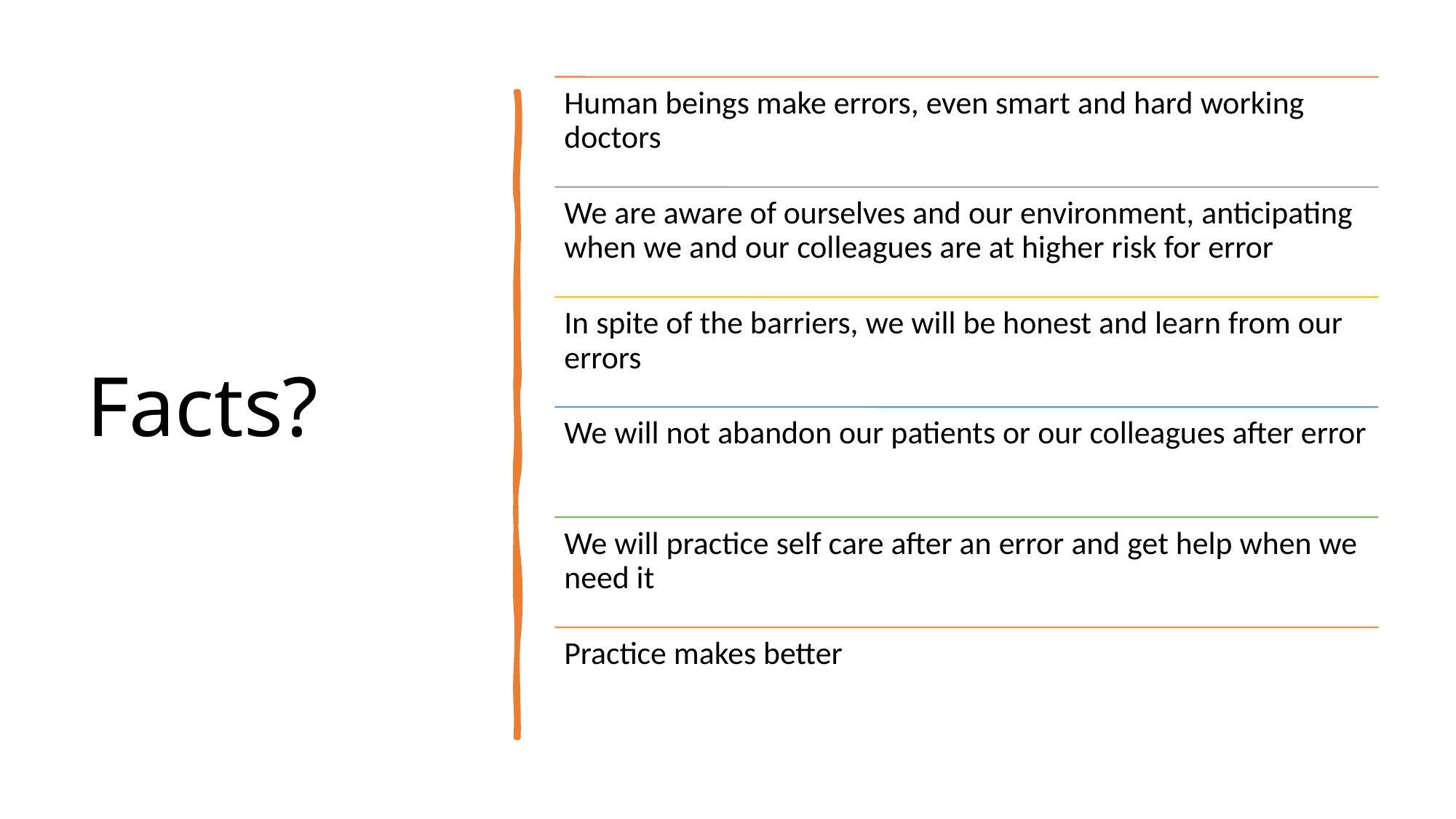

# Facts?

## Slide 4
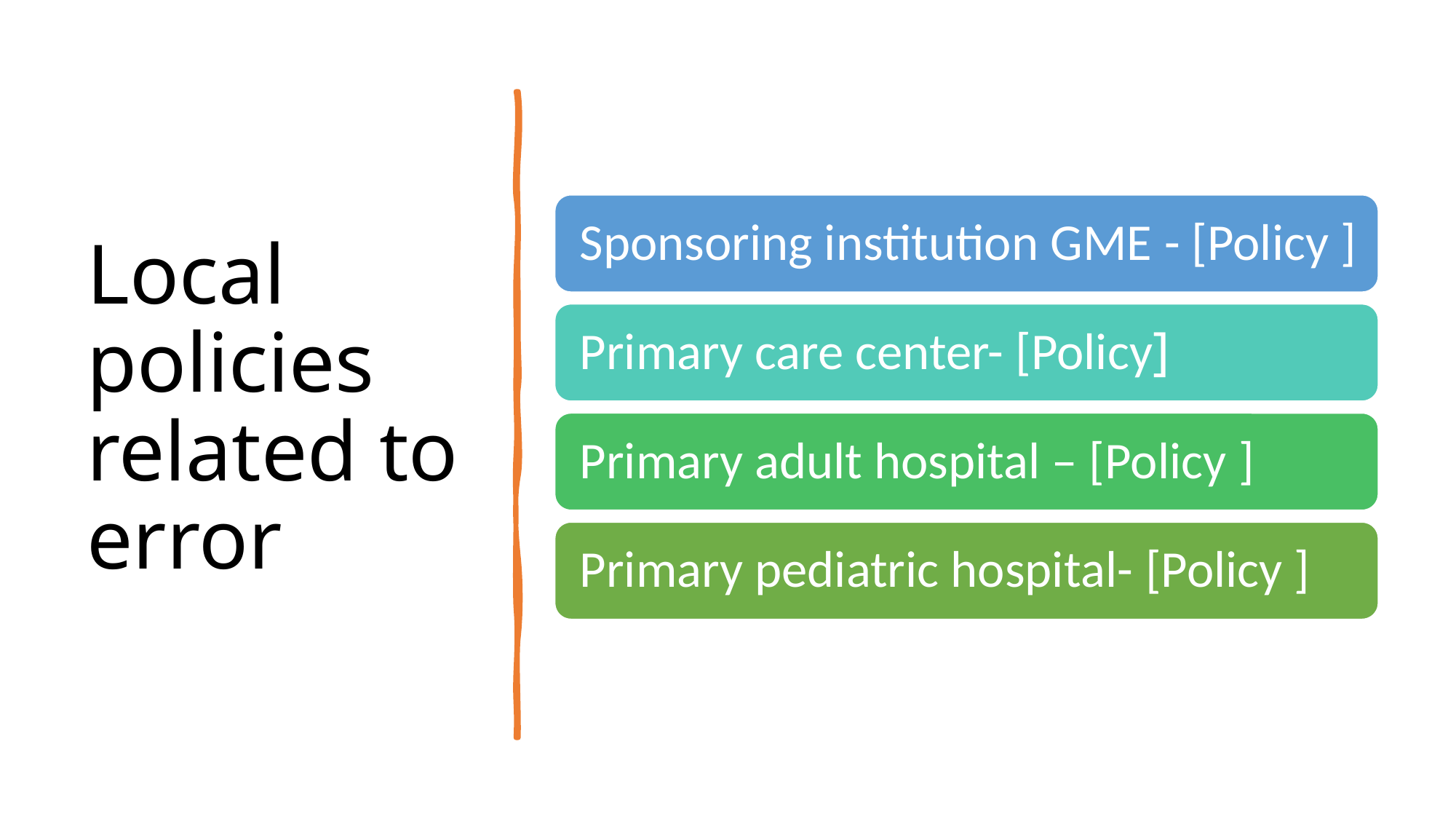

# Local policies related to error

## Slide 5
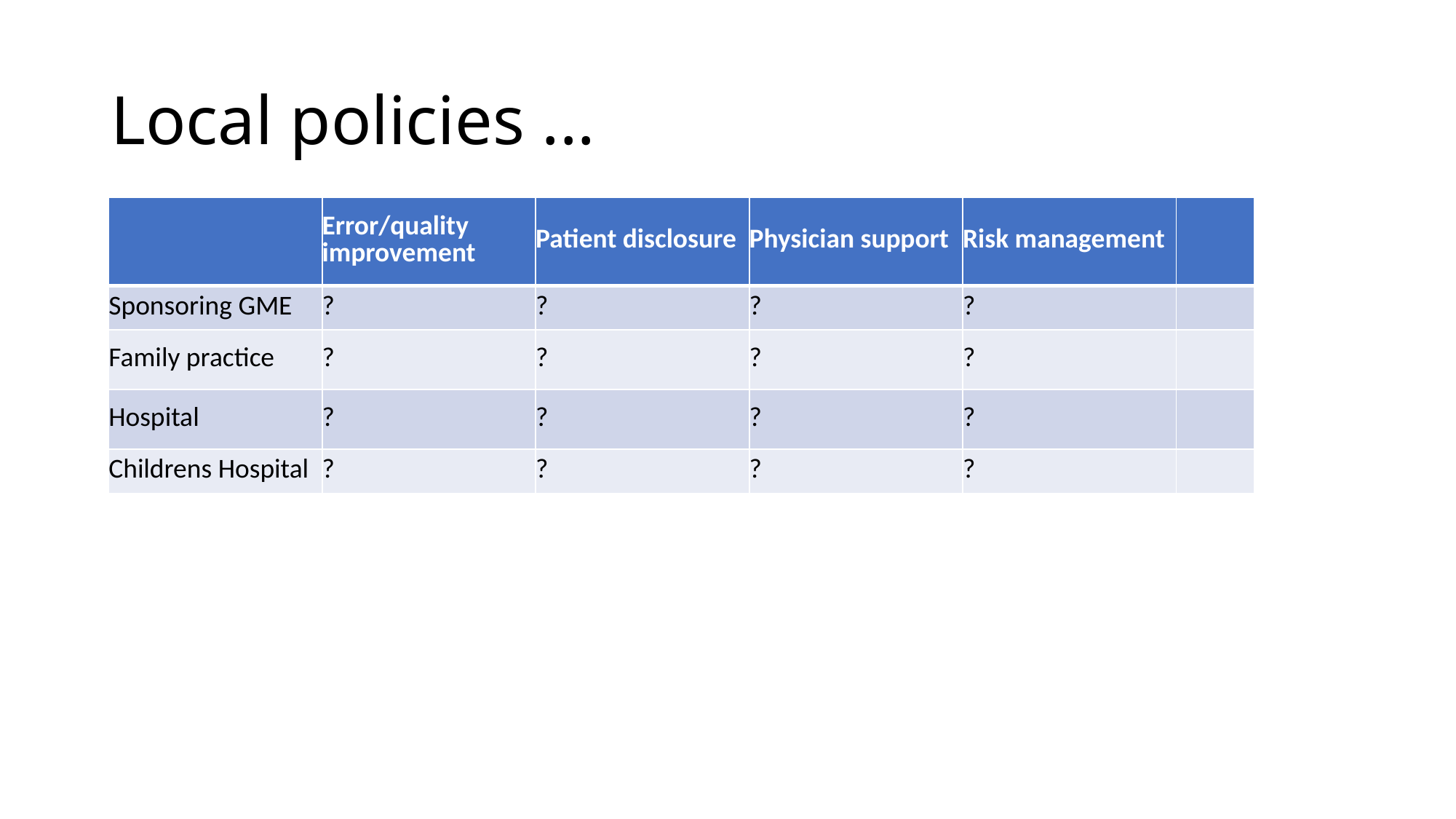

# Local policies ...
| | Error/quality improvement | Patient disclosure | Physician support | Risk management | |
| --- | --- | --- | --- | --- | --- |
| Sponsoring GME | ? | ? | ? | ? | |
| Family practice | ? | ? | ? | ? | |
| Hospital | ? | ? | ? | ? | |
| Childrens Hospital | ? | ? | ? | ? | |

## Slide 6
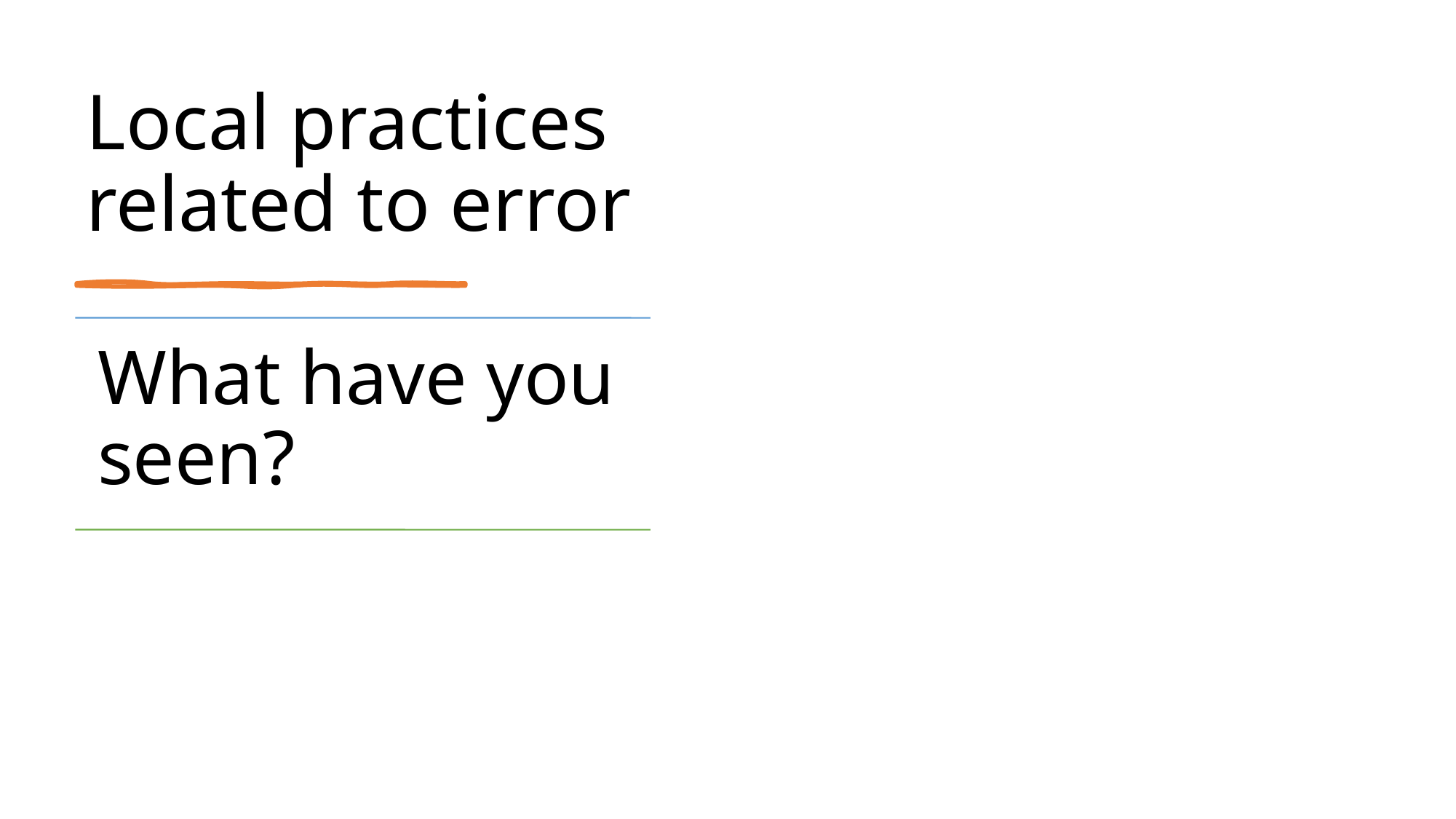

# Local practices related to error

## Slide 7
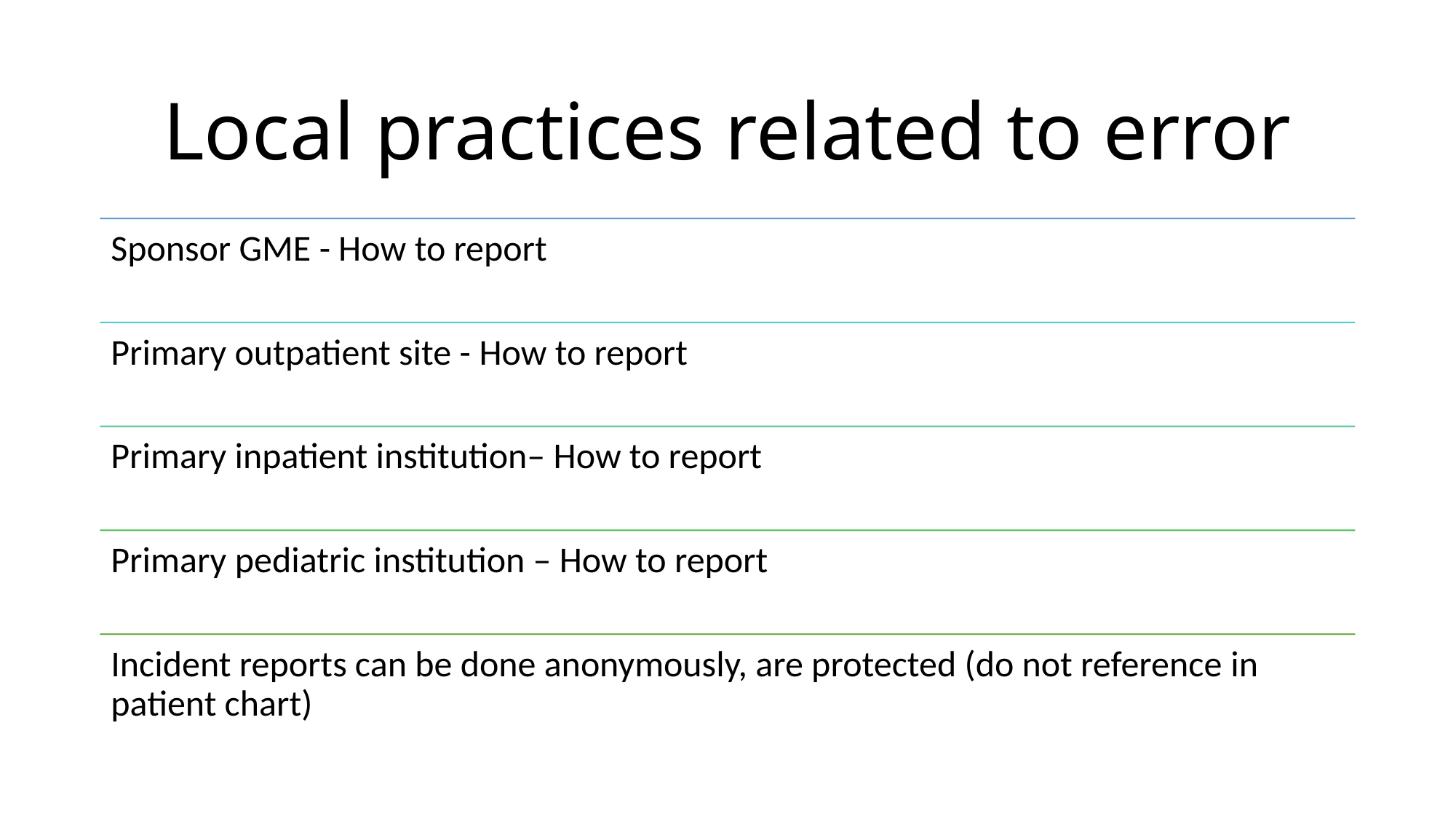

# Local practices related to error

## Slide 8
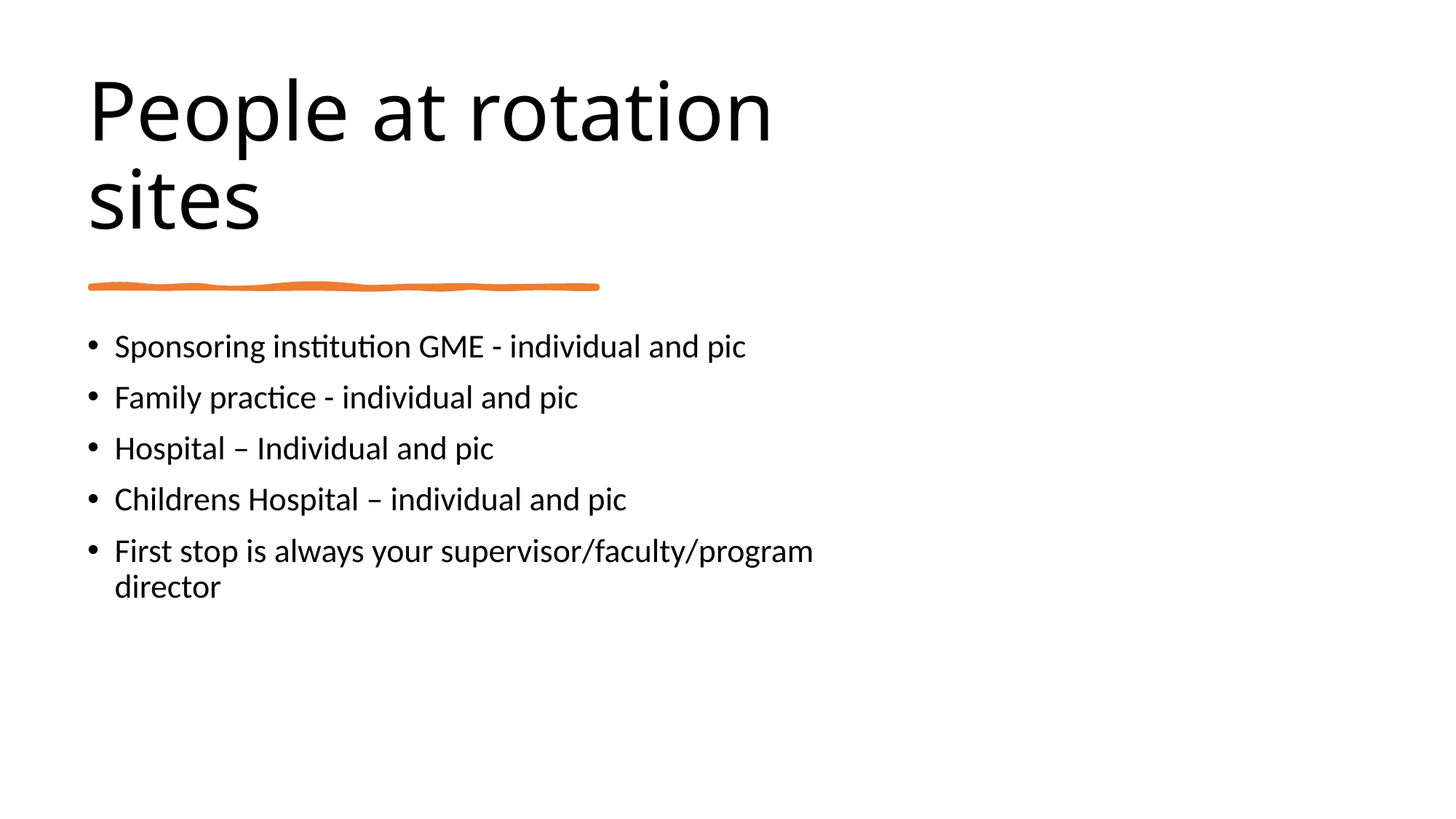

# People at rotation sites
Sponsoring institution GME - individual and pic
Family practice - individual and pic
Hospital – Individual and pic
Childrens Hospital – individual and pic
First stop is always your supervisor/faculty/program director

## Slide 9
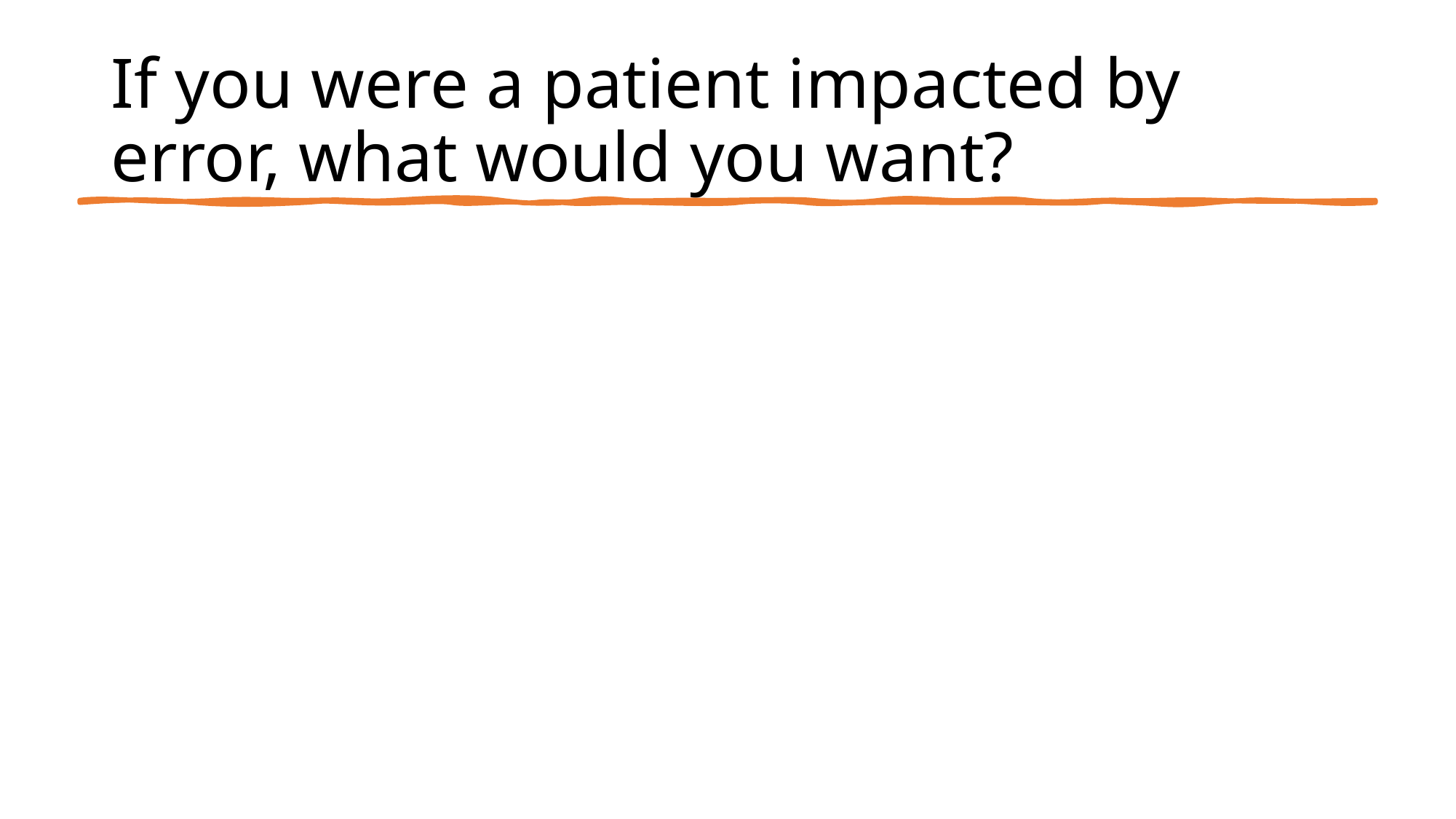

# If you were a patient impacted by error, what would you want?

## Slide 10
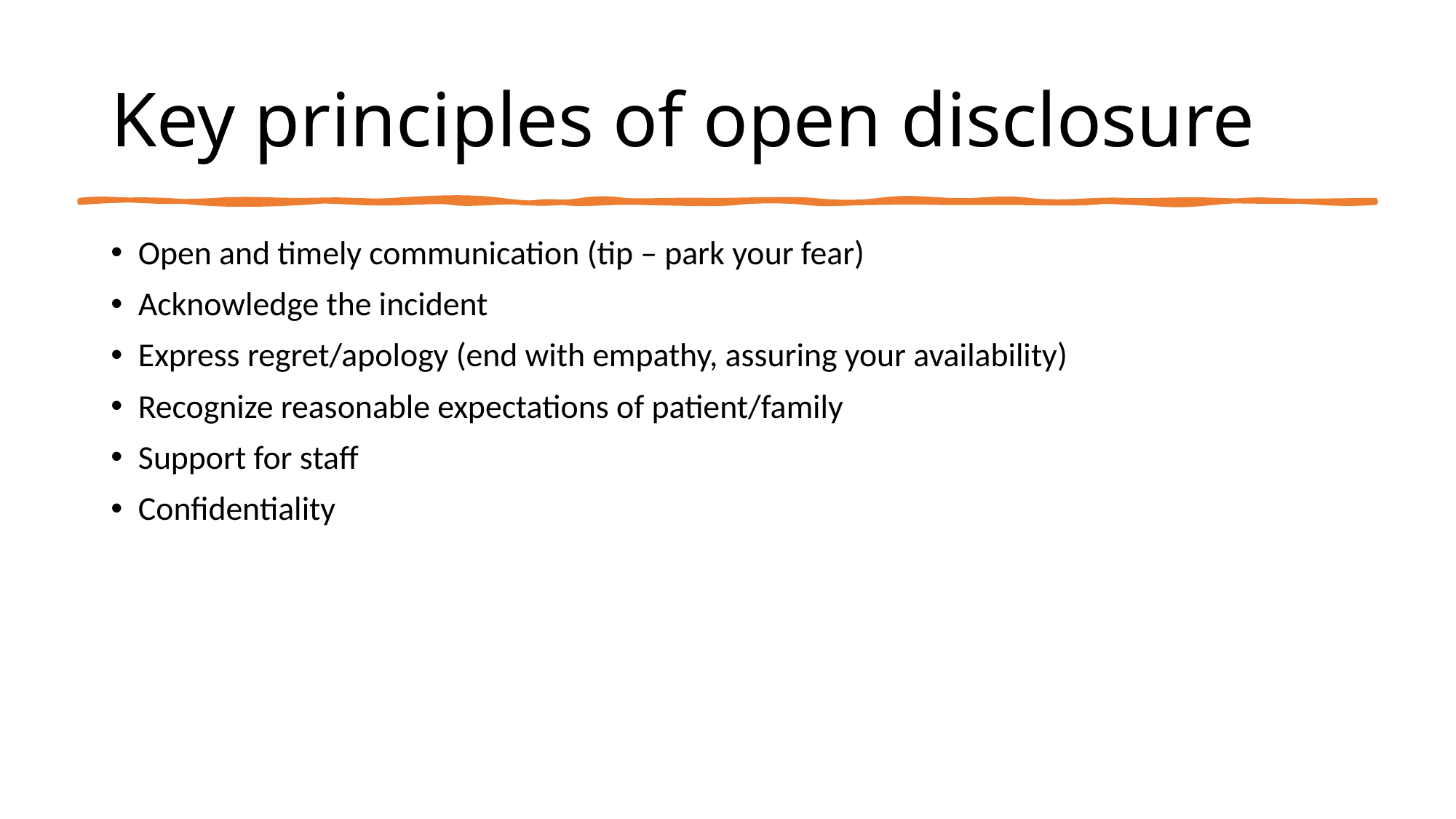

# Key principles of open disclosure
Open and timely communication (tip – park your fear)
Acknowledge the incident
Express regret/apology (end with empathy, assuring your availability)
Recognize reasonable expectations of patient/family
Support for staff
Confidentiality

## Slide 11
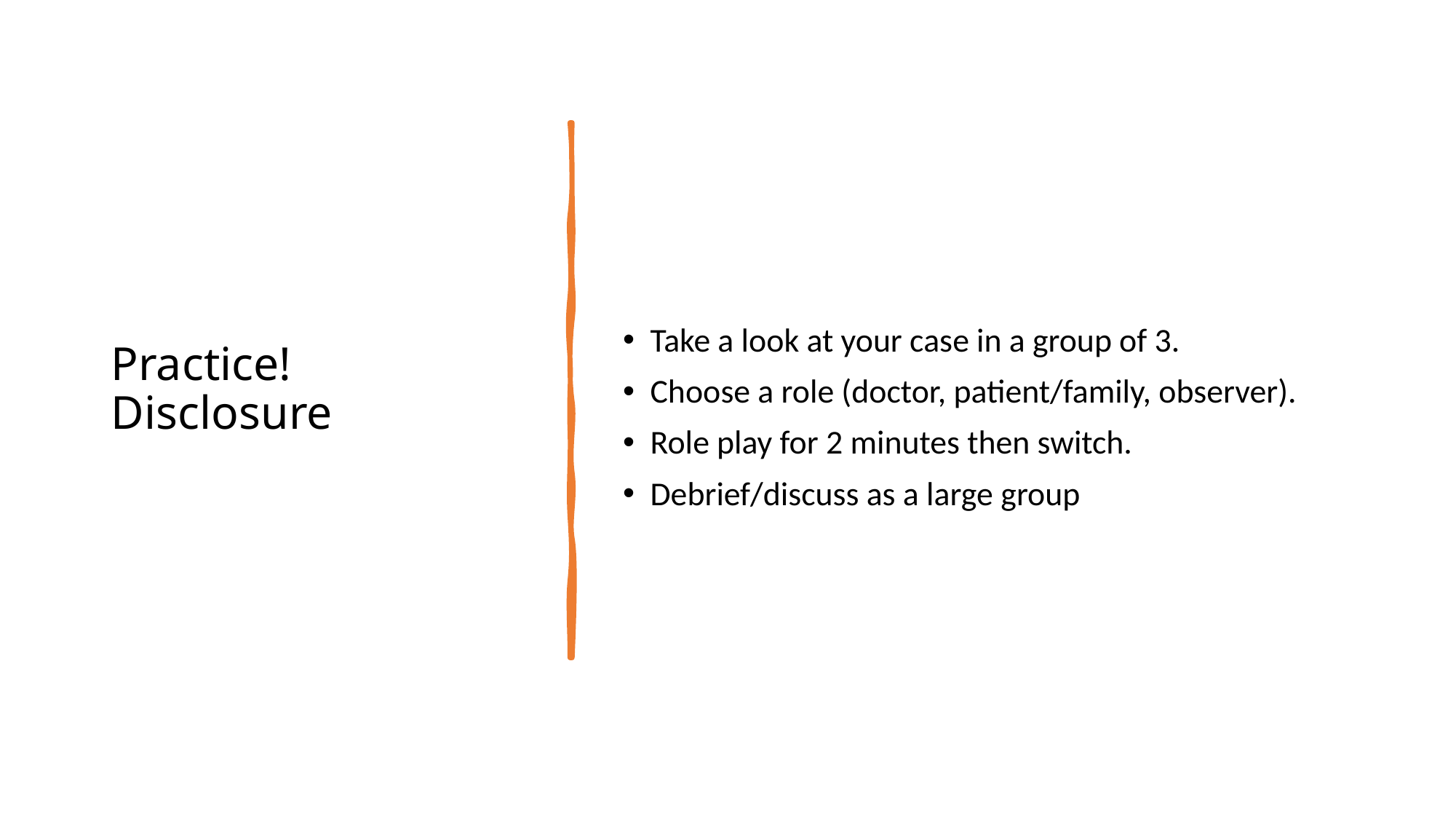

# Practice!  Disclosure
Take a look at your case in a group of 3.
Choose a role (doctor, patient/family, observer).
Role play for 2 minutes then switch.
Debrief/discuss as a large group

## Slide 12
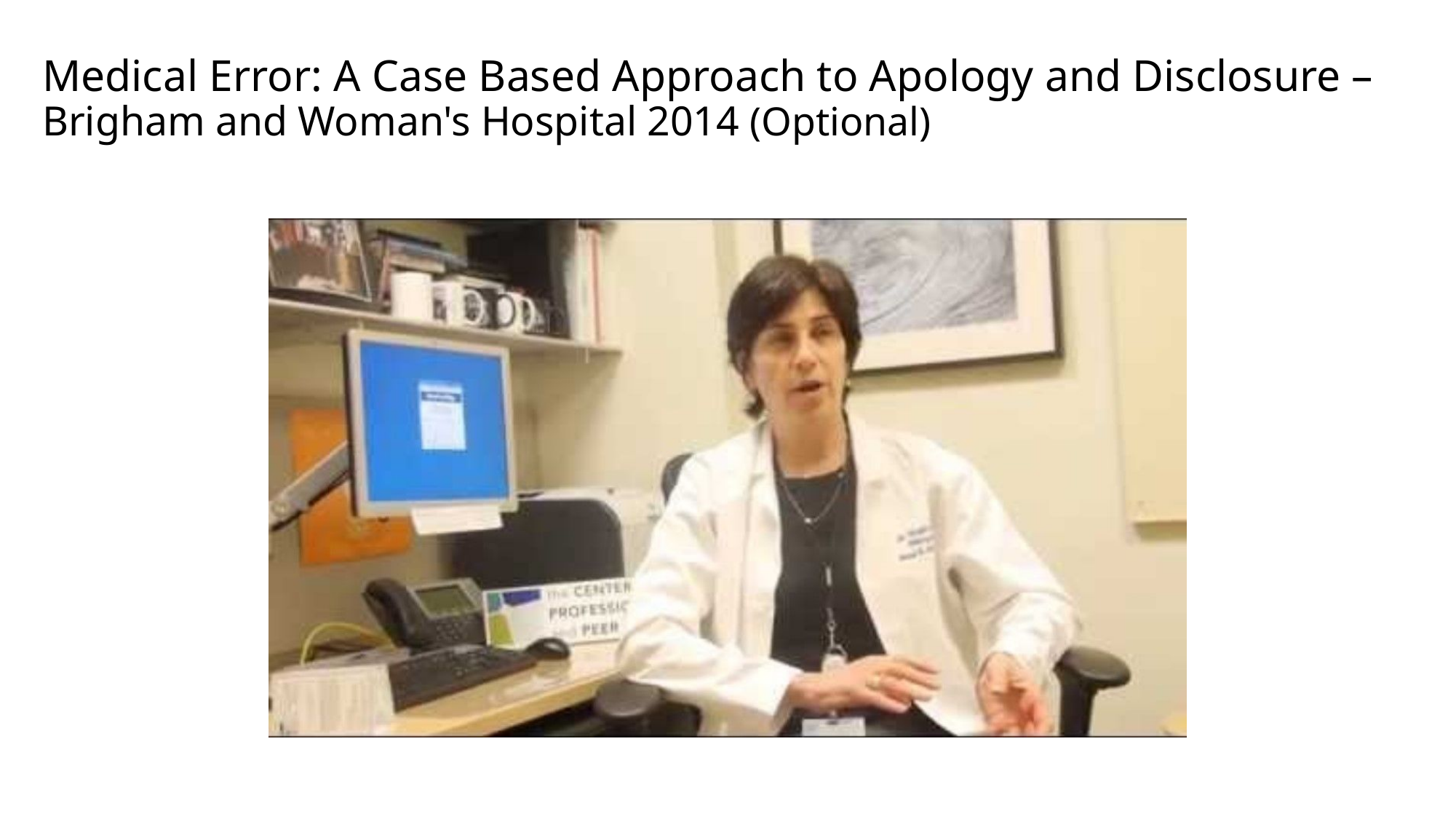

# Medical Error: A Case Based Approach to Apology and Disclosure – Brigham and Woman's Hospital 2014 (Optional)

## Slide 13
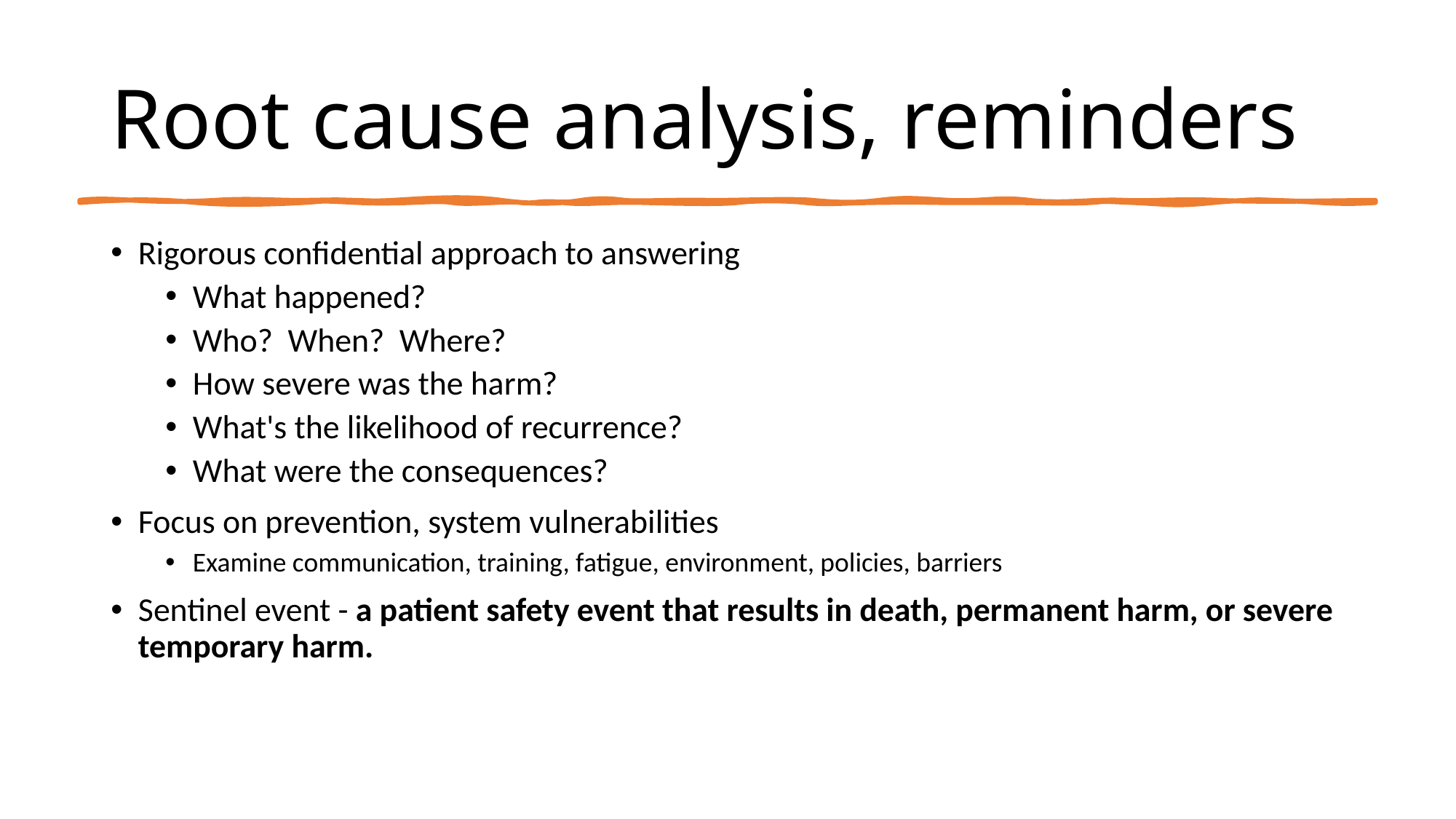

# Root cause analysis, reminders
Rigorous confidential approach to answering
What happened?
Who?  When?  Where?
How severe was the harm?
What's the likelihood of recurrence?
What were the consequences?
Focus on prevention, system vulnerabilities
Examine communication, training, fatigue, environment, policies, barriers
Sentinel event - a patient safety event that results in death, permanent harm, or severe temporary harm.

## Slide 14
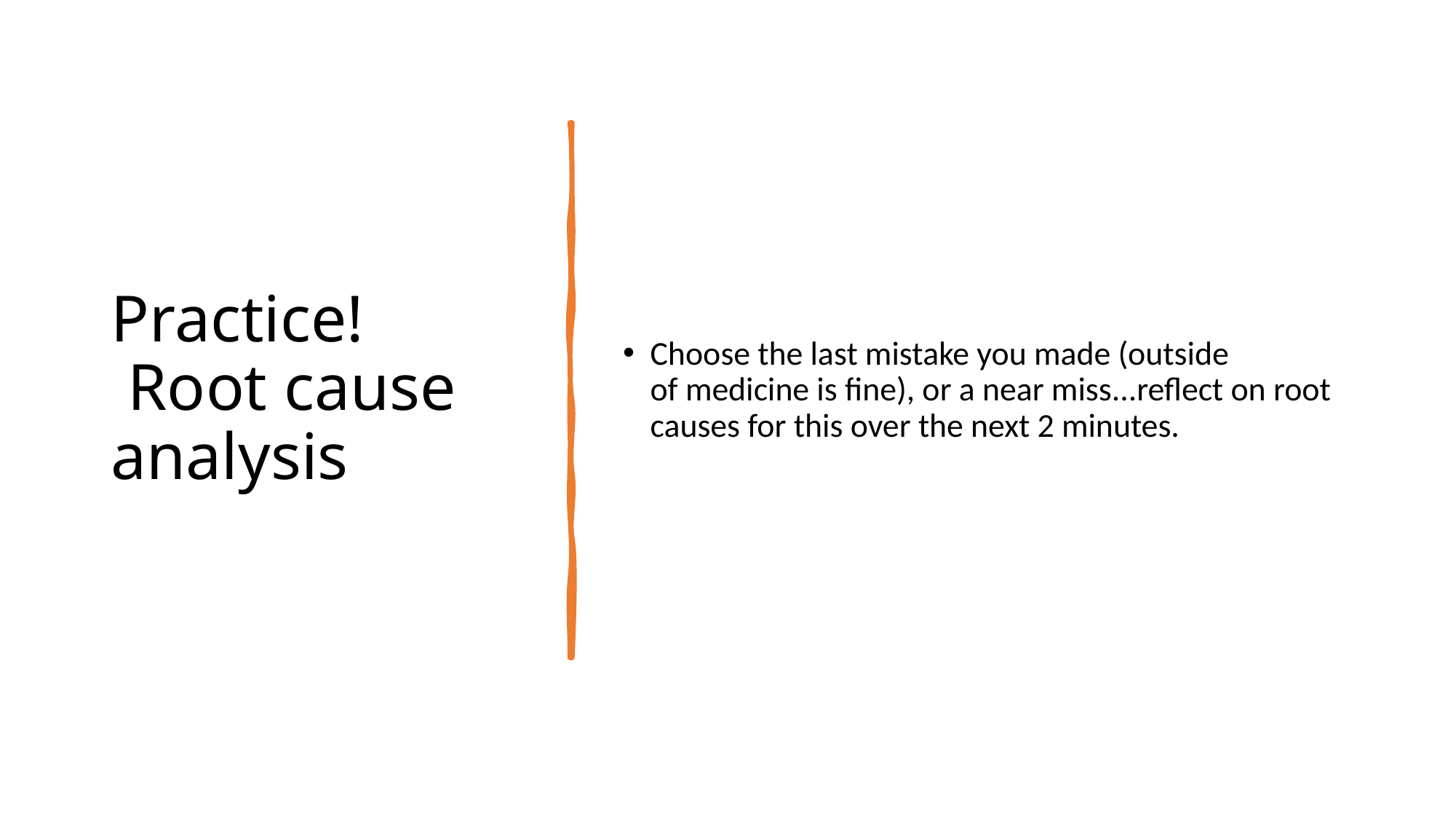

# Practice!   Root cause analysis
Choose the last mistake you made (outside of medicine is fine), or a near miss...reflect on root causes for this over the next 2 minutes.

## Slide 15
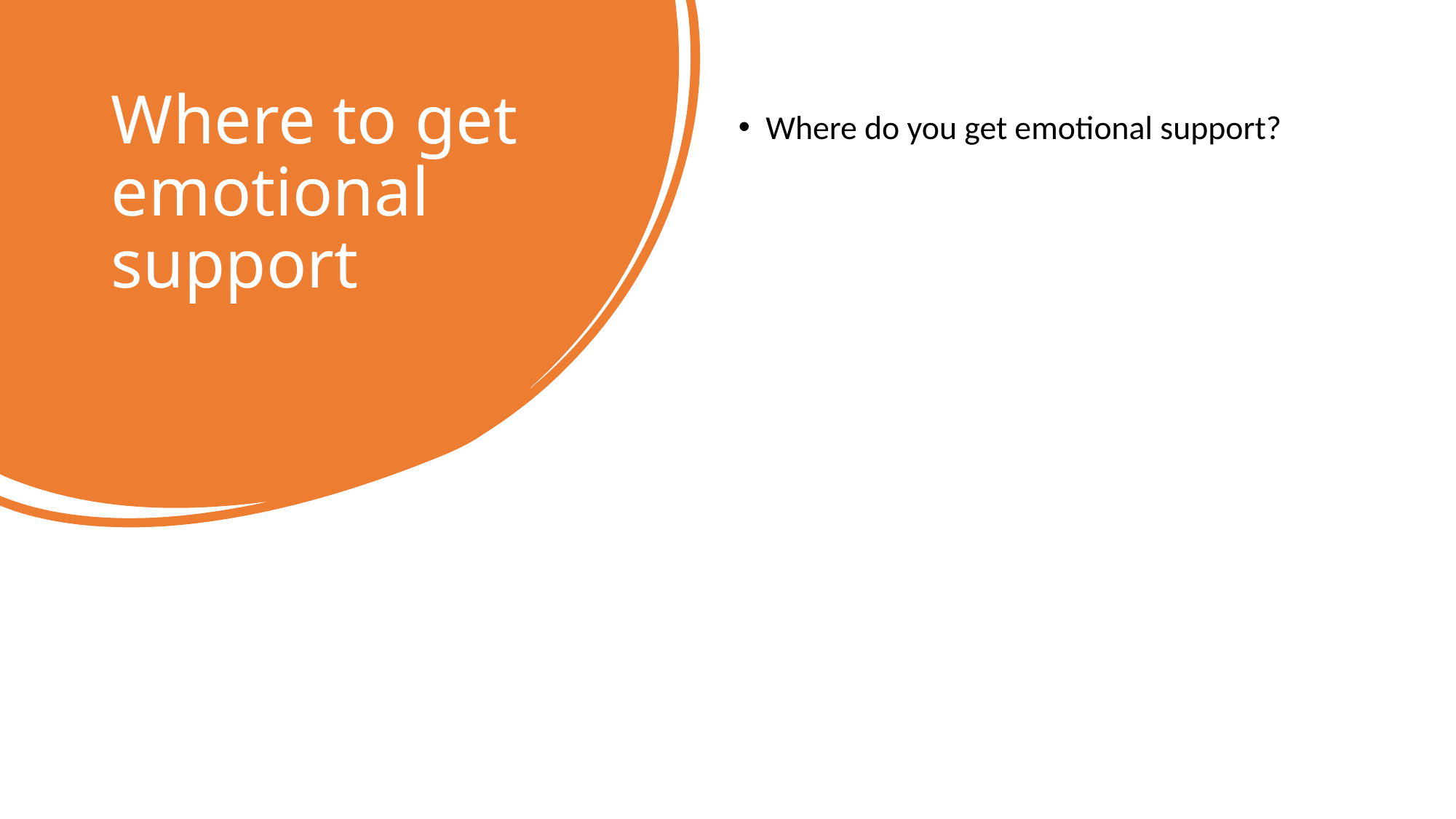

# Where to get emotional support
Where do you get emotional support?

## Slide 16
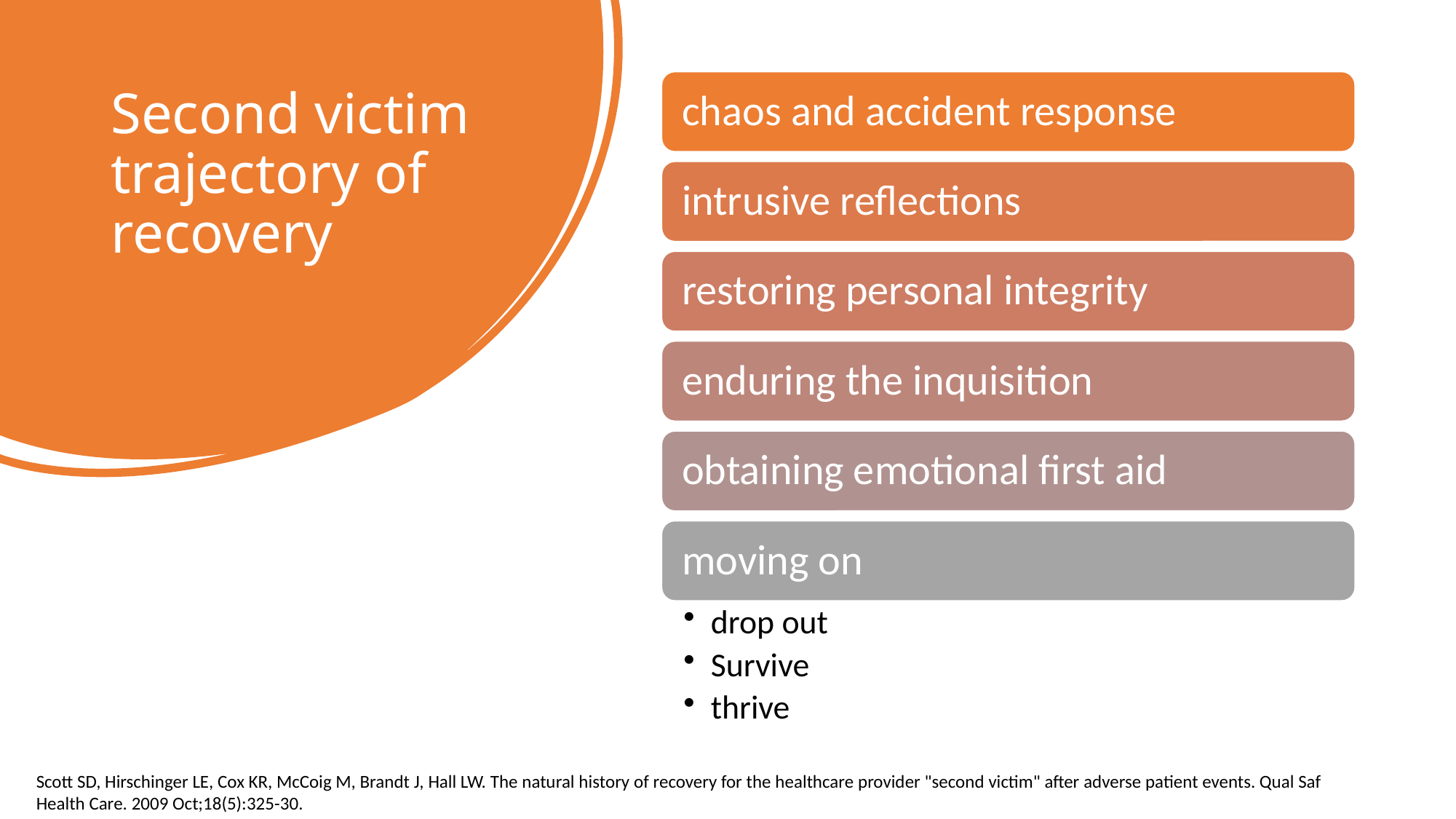

# Second victim trajectory of recovery
Scott SD, Hirschinger LE, Cox KR, McCoig M, Brandt J, Hall LW. The natural history of recovery for the healthcare provider "second victim" after adverse patient events. Qual Saf Health Care. 2009 Oct;18(5):325-30.

## Slide 17
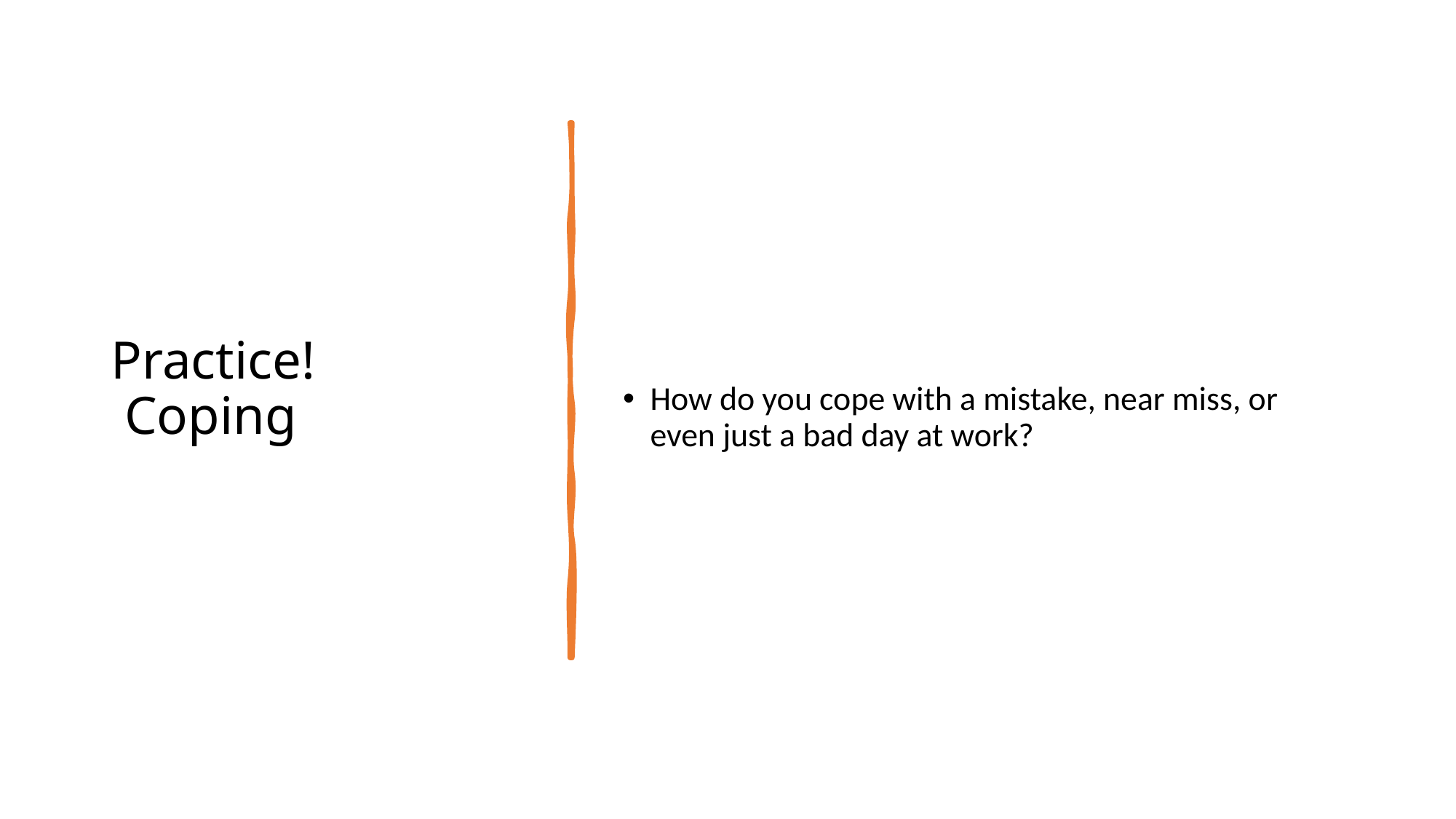

# Practice!   Coping
How do you cope with a mistake, near miss, or even just a bad day at work?

## Slide 18
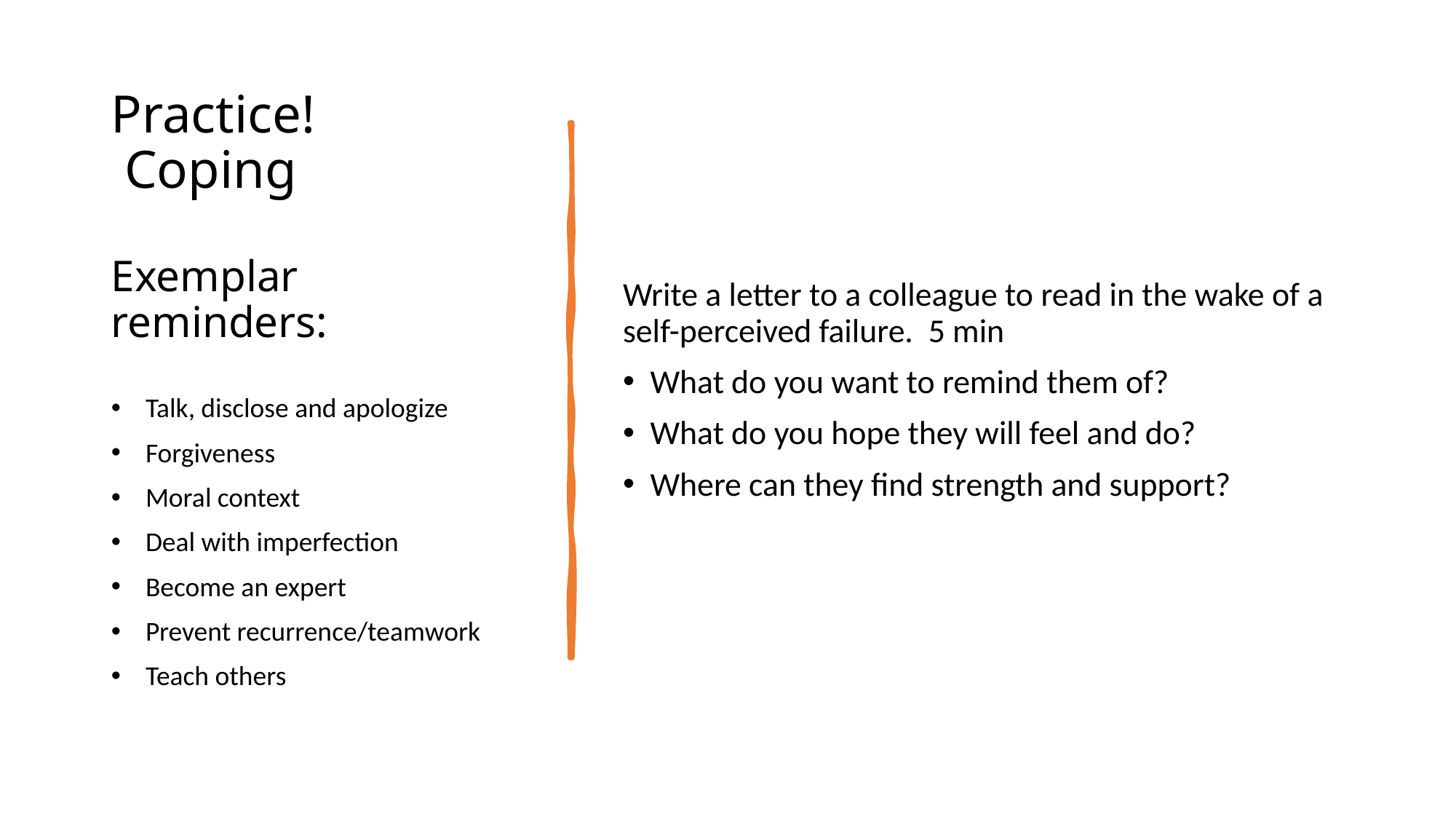

# Practice!   Coping   Exemplar reminders:
Talk, disclose and apologize
Forgiveness
Moral context
Deal with imperfection
Become an expert
Prevent recurrence/teamwork
Teach others
Write a letter to a colleague to read in the wake of a self-perceived failure.  5 min
What do you want to remind them of?
What do you hope they will feel and do?
Where can they find strength and support?

## Slide 19
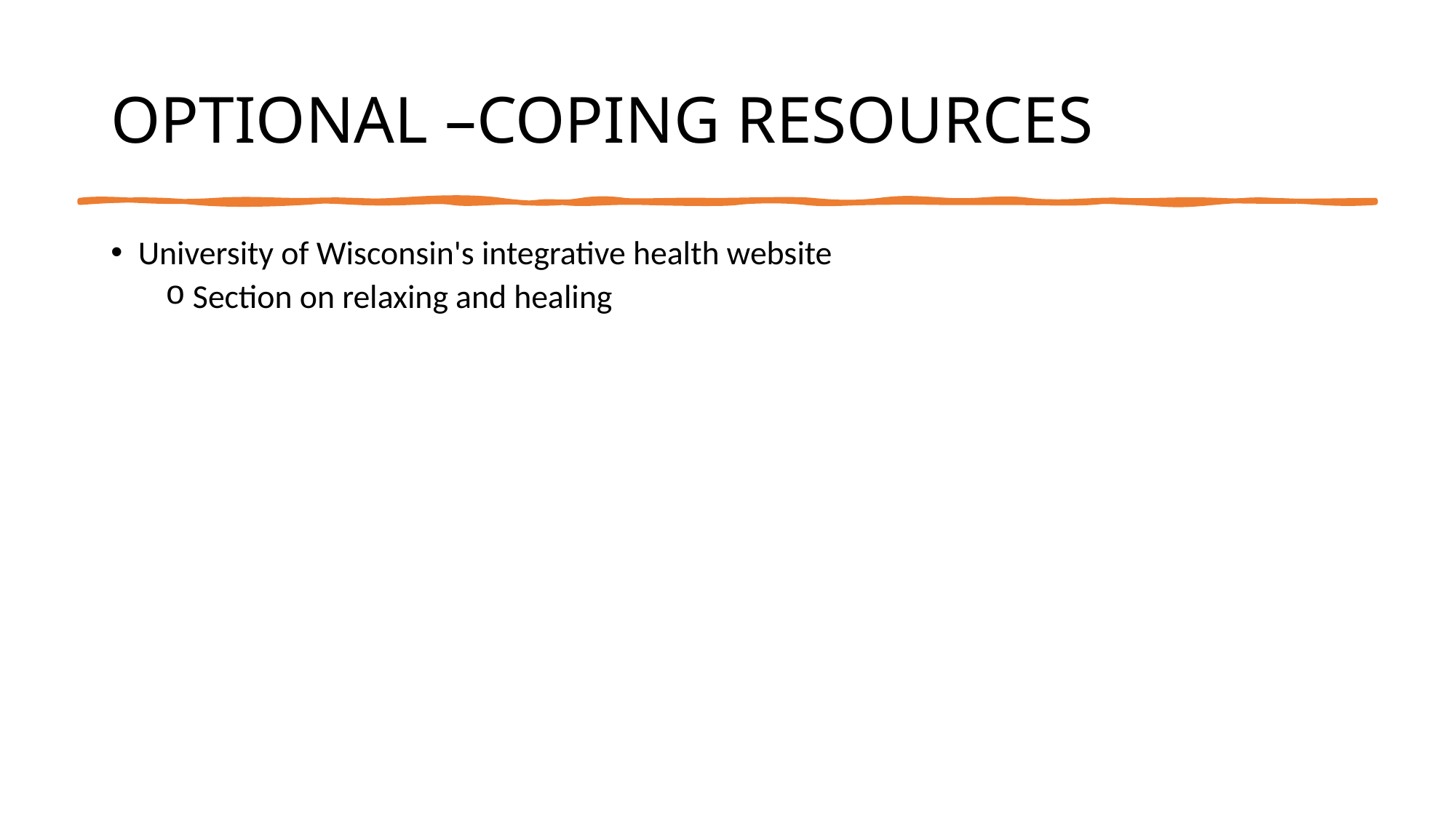

# OPTIONAL –COPING RESOURCES
University of Wisconsin's integrative health website
Section on relaxing and healing

## Slide 20
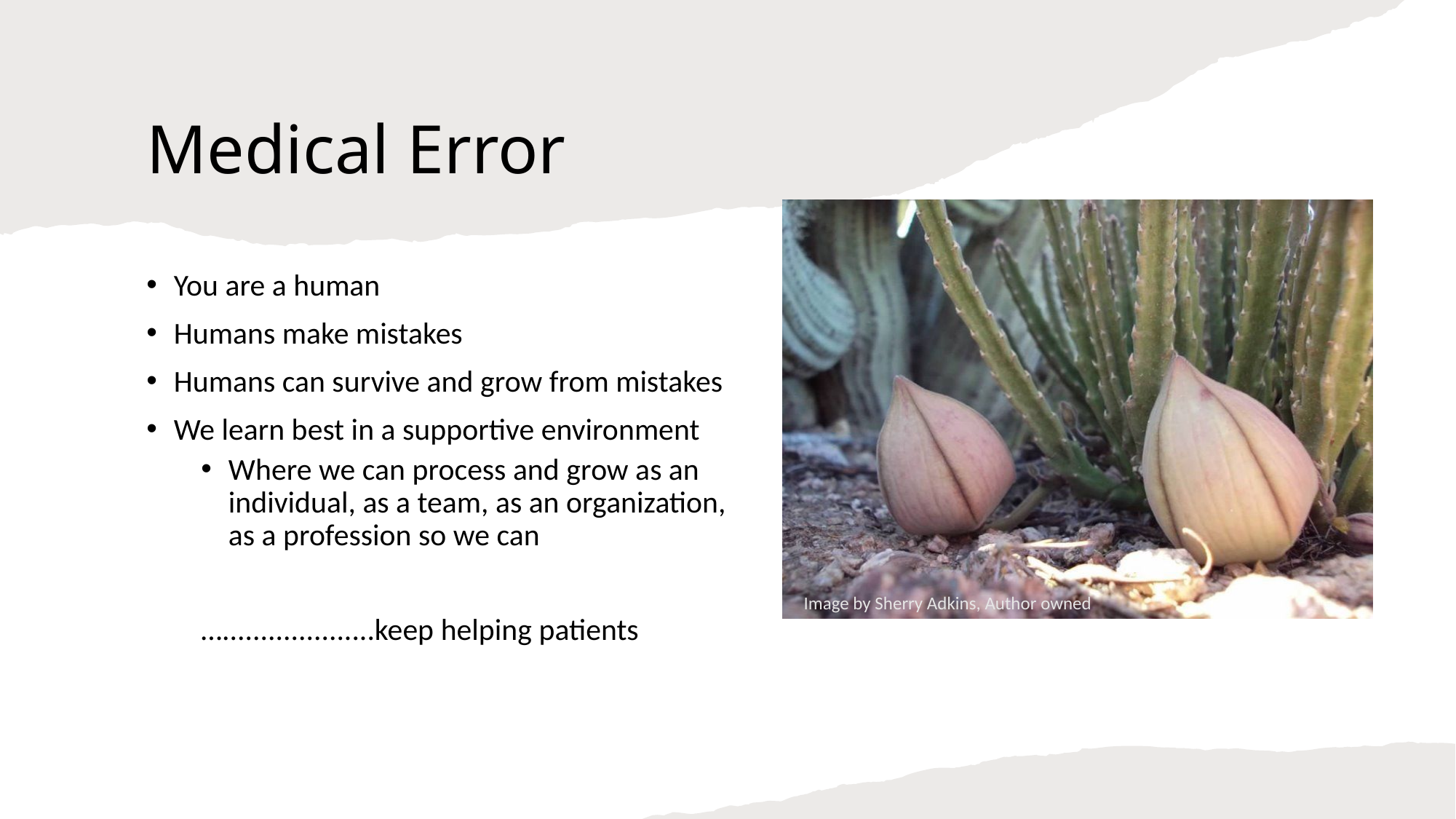

# Medical Error
You are a human
Humans make mistakes
Humans can survive and grow from mistakes
We learn best in a supportive environment
Where we can process and grow as an individual, as a team, as an organization, as a profession so we can
…....................keep helping patients
Image by Sherry Adkins, Author owned

## Slide 21
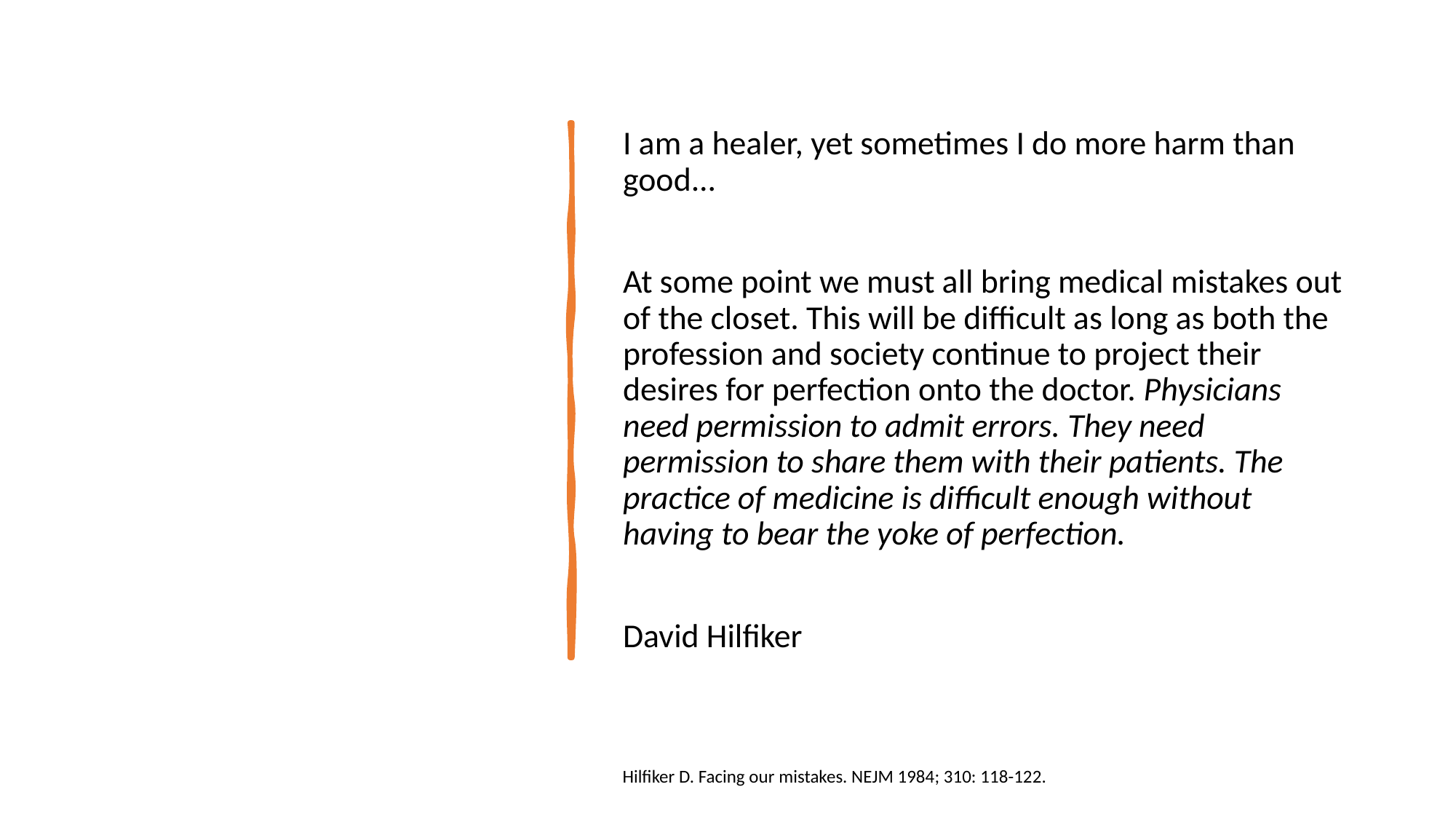

I am a healer, yet sometimes I do more harm than good...
At some point we must all bring medical mistakes out of the closet. This will be difficult as long as both the profession and society continue to project their desires for perfection onto the doctor. Physicians need permission to admit errors. They need permission to share them with their patients. The practice of medicine is difficult enough without having to bear the yoke of perfection.
David Hilfiker
Hilfiker D. Facing our mistakes. NEJM 1984; 310: 118-122.

## Slide 22
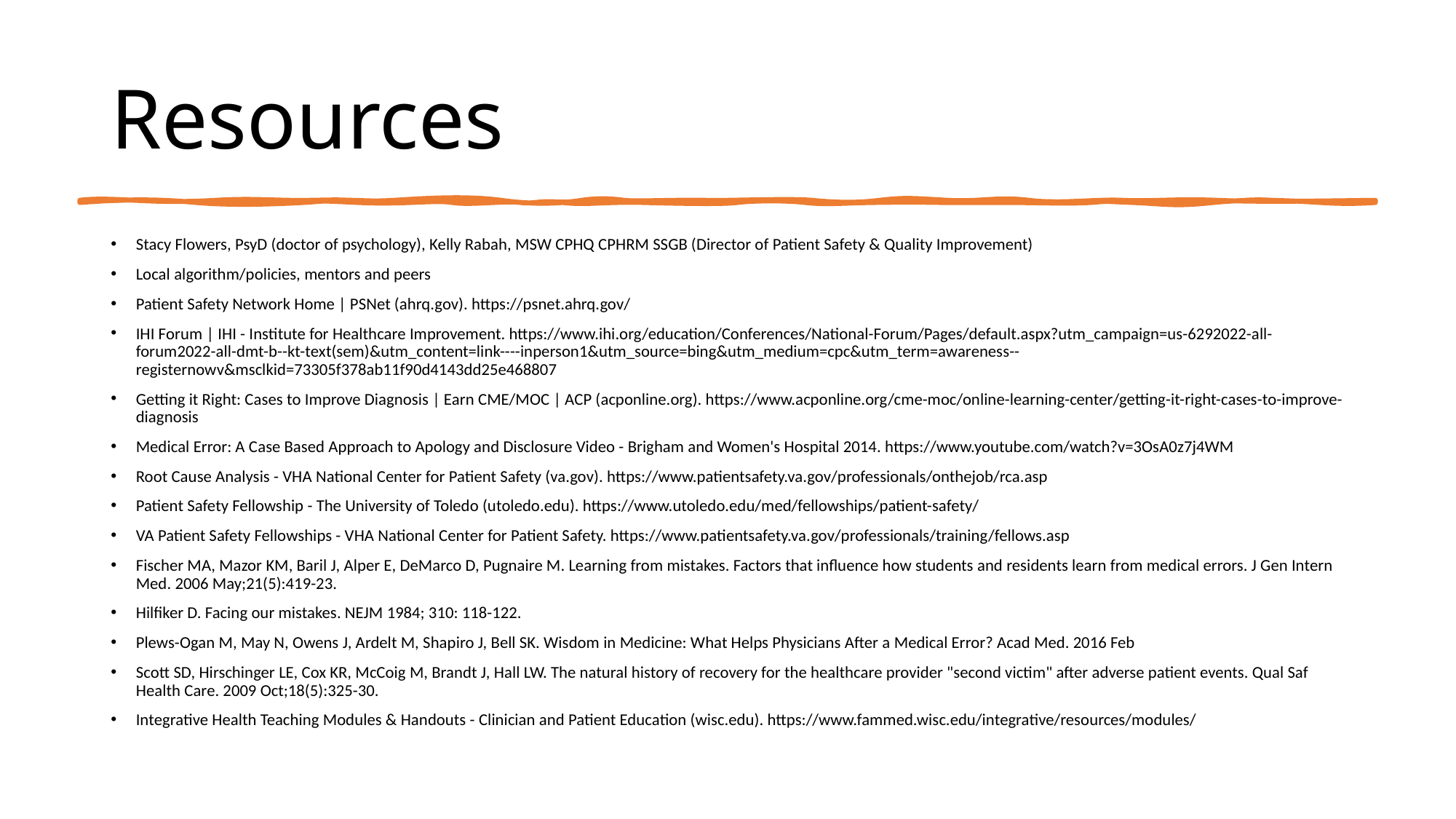

# Resources
Stacy Flowers, PsyD (doctor of psychology), Kelly Rabah, MSW CPHQ CPHRM SSGB (Director of Patient Safety & Quality Improvement)
Local algorithm/policies, mentors and peers
Patient Safety Network Home | PSNet (ahrq.gov). https://psnet.ahrq.gov/
IHI Forum | IHI - Institute for Healthcare Improvement. https://www.ihi.org/education/Conferences/National-Forum/Pages/default.aspx?utm_campaign=us-6292022-all-forum2022-all-dmt-b--kt-text(sem)&utm_content=link----inperson1&utm_source=bing&utm_medium=cpc&utm_term=awareness--registernowv&msclkid=73305f378ab11f90d4143dd25e468807
Getting it Right: Cases to Improve Diagnosis | Earn CME/MOC | ACP (acponline.org). https://www.acponline.org/cme-moc/online-learning-center/getting-it-right-cases-to-improve-diagnosis
Medical Error: A Case Based Approach to Apology and Disclosure Video - Brigham and Women's Hospital 2014. https://www.youtube.com/watch?v=3OsA0z7j4WM
Root Cause Analysis - VHA National Center for Patient Safety (va.gov). https://www.patientsafety.va.gov/professionals/onthejob/rca.asp
Patient Safety Fellowship - The University of Toledo (utoledo.edu). https://www.utoledo.edu/med/fellowships/patient-safety/
VA Patient Safety Fellowships - VHA National Center for Patient Safety. https://www.patientsafety.va.gov/professionals/training/fellows.asp
Fischer MA, Mazor KM, Baril J, Alper E, DeMarco D, Pugnaire M. Learning from mistakes. Factors that influence how students and residents learn from medical errors. J Gen Intern Med. 2006 May;21(5):419-23.
Hilfiker D. Facing our mistakes. NEJM 1984; 310: 118-122.
Plews-Ogan M, May N, Owens J, Ardelt M, Shapiro J, Bell SK. Wisdom in Medicine: What Helps Physicians After a Medical Error? Acad Med. 2016 Feb
Scott SD, Hirschinger LE, Cox KR, McCoig M, Brandt J, Hall LW. The natural history of recovery for the healthcare provider "second victim" after adverse patient events. Qual Saf Health Care. 2009 Oct;18(5):325-30.
Integrative Health Teaching Modules & Handouts - Clinician and Patient Education (wisc.edu). https://www.fammed.wisc.edu/integrative/resources/modules/
