# Supplementary material for: Medical Error: Using Storytelling and Reflection to Impact Resident Error Response Factors
Source: MedEdPORTAL. 2024 Oct 10;20:11451. doi: 10.15766/mep_2374-8265.11451 (PMC11466310; doi:10.15766/mep_2374-8265.11451)
Supplement: Supplementary file 1 — Facilitators Guide.docxError Session 1.pptxError Session 1 Handout.pdfError Session 2.pptxError Session 3.pptxError Session 3 Handout - Error Cases.docxFaculty Survey.docxPremodule Resident Survey.docxPostmodule Resident Survey.docx [file mep_2374-8265.11451-s001.zip › F. Error Session 3 Handout - Error Cases.docx]

Session 3

Roleplay/Small-Group Discussion

Clinical error situations

1. You are seeing a patient in follow up and on reviewing a consultant’s note, you see mention of a lung mass noted on chest x-ray from approximately 6 months ago. The patient tells you they never knew about this finding. The x-ray was ordered by a specialist as part of their preop evaluation.
2. You receive an ultrasound report for a patient of yours that shows possible malposition of IUD, which you placed approximately 6 months prior. The patient reported pain during the procedure, but this did not seem out of proportion. She began to experience pelvic pain about a month ago.
3. You are seeing your patient in follow up after excision of a sebaceous cyst on the back, and their sutures have failed. After completing the procedure, you reflected perhaps you didn’t undermine the tissue enough and may have needed a deep layer of sutures to make the closure stronger.
4. You are notified of a patient’s death on the inpatient hospice service, from a presumed myasthenia gravis crisis. You had completed an extensive evaluation for fatigue and shortness of breath, which were progressive over the last six months. Most recently, you requested the patient see neurology, but the patient was admitted with respiratory failure before the specialist’s visit. You are seeing the patient’s son now for his own follow up visit.
5. EMS has just left your facility, after a post-vaccine syncopal episode resulted in a significant head injury to a young, healthy patient. You need to alert the patient’s wife to this event.
6. You are seeing a patient with long term cognitive disability in follow up from an acute visit with a partner. The patient reported feeling tired, elevated heart rate was noted (ST on EKG), patient was referred to cardiology and started on a beta blocker. You do further evaluation and note he is anemic.
7. You receive notice from your office staff that a patient of yours is angry that you did not check on her while she was in the hospital with COVID-19 and complications. She feels that you don’t care and she doesn’t want to return to see you. How do you navigate what the patient sees as an error or deficiency in care?
