# Supplementary material for: Medical Error: Using Storytelling and Reflection to Impact Resident Error Response Factors
Source: MedEdPORTAL. 2024 Oct 10;20:11451. doi: 10.15766/mep_2374-8265.11451 (PMC11466310; doi:10.15766/mep_2374-8265.11451)
Supplement: Supplementary file 1 — Facilitators Guide.docxError Session 1.pptxError Session 1 Handout.pdfError Session 2.pptxError Session 3.pptxError Session 3 Handout - Error Cases.docxFaculty Survey.docxPremodule Resident Survey.docxPostmodule Resident Survey.docx [file mep_2374-8265.11451-s001.zip › G. Faculty Survey.docx]

Faculty and Medical Error Curriculum

**This questionnaire is designed to find out about the current curriculum around medical error and faculty stories that can help learners develop their understanding of error and personal plan for reducing and managing error. Please avoid identifying comments in your free text answers.**

**If you would like to share a story of a medical error during your career for learning purposes (could be de-identfiied, shared by another faculty, or told personally by you), please contact (insert your contact).**

**If you are personally struggling to process a medical error, please contact (insert your contact).**

**Please answer the following questions with strongly agree, agree, neutral, disagree, or strongly disagree.**

Strongly agree Agree Neutral Disagree Strongly

disagree


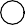

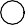

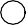

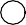

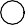
Good doctors should be honest about errors they make.


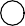

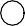

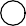

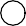
If I am smart enough, I can avoid
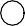
 medical error for myself and my

patients.


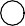

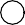


I have made errors in my care for patients. Yes No

Patient-physician relationships can recover after medical error.

Physicians can recover after medical error.

I can recover after a medical error.

I can be honest about errors that I make as a doctor.

Strongly agree Agree Neutral Disagree Strongly

disagree

**Please answer the following questions with strongly agree, agree, neutral, disagree, or**

**strongly disagree.**


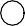

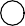

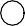

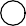

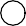

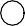

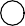

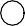

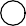

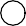

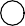

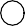

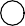

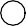

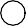

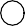

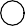

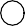

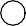

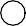


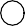

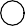

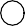

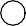

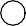

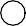
I acknowledge when I am at increased risk for making errors (i.e. hungry, angry, late, tired, inexperienced/unfamiliar).

Have you shared a story with a learner about a Yes

personal medical error? No


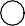

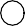


Have you shared a story with a peer about a personal Yes

medical error? No


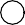

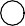


Have you personally been part of a patient care team Yes

where the patient experienced medical error? No


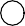

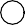

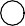

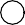

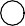

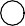


Was the error acknowledged among the care team? Yes No

How did you personally process this error? (please avoid including identifying information)

Did the team debrief or attempt to discuss root causes Yes for the error? No

How did the team process the error? (please avoid including identifying information)

Was the error disclosed to the patient? Yes No

I don't know.


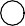

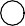

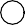


How did that happen? (please avoid including identifying information)

Did the team or organization learn from the error? Yes No

I don't know.


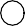

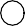

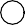


I know what to do at my institution when faced with a Yes

medical error (I am aware of their policies related to No this topic).


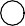

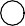


What do you do when you become aware of a medical error? (Select all that apply.)

Hide it/pretend it didn't happen

Acknowledge it personally and try to learn but do not discuss with others

Discuss informally with peers but do not engage faculty or patient.

Work to debrief formally with the team and faculty to learn from the error.

Share openly about the error so others can learn. Feel bad about myself

Reach out to others to process the error Other

You indicated "Other" when you become aware of a medical error. If you are willing, please share what this means for you.


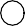

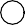


Have you had training in how to handle the occurrence Yes of a medical error? No

Do you desire more training in any of the following?
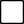
 Error management


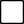
 Quality improvement


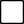
 Error disclosure to peers and patients
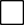
 Safety culture


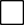
 Personal stories of error from mentors


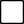
 Culture of safety (versus culture of blame)


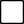
 Legal and malpractice concerns related to medical error


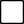
 Personal coping


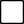
 Patient stories of error
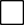
 Other

What additional training would you be interested in?

**Please answer the following questions with strongly agree, agree, neutral, disagree, or**

**strongly disagree.**

Strongly agree Agree Neutral Disagree Strongly

disagree


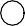

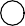

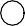

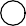

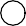
I am able to share stories of error from my career with learners.

I am not able to share stories of error from my career because
