# Supplementary material for: Medical Error: Using Storytelling and Reflection to Impact Resident Error Response Factors
Source: MedEdPORTAL. 2024 Oct 10;20:11451. doi: 10.15766/mep_2374-8265.11451 (PMC11466310; doi:10.15766/mep_2374-8265.11451)
Supplement: Supplementary file 1 — Facilitators Guide.docxError Session 1.pptxError Session 1 Handout.pdfError Session 2.pptxError Session 3.pptxError Session 3 Handout - Error Cases.docxFaculty Survey.docxPremodule Resident Survey.docxPostmodule Resident Survey.docx [file mep_2374-8265.11451-s001.zip › I. Postmodule Resident Survey.docx]

Residents and Medical Error Curriculum Post-Survey

Please complete the survey below. Thank you!

| **This questionnaire is designed to find out about the current formal and informal curriculum around medical error and resident interest in further studies. Please avoid identifying comments in your free text answers.**  **If you are personally struggling to process a medical error, please use the contact info provided during our session to reach out.** | | | | |
| --- | --- | --- | --- | --- |
| Strongly agree | Agree | Neutral | Disagree | Strongly disagree |
| Good doctors should be honest about errors they make. 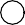 | 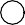 | 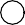 | 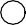 | 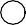 |
| Patient-physician relationships can recover after medical error. 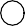 | 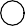 | 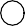 | 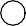 | 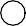 |
| Physicians can recover after medical error. 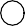 | 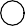 | 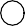 | 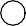 | 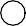 |
| I can recover after a medical error. 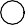 | 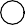 | 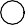 | 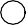 | 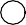 |
| If I am smart enough, I can avoid 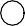 medical error for myself and my  patients. | 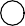 | 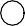 | 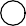 | 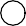 |
| My mentors have made errors in their care for patients. | Yes No Not 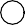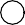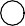 |  |  |  |
|  |  | sure |  |  |
| Strongly agree | Agree | Neutral | Disagree | Strongly |
|  |  |  |  | disagree |
| I can be honest about errors that 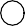 I make as a doctor. | 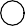 | 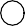 | 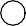 | 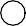 |
| I acknowledge when I am at increased risk for making errors (i.e. hungry, angry, late, tired, inexperienced/unfamiliar). 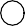 | 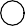 | 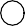 | 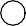 | 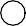 |
| Have you had a mentor share a story about a personal medical error? | Yes No 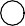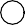 |  |  |  |
| Have you had a peer share a story about a personal medical error? | Yes No 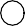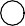 |  |  |  |
| I know what to do at my institution when faced with a  medical error (I am aware of their policies related to this topic). | Yes No 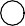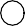 |  |  |  |

How many sessions of medical error training with Dr. None


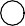

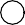

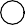

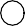


Adkins have you attended? 1

2

3

On a scale of 0-10 (0 being not emotionally difficult at all to 10 extremely emotionally difficult) how emotionally difficult was this training?

On a scale of 0-10 (0 being not feasible at all and 10 being extremely feasible) how feasible was it to complete this training?

On a scale of 0-10 (0 being not helpful at all and 10 being extremely helpful) how helpful was this training?

0 1 2 3 4 5 6 7 8 9 10


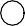

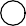

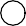

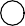

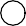

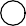

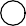

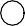

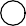

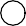

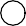

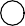


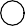

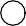

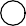

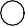

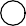

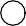

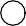

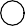

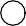

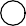

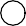

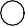


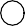

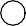

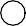

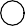

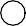

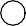

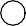

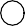

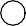

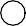

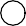

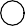


What do you do when you become aware of a medical error?

Hide it/pretend it didn't happen

Acknowledge it personally and try to learn but do not discuss with others

Discuss informally with peers but do not engage faculty or patient.

Work to debrief formally with the team and faculty to learn from the error.

Share openly about the error so others can learn. Feel bad about myself

Reach out to others to process the error Other.

You indicated "Other" when you become aware of a medical error. If you are willing, please share what this means for you.


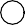

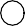


Have you had training in how to handle the occurrence Yes of a medical error? No

Do you desire more training in any of the following?
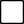
 Error management


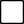
 Quality improvement


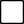
 Error disclosure to peers and patients
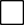
 Safety culture


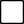
 Personal stories of error from mentors


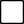
 Culture of safety (versus culture of blame)


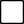
 Legal and malpractice concerns related to medical error


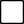
 Personal coping


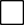
 Patient stories of error
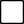
 Other

What additional training would you be interested in?

I have additional feedback for the presenter

Do you have any feedback or suggestions about the training curriculum?
